# The effect of antibiotics on the intestinal microbiota in children - a systematic review

**DOI:** 10.3389/falgy.2024.1458688

**Published:** 2024-10-07

**Authors:** Juliane Wurm, Nigel Curtis, Petra Zimmermann

**Affiliations:** ^1^ Department of Paediatrics, Fribourg Hospital, Fribourg, Switzerland; ^2^ Department of Health Science and Medicine, University Lucerne, Lucerne, Switzerland; ^3^ Department of Paediatrics, The University of Melbourne, Parkville, VIC, Australia; ^4^ Infectious Diseases Research Group, Murdoch Children’s Research Institute, Parkville, VIC, Australia; ^5^ Infectious Diseases Unit, The Royal Children’s Hospital Melbourne, Parkville, VIC, Australia; ^6^ Department for Community Health, Faculty of Science and Medicine, University of Fribourg, Fribourg, Switzerland

**Keywords:** microbiome, stool, intestine, sequencing, 16S rRNA, penicillin, cephalosporin, macrolide

## Abstract

**Background:**

Children are the age group with the highest exposure to antibiotics (ABX). ABX treatment changes the composition of the intestinal microbiota. The first few years of life are crucial for the establishment of a healthy microbiota and consequently, disturbance of the microbiota during this critical period may have far-reaching consequences. In this review, we summarise studies that have investigated the effect of ABX on the composition of the intestinal microbiota in children.

**Methods:**

According to the PRISMA guidelines, a systematic search was done using MEDLINE and Embase to identify original studies that have investigated the effect of systemic ABX on the composition of the intestinal microbiota in children.

**Results:**

We identified 89 studies investigating a total of 9,712 children (including 4,574 controls) and 14,845 samples. All ABX investigated resulted in a reduction in alpha diversity, either when comparing samples before and after ABX or children with ABX and controls. Following treatment with penicillins, the decrease in alpha diversity persisted for up to 6–12 months and with macrolides, up to the latest follow-up at 12–24 months. After ABX in the neonatal period, a decrease in alpha diversity was still found at 36 months. Treatment with penicillins, penicillins plus gentamicin, cephalosporins, carbapenems, macrolides, and aminoglycosides, but not trimethoprim/sulfamethoxazole, was associated with decreased abundances of beneficial bacteria including Actinobacteria, *Bifidobacteriales*, *Bifidobacteriaceae,* and/or *Bifidobacterium*, and *Lactobacillus.* The direction of change in the abundance of *Enterobacteriaceae* varied with ABX classes, but an increase in *Enterobacteriaceae* other than *Escherichia coli* was frequently observed.

**Conclusion:**

ABX have profound effects on the intestinal microbiota of children, with notable differences between ABX classes. Macrolides have the most substantial impact while trimethoprim/sulfamethoxazole has the least pronounced effect.

## Introduction

Children are the age group with the highest exposure to antibiotics (ABX). ABX are the second most prescribed drugs for children, surpassed only by analgesics (1–5). More than two-thirds of children receive ABX before reaching the age of two years (6) with exposure to an average of almost three ABX in the first year of life (7). Approximately one third of hospitalised children (8) and nearly half of acutely ill children in outpatient settings receive ABX (9), often with inappropriate indications or drugs (10). While the widespread use of ABX has significantly reduced childhood morbidity and mortality during the last century (11), exposure to ABX is also associated with adverse long-term health effects. These include an increased risk for atopic dermatitis, allergies, wheezing and asthma, obesity, arthritis idiopathic disorder, psoriasis, and neurodevelopmental disorders (12). These adverse effects likely result from changes in the microbiota, particularly the intestinal microbiota, which undergoes significant development during the first two to three years of life (13–15). Children are highly susceptible to ABX-induced dysbiosis (an imbalance in the microbiota), whereas adults typically have a more stable and resilient microbiota that recovers more easily from such disturbances (16). The first years of life are critical for the development and stabilisation of the intestinal microbiota, coinciding with key milestones in the development of the immune system, metabolism and neurodevelopment (17–19). Consequently, any disturbances of the microbiota during this critical period may have long-lasting and far-reaching consequences.

In this review, we systematically summarise studies that have investigated the effect of ABX on the composition of the intestinal microbiota in children of all age groups.

## Methods

In May 2024, MEDLINE (1946 to present) and Embase (1972 to present) were searched. Embase was searched using the OVID interface. The detailed search terms can be found in the supplementary data. No geographical limitations were used. References of retrieved articles were searched for additional publications.

Original studies which investigated effect of ABX on the composition of the bacterial intestinal microbiota in children less than 18 years of age were included, as well as studies involving mixed-age populations that provided separate data specifically for this age group. Exclusion criteria were studies which (i) included children with underlying diseases (e.g., oncological diseases, cystic fibrosis) and (ii) studies not published in English, German, French, Spanish, Portuguese or Italian.

The following variables were extracted from included studies: year of publication, country, study design, number and characteristics of included children, number of samples, ABX treatment (drug, dose, frequency, route of administration, duration), previous ABX, microbiota analysis method, timing of stool analysis and key findings (including changes in diversity, abundance of microbes, antibiotic resistance genes, ARGs).

Studies were identified, selected, appraised, and synthesised following the Preferred Reporting Items for Systematic Reviews and Meta-Analyses (PRISMA) guidelines for systematic reviews (20, 21). The level of evidence of each study was classified according to the 2011 Oxford Centre for Evidence-Based Medicine (OCEBM) Levels of Evidence (22). Risk of bias was assessed using the 2017 Joanna Briggs Institution (JBI) standardised critical appraisal checklist for case-control and cohort studies (23).

## Results

**Figure 1:**
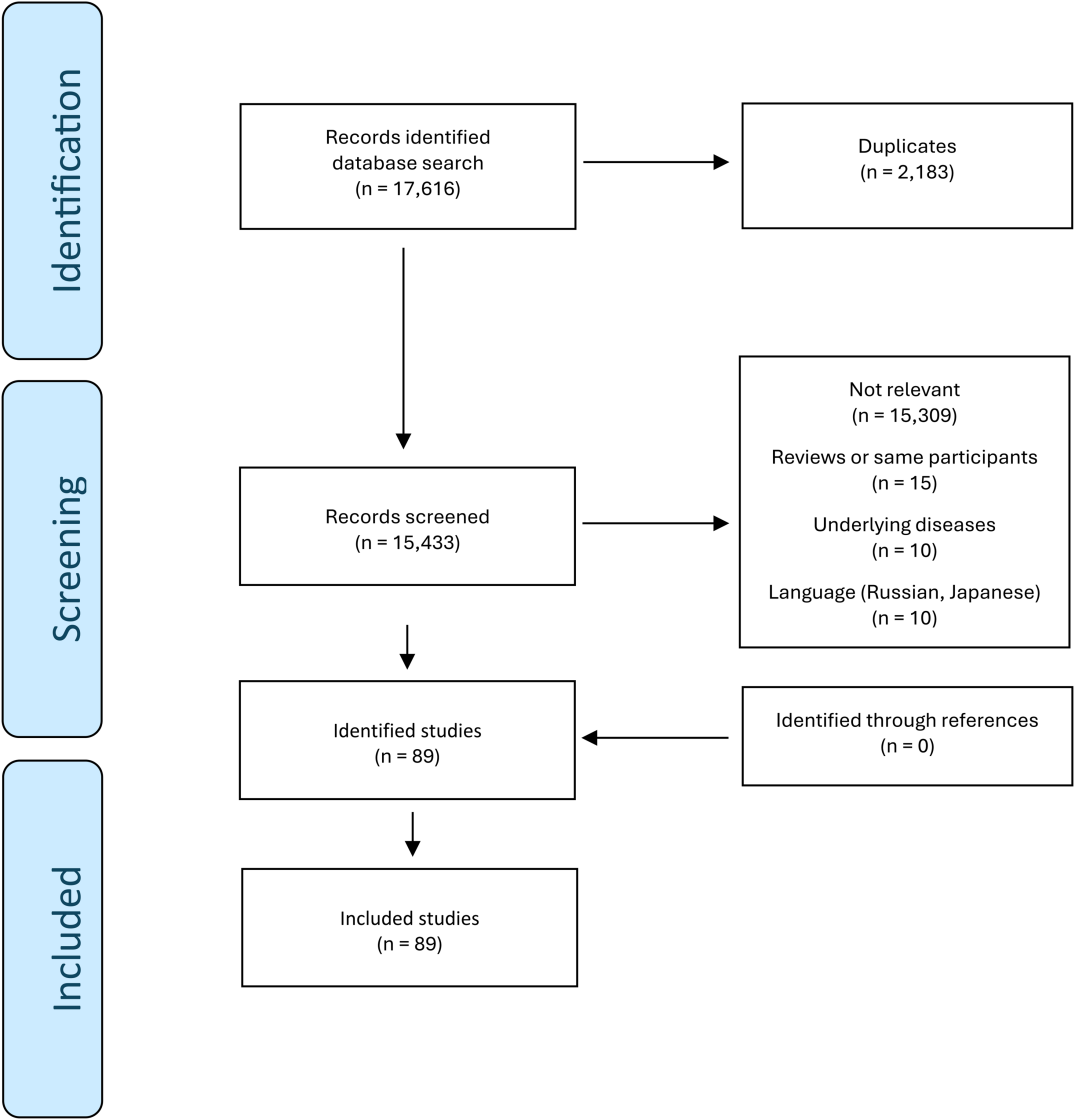
Selection of studies.

The search identified 6,146 and 11,470 studies in MEDLINE and Embase, respectively. From the 17,616 studies, 2,183 duplicates were removed. 89 studies met the inclusion criteria (24–112). No additional relevant studies were found through citation searching. The selection of included studies is summarized in Figure 1.

The 89 studies investigated a total of 9,712 children (of these 4,574 were controls; mean 111 children per study, range 9 to 1,023) and 14,845 samples (mean 322 samples per study, range 20 to 1,247). Of the included studies, 47 were done in Europe (Denmark, Netherlands, Finland, Spain, France, Italy, Germany, Ireland, Estonia, United Kingdom, Sweden, Norway, Austria) (24, 26, 28, 29, 31, 32, 34, 35, 37–39, 43, 45, 48–52, 60, 61, 63, 64, 66, 67, 70, 71, 74, 75, 81–83, 88, 92, 93, 95, 98, 101, 102, 104–112), 21 on the American continent (USA, Chile, Canada) (25, 30, 41, 42, 44, 54–56, 58, 59, 69, 73, 76, 78, 84–86, 94, 96, 97, 99), 13 in Asia (China, India, Japan, Korea, Taiwan, Lebanon) (27, 40, 53, 57, 65, 68, 72, 77, 80, 87, 90, 91, 100), seven in Africa (Niger, Burkina Faso, Zimbabwe, South Africa) (36, 46, 47, 62, 79, 89, 103) and one in Australia (33). The age of participants ranged from preterm birth to 15 years. Of the included studies, 75 were observational studies, including 60 cohort studies (24, 25, 27, 28, 30–35, 37–42, 44, 45, 48–58, 61, 64–68, 70, 71, 73, 74, 76–78, 84, 87–92, 94, 97–102, 109–112), 12 cross-sectional studies (26, 29, 43, 72, 75, 82, 85, 93, 95, 99, 108, 113), three pre-post-intervention studies (35, 60, 96); and 13 (cluster) randomised controlled trials (36, 46, 47, 59, 62, 63, 69, 79–81, 83, 86, 103). In total, 42 studies used 16S rRNA gene sequencing (25–28, 40–46, 48, 51, 52, 55, 57, 58, 62, 64, 66, 68, 69, 71–73, 76, 77, 79, 80, 83–86, 91, 92, 98–103, 113), ten shotgun metagenomic sequencing (29, 30, 36, 47, 54, 75, 83, 89, 94, 98), seven polymerase chain reaction (PCR) or PCR-temperature gradient gel electrophoresis (PCR-TGGE) (39, 49, 50, 65, 67, 78, 82), one each fluorescence *in situ* hybridization (FISH) (104) and 16S-23S IS profiling (48), and 30 cultures (24, 31–35, 37–39, 49, 53, 56, 59–61, 63, 74, 81, 87, 88, 90, 93, 96, 105, 107–112).

Supplementary Table S1 provides a summary of the main findings of the 89 studies included in this review.

All included studies had an overall risk of bias score (JBI standardised critical appraisal checklist, yes%) over 60% (acceptable quality) and 49% (44/89) of studies had an overall score ≥80% (good quality) (Supplementary Table S3). The most frequent risk of bias was attrition bias [present in 24% (21/89) studies].

## Penicillins

The effect of penicillins on the intestinal microbiota was investigated in 13 studies including 1,311 children [one study did not specify the number of children investigated (70); for detailed information see Supplementary Table S3] (35, 55, 70, 71, 75, 78, 79, 87, 89, 90, 100, 107, 112).

### Diversity

**Figure 2:**
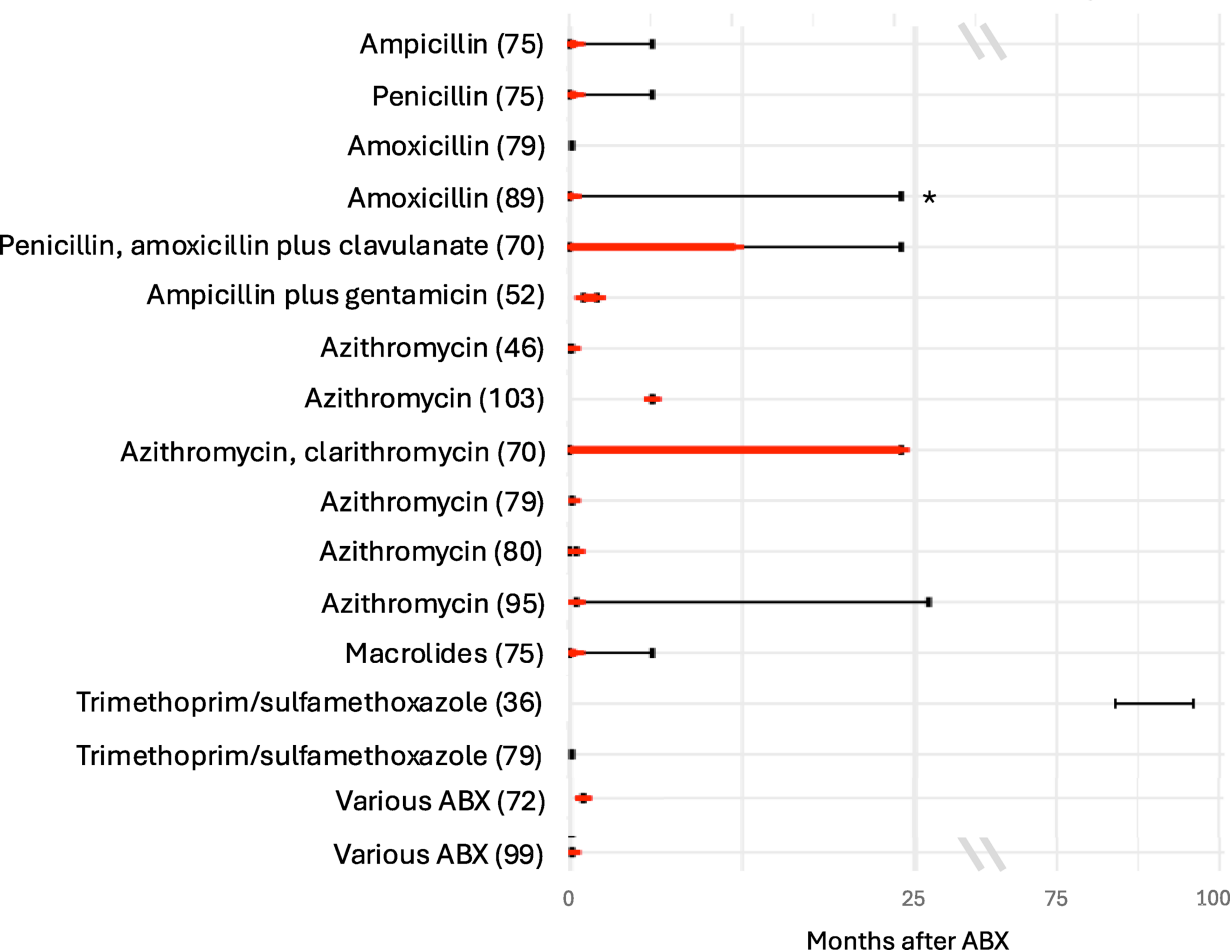
Duration of decreased alpha diversity after ABX reported in different studies. Red bars depict duration of decreased alpha diversity in ABX group compared to controls (red bars); black bars depict the duration of follow-up after ABX. Studies specifying Shannon index, inverse Simpson index or richness for the ABX and control group were included. Studies which did not specify the time points when the alpha diversity was investigated were excluded.

Two studies found a lower alpha diversity (a measure of the variety and abundance of bacterial taxa within a sample) of the intestinal microbiota during the treatment with amoxicillin compared to controls with recovery either immediately after stopping ABX (89) or six months after (71), respectively. However, compared to controls, the first study found a higher alpha diversity at the latest follow-up time point 24 months after ABX (89). In other studies, compared to controls, a lower richness was found to persist for less than two weeks after treatment with ampicillin and penicillin (75), and for up to 6–12 months (latest follow-up being after 12–24 months) in a study (which did not report separate results for treatment with amoxicillin with or without clavulanate and penicillin V) (70) (Figure 2). One study found a lower alpha diversity at day seven of life compared to day three in preterm neonates after treatment with penicillin plus moxalactam or piperacillin-tazobactam, but no difference in diversity after ABX with the two treatment regimens (100). Another study found no difference in alpha diversity, at the latest follow-up point of the study, five days after treatment with amoxicillin (79). Three studies investigated beta diversity (a measure of the differences in bacterial taxa between different samples) by comparing the ABX group and controls and found a higher beta diversity after treatment with penicillin, amoxicillin/ampicillin, penicillin plus moxalactam and piperacillin-tazobactam, respectively (75, 89, 100).

### Composition

When comparing the composition of the intestinal microbiota during or after oral administration of amoxicillin, compared to controls, increased abundances of Firmicutes (71), Enterobacteriales (71), *Ruminococcaceae* (71), *Lachnospiraceae* (71), *Megasphaera* (71), *Coprococcus* (71), *Escherichia* (89), *Dialister* (71), *Weissela confusa* (89), *Prevotella* sp. 885 (89), *Prevotella stercorea* (89), *Holdemanella biformis* (89), *Lactobacillus animalis* (89), *Fusicatenibacter saccharivorans* (89), *Catenibacterium mitsuokai* (89), *Slackia isoflavoniconvertens* (89), *Weissella cibaria* (89), *Streptococcus macedonicus* (89), *Gemmiger formicilis* (89)*,* and *Actinomyces odontolyticus* (89)*,* and decreased abundances of *Coriobacteriaceae* (71), *Bacteroidaceae* (71), *Streptococcaceae* (71), *Lactobacillus* (89), *Bifidobacterium* (71), *Enterococcus* (71), *Streptococcus* (87, 89), *Klebsiella* (89), *Holdemanella* (89), *Dorea* (89), *Bifidobacterium bifidum* (89), and *Bifidobacterium longum* (89) were observed.

**Figure 3:**
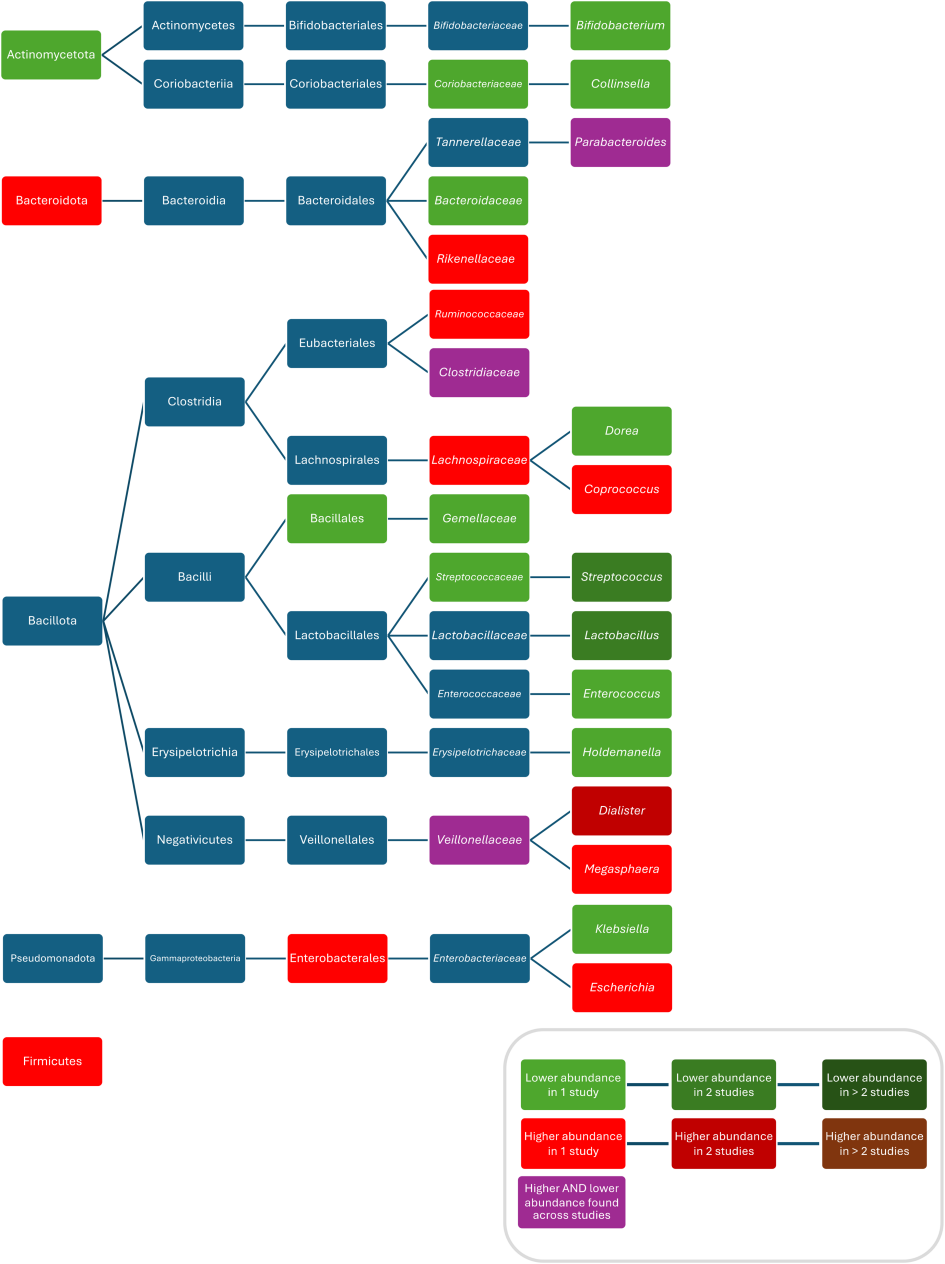
Differences in bacterial abundance between children treated with penicillins and controls. Included studies investigated amoxicillin (71, 89) and amoxicillin with or without clavulanate and penicillin V (70). Studies which did not compare ABX group to controls and studies not providing *p*-values were excluded.

When comparing the composition before and during treatment with penicillin V, ampicillin and methicillin, respectively, decreased bacterial counts of *Lactobacillus* (87), *Streptococcus* (87), and *Bifidobacterium* (87) were found. One study found increased abundances of Bacteroidetes, *Rikenellaceae,* and *Dialister* and decreased abundances of Actinobacteria, *Gemellales*, *Gemellaceae, Lactobacillus,* and *Collinsella* in children who were given penicillin or amoxicillin with or without clavulanate, without reporting results for these ABX separately and without reporting the route of administration (70). Fluctuations in abundance over time were found for *Veillonellaceae* (71), *Clostridiaceae* (both being lower during treatment with amoxicillin and higher than in controls after treatment) (71) and *Parabacteroides* (being higher within less than 6 months after treatment with amoxicillin with or without clavulanic acid or penicillin (without separate analysis) compared with controls and then being lower 6 to 12 months after treatment compared with controls (70) (Figure 3). One study found no difference in bacterial abundance after treatment with penicillin (75).

### ARGs

The effect of penicillins on the abundance of ARGs was studied in three studies. Two studies found an increased abundance of ARGs after treatment with penicillin and ampicillin/amoxicillin, respectively, which returned to normal three to four weeks after treatment (75, 89). The other study found increased abundances of multiple ARGs, e.g., for β-lactamases (unknown), ABC efflux pumps, *araC,* and *emrD* (55). A higher abundance of plasmids after treatment with penicillin or ampicillin (without separate analysis) was reported in another study (75).

## Penicillins plus aminoglycosides

13 studies investigated combinations of different penicillins plus gentamicin in 3,141 children (for detailed information see Supplementary Table S3) (26, 37, 40, 52, 58, 63, 69, 81, 83, 85, 93, 94, 110).

### Diversity

Compared with controls, three studies found a lower alpha diversity in children treated with penicillin, amoxicillin, or ampicillin plus gentamicin (52, 58, 83). The duration of treatment with ampicillin plus gentamicin positively correlated with the decrease in alpha diversity (58). One study found a lower alpha diversity in children treated for more than seven days with ampicillin plus gentamicin compared with these treated for a shorter duration (94). Another study found a lower richness in children treated with ampicillin plus gentamicin compared with controls two months after treatment (52) (Figure 2). In contrast, one study found no difference in alpha diversity at two weeks of life after treatment with ampicillin plus gentamicin (69), and another one, one year after treatment with penicillin plus tobramycin (26). Yet another study compared the alpha diversity one week after treatment with ampicillin plus tobramycin, ampicillin plus tobramycin plus metronidazole, and ampicillin plus cefotaxime and found no difference between these groups (85). Another study compared alpha and beta diversity after treatment with ampicillin plus gentamicin and ampicillin plus cefotaxime and found no differences at the latest follow up at 30 days of life (40).

### Composition

**Figure 4:**
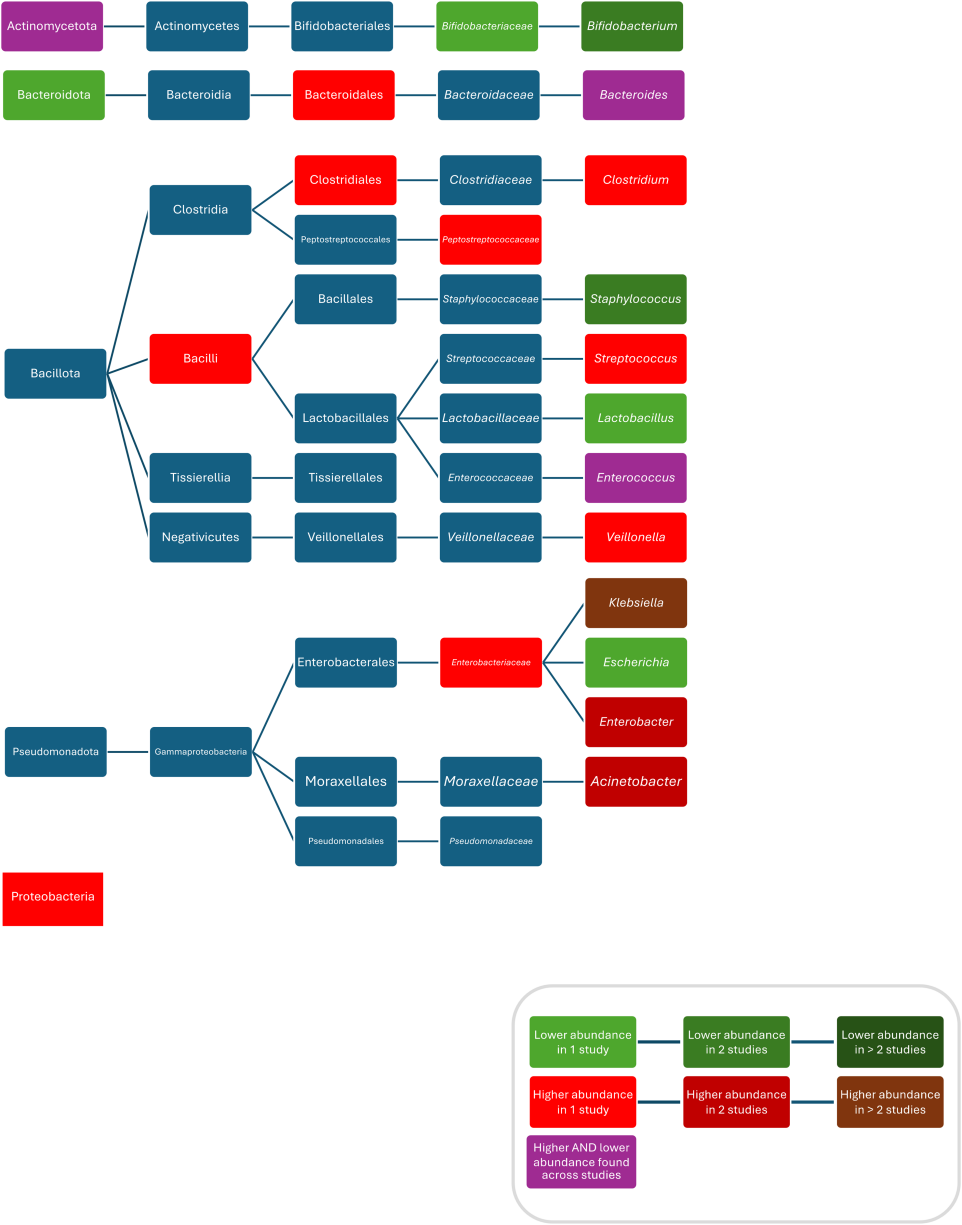
Differences in bacterial abundance or colonization rate between children treated with penicillins plus aminoglycosides and controls. Studies included investigated ampicillin plus gentamicin (52, 58, 69, 81, 93, 94), penicillin plus gentamicin (81, 83), and penicillin plus tobramycin (26). Studies which did not compare ABX group to controls and studies not providing *p*-values were excluded.

Compared to controls, after treatment with ampicillin plus gentamicin increased abundances/colonisation rates of Proteobacteria (52), *Bacilli* (94), *Clostridiales* (94), *Bacteroidales* (94), *Enterobacteriaceae* (52), *Peptostreptococcaceae* (52), *Clostridium* (52), *Klebsiella* (37, 83, 94), *Enterobacter* (58, 93), *Klebsiella/Enterobacter* (93), *Veillonella* (80), *Streptococcus* (80), and *Enterococcus faecalis* (37), and decreased abundances of *Bifidobacteriacea* (52), *Bifidobacterium* (52, 83), *Escherichia* (83), *Staphylococcus* (58, 83), and *Lactobacillus* (52); after treatment with penicillin plus gentamicin increased abundances of *Acinetobacter* (83), and *Klebsiella* (83), and decreased abundances of *Bifidobacterium* (83), *Escherichia* (83), *Staphylococcus* (83), and *Escherichia coli* (83); after treatment with penicillin plus netilmicin decreased abundance of *Clostridum difficile* (63); and after treatment with penicillin plus tobramycin decreased abundances of Bacteroidetes were found (26). Conflicting results were reported for the abundance of Actinobacteria (52, 69), *Enterococcus* (52, 58, 83) and *Bacteroides* (83, 94), and *Escherichia coli* (37, 93, 94) (Figure 4).

### ARGs

One study reported that changes in the ARG profile persisted for up to four months after treatment with amoxicillin/clavulanate plus gentamicin with 10 of 31 ARGs being more abundant, while after penicillin plus gentamicin five of 10 ARGs were found to be more abundant (83). One study found resistance to ampicillin in *E. coli, Klebsiella,* and *Enterobacter* after ampicillin plus gentamicin treatment (93).

## Penicillins plus cephalosporins

Five studies investigated combinations of penicillins plus cephalosporins, including 1,123 children (for detailed information see Supplementary Table S3) (34, 40, 83, 85, 101).

### Diversity

One study found a lower alpha diversity in children treated with amoxicillin plus cefotaxime compared with controls (83). The same study also found a high beta diversity with a dissimilar composition between children treated with amoxicillin plus cefotaxime and controls, which was still present four months after stopping ABX (83). One study compared alpha diversity one week after treatment with ampicillin plus tobramycin, ampicillin plus tobramycin plus metronidazole, and ampicillin plus cefotaxime and found no difference between these groups (85). Another study compared alpha and beta diversity after treatment with ampicillin plus gentamicin and ampicillin plus cefotaxime and found no differences at the latest follow-up at 30 days of life (40).

### Composition

Increased abundances of *Enterococcus* (101), *Clostridium* (101), and *Acinetobacter* (83), and decreased abundances of *Bifidobacterium* (101), *Akkermansia* (83), and *Escherichia coli* (83) were found in children after treatment amoxicillin plus cefotaxime or ceftazidime compared to controls. One study compared abundances after treatment with ampicillin plus tobramycin, ampicillin plus tobramycin plus metronidazole, and ampicillin plus cefotaxime and found no difference between groups (85). One study did not provide *p*-values for their analysis of colonisation rates (34).

### ARGs

One study reported changes in the ARG profile with 10 of 31 ARGs being more abundant after treatment with amoxicillin plus cefotaxime. Compared to penicillin plus gentamicin (five of 10 ARGs being more abundant), amoxicillin plus cefotaxime was found to have a higher impact on ARG abundance (83).

## Cephalosporins

The effect of cephalosporins on the intestinal microbiota in children was investigated in nine studies including 916 children (for detailed information see Supplementary Table S3) (55, 57, 60, 63, 87, 88, 90, 91, 93).

### Diversity

One study found a lower alpha diversity in infants treated with cefalexin compared with controls at two months of life (91). Another study found a lower richness immediately after treatment with cefotaxime and cefazoline compared to before ABX (55). One study found no difference in alpha diversity in neonates at ten days of life between the ABX group and controls after treatment with cefotaxime (57). Three studies did not analyse alpha diversity or richness (60, 87, 93).

### Composition

The administration of cefalexin was associated with increased abundances of *Enterobacteriaceae* (91), *Enterococcus* (91), and decreased abundances of *Bifidobacterium* (91) compared to controls. The administration of cefuroxime was associated with a decreased abundance of *Escherichia coli* (93). When comparing before and after treatment with cefaclor decreased bacterial counts of *Enterobacteriaceae* (87), *and Bifidobacterium* (87)*,* were found, while when comparing before and after treatment with ceftazidime decreased bacterial counts of *Enterobacteriaceae* (87), *Lactobacillus* (87)*,* and *Bifidobacterium* (87)*,* were found. After treatment with cefotaxime, compared to controls, increased abundances of *Enterobacteriaceae* (57) and *Parabacteroides* (57), and decreased abundances of *Bifidobacterium* (57), *Clostridium difficile* (63) and *Escherichia coli* (55) were found. After treatment with ceftriaxone, abundances of *Enterobacteriaceae* and *Lactobacillus* increased compared to before ABX (88). When comparing before and after treatment with cefpiramide decreased counts of *Enterobacteriaceae* (87), *Bacteroidaceae* (87), *Bifidobacterium* (87), *Lactobacillus* (87)*,* and *Staphylococcus* (87) were found.

### ARGs

After treatment with ceftriaxone, one study found *Klebsiella/Enterobacter, Citrobacter, Serratia* and *E. coli* to be resistant to cefoperazone and ceftriaxone and *Pseudomonas aeruginosa* to ceftriaxone (60). One study found increased abundances of multiple ARGs after treatment with cefotaxime, e.g., *β-lactamase (CMY-LAT-MOX), MFS efflux, ABC efflux,* and *robA* (55).

## Carbapenems

The effect of carbapenems on the intestinal microbiota was investigated in three studies including 67 children (for detailed information see Supplementary Table S3) (55, 96, 105).

### Diversity

One study found a reduced richness comparing before and two days after treatment with meropenem (55). Two studies did not analyse alpha or beta diversity (96, 105).

### Composition

The studies reported an increased bacterial count of *Enterococcus* (96), *Proteus* (96), *Pseudomonas* (96), *Enterobacter* (96), and *Staphylococcus epidermidis* (55), and a decreased abundance of *Klebsiella* (96), *Lactobacillus* (96), and *Streptococcus* (96) comparing before and after treatment with imipenem-cilastatin, while the second study did not find changes in the abundance of different bacteria (105).

### ARGs

The first study did not identify bacteria with resistance to imipenem (96), while in the second study, in one child *P. aeruginosa* resistant to imipenem was found after treatment. This child was previously also treated with aztreonam (105). One study found increased abundances of multiple ARGs after treatment with meropenem, e.g., *mecA, norA, dfrC, gyrA,* and *qacA* (55).

## Macrolides

The effect of macrolides on the intestinal microbiota was investigated in 13 studies including 802 children (for detailed information see Supplementary Table S3) (38, 46, 62, 70, 71, 75, 79, 80, 87, 95, 103, 111, 112).

### Diversity

Four studies found a lower alpha diversity (46, 79, 95, 103) and three a lower richness (70, 75, 80) between five days and 12–24 months after treatment with macrolides. One study reported a decrease in alpha diversity after 14 days but no further changes between 13 and 39 months after treatment with azithromycin (95). After treatment with azithromycin, one study found a difference in richness between children with ABX and controls, but no difference in alpha diversity (80) (Figure 2). Three studies analysed beta diversity and found a high beta diversity between ABX group and controls up to 14 days after treatment with azithromycin (80, 95) and a distinct composition on phylum and genus level in the ABX group up until six months after treatment with either azithromycin or clarithromycin without reporting results separately (70). One study did not detect a difference in beta diversity five days after treatment with azithromycin (46).

### Composition

**Figure 5:**
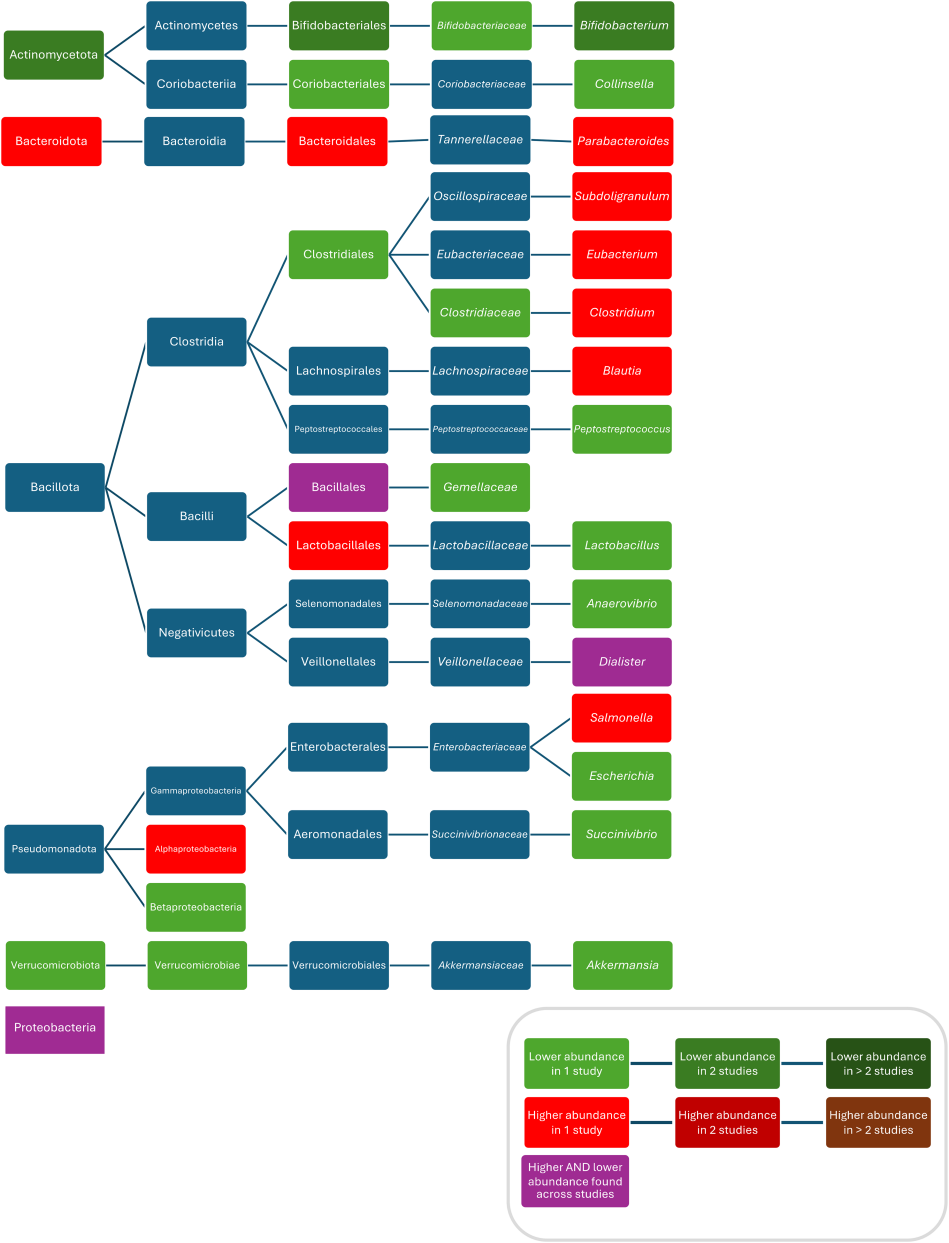
Differences in bacterial abundance or total bacterial count between children treated with macrolides and controls. Included studies investigated azithromycin (80, 95, 103), azithromycin and clarithromycin (70) or did not separately analyse different macrolides (71). Studies which did not compare ABX group to controls and studies not providing *p*-values were excluded.

Compared to controls, after treatment with azithromycin increased abundances/bacterial counts of *Clostridium* (95), and *Blautia* (46), and decreased abundances/bacterial counts of Actinobacteria (95), Verrucomicrobia (80), *Betaproteobacteria* (80), *Verrucomicrobiae* (80), *Bifidobacteriales* (95), *Bifidobacteriaceae* (95), *Clostridiacea* (95), *Bifidobacterium* (95), *Anaerovibrio* (46), *Peptoniphilus* (46), *Succinivibrio* (46), *Megasphaera* (46), *Escherichia* (80), *Akkermansia* (80), *Peptostreptococcus* (80), *Campylobacter hominis* (62), *Campylobacter ureolyticus* (62)*,* and *Campylobacter jejuni* (62) were found. Two studies did not report separate results for changes in abundances after treatment with different macrolides and found compared to controls, increased abundances of *Bacteroidetes* (70), *Alphaproteobacteria* (71), *Bacteroidales* (70), *Lactobacillales* (70), *Rikenellaceae* (70), *Subdoligranulum* (71), *Salmonella* (71), *Eggerthella* (70), *Bacteroides* (70), *Parabacteroides* (70), *Eubacterium* (70), and *Clostridium* (70), and decreased abundances of Actinobacteria (70), *Bifidobacteriales* (70), *Coriobacteriales* (70), *Clostridiales* (70), *Gemellaceae* (70), *Collinsella* (70), and *Bifidobacterium* (70, 71). Conflicting results were reported for abundances of Proteobacteria (70, 80), *Bacillales/Gemellales* (70, 71), and *Dialister* (70, 95) (Figure 3). Two studies found no difference in bacterial abundance between children with ABX and controls (38, 75). Three studies did report *p*-values for abundances (38, 79, 111). One study did not investigate bacterial abundances (103) (Figure 5).

### ARGs

ARGs were investigated in two studies (70, 114). One study found increased macrolide resistance after treatment with azithromycin or clarithromycin (without separate analysis), which declined linearly until going back to baseline 6 to 12 months after ABX. The study also found that the abundance of *ermF* and *ermB* genes correlated negatively with time since the last macrolide treatment, while the abundance of the *bsh* gene correlated positively with time since the last macrolide treatment (70). One study found a higher abundance of ARGs and plasmids in the ABX group compared with controls, without performing a separate analysis for different macrolides (75).

## Trimethoprim/sulfamethoxazole

The effect of trimethoprim/sulfamethoxazole on the intestinal microbiota was investigated in six studies, including 254 children (27, 36, 47, 53, 55, 79).

### Diversity

Two studies detected no difference in alpha diversity between the ABX group and the control group within the first week after ABX and after seven and eight years of continuous prophylactic treatment (Figure 2) (36, 79). Another study found a longitudinal increase in alpha diversity between six weeks and six months after the ABX in the ABX group (47). Another study found a decrease in diversity two weeks after the start of prophylactic treatment compared to before treatment, which recovered within one to two months (27). Another study found a decreased richness comparing before and two days after ABX (55). One study found no difference in bacterial counts between the two groups (53). Two studies analysed beta diversity, of which one did not detect a difference in between the ABX group and controls (36) and the other one found a lower beta diversity in the ABX group compared to controls (47). Comparing before to after treatment with trimethoprim/sulfamethoxazole decreased bacterial counts of *Enterobacter* (53) and *Veillonella* (53) were found.

### Composition

Comparing the ABX group to controls increased abundances of Enterobacteriales (27), *Alistipes onderdonkii* (36), *Eggerthella lenta* (36), *Clostridium bartlettii* (36). *Haemophilus parainfluenzae* (36), *Streptococcus mutans* (36), *Streptococcus parasanguinis* (36)*,* and *Streptococcus vestibularis* (36)*,* and decreased abundances of *Enterobacteriaceae* (36) were found after ABX. One study detected no differences in bacterial abundance after treatment with trimethoprim/sulfamethoxazole (47) and another study did not provide *p*-values for changes in abundances (79).

### ARGs

ARGs were studied in one study, *dfr* and *sul* genes were found to be more abundant in the ABX group after the ABX compared to before (47).

## Aminoglycosides

The effect of aminoglycosides on the intestinal microbiota was investigated in three studies, including 52 children (for detailed information see Supplementary Table S3) (55, 87, 112).

### Diversity

The first study found no difference in alpha diversity three days after treatment with gentamicin (55) and the second did not analyse alpha diversity (87). Both studies did not analyse beta diversity.

### Composition

One study observed decreased bacterial counts of *Enterobacteriaceae* (87), *Streptococcus* (87), *Clostridium* (87), and *Lactobacillus* (87) comparing before and three to six days after treatment with gentamicin.

### ARGs

One study found increased abundances of multiple ARGs after treatment with gentamicin, e.g., *evgA* and *emrK* (55).

## Glycopeptides

The effect of glycopeptides on the intestinal microbiota was investigated in three studies, including 48 children (for detailed information see Supplementary Table S3) (45, 55, 84).

### Diversity

One study found a lower alpha diversity seven days after treatment with vancomycin compared to before ABX, but no difference between the ABX group and controls (45). Another study found a lower richness two days after treatment with vancomycin compared to before ABX (55). One study found no difference in alpha diversity after treatment with vancomycin at 25 days of life (84).

### Composition

One study found increased abundances of *Staphylococcus* and decreased abundances of *Commamonadaceae, Pseudomonas, Bifidobacterium* when comparing before and after ABX (45). Another study reported increased abundances of *Staphylococcus* (in intestinal tissue but not in stool) when comparing children treated with vancomycin to other ABX (84).

### ARGs

One study found an increase in ARGs after treatment with vancomycin, e.g., *evgA, emrK* (55).

## Mixed and not specified ABX

Various ABX without reporting separate results were investigated in 19 studies (24, 31–33, 44, 48, 49, 54, 59, 64–66, 72–74, 77, 86, 92, 97), while another 20 did not specify which ABX were investigated (28–30, 38, 42, 43, 50, 51, 56, 61, 67, 68, 76, 82, 98, 99, 104, 106, 109, 115). These studies included a total of 5,139 children (for detailed information see Supplementary Table S3).

### Diversity

A decreased alpha diversity in ABX compared to controls was found in seven studies (29, 54, 72, 74, 77, 97, 98) and lower richness in two studies (66, 99). One study found a decrease in alpha diversity during ABX and an increase afterwards (64). One study found a lower alpha diversity in children treated for two days compared to seven days or more (44). Two studies reported that alpha diversity inversely correlated with increase in number of ABX courses (54, 74). One study found a decrease in diversity and in richness with each additional day of ABX (76) and another study found a lower diversity within the Bacteroidetes phylum in the ABX group than in controls (48). Other studies found no difference in alpha diversity between birth and hospital discharge (86), at ten days of life (43), or up to six month of life, when comparing children after ABX to controls (92). One study found no difference in alpha diversity comparing before, during, and after ABX (25). Twelve studies did not analyse alpha diversity or richness (28, 31–33, 39, 42, 49, 56, 61, 67, 73, 82, 104). Five studies found a high beta diversity with a dissimilar bacterial composition between the ABX group and controls (54, 72, 92, 98, 25). One study found a dissimilar composition in children with two days of ABX compared to seven or more days (44). One study found a dissimilar composition between neonates receiving ABX in the first week of life only and neonates receiving ABX in the first week of life plus after the first week of life (54). Samples collected in controls showed more similarity to each other than samples from the period when children were starting or stopping ABX (30). One study found no effect of ABX on beta diversity at one year of life (51). Another study found a decrease in beta diversity in neonates from week one to week three of life in the ABX group compared to controls (97).

### Composition

**Figure 6:**
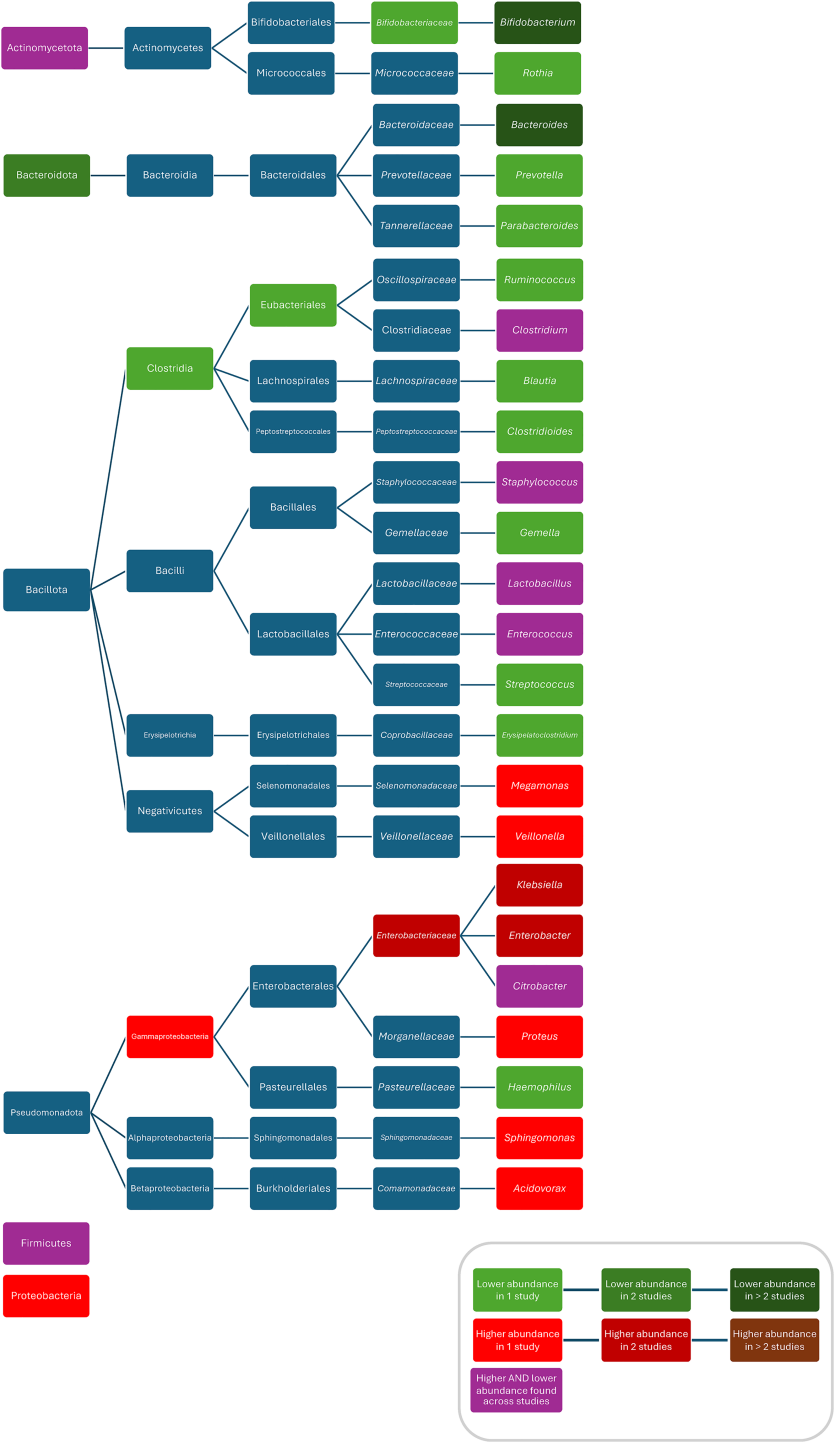
Differences in bacterial abundance or colonisation rate between ABX groups and controls of studies investigating various ABX without separate analysis of individual ABX. Included studies investigated: ampicillin, nafcillin, gentamicin, tobramycin, others (97), penicillin (plus aminoglycoside), others (92), ampicillin/sulbactam, cefotaxime (72), penicillin plus gentamicin, amoxicillin plus gentamicin, amoxicillin plus ceftazidime, others (48), ampicillin and cefotaxime, amikacin, vancomycin, others (65), ampicillin, cefotaxime, gentamicin, others (54), cephalosporin, penicillin, others (113), penicillins, macrolides, cephalosporins, others (115), beta-lactams, aminoglycosides, vancomycin, others (73), benzylpenicillin, cloxacillin, flucloxacillin, others (32), benzylpenicillin, cloxacillin, flucloxacillin, others (31), penicillin, penicillin plus gentamicin, others (33), or did not specify which ABX they investigated (28, 29, 42, 56, 61, 82, 98). Studies which did not compare ABX group to controls and studies not providing p-values were excluded.

When comparing ABX groups and controls, after ABX, increased abundances/colonisation rates/bacterial counts for Proteobacteria (28), *Gammaproteobacteria* (73), *Enterobacteriaceae* (29, 54), *Veillonella* (54), *Klebsiella* (56), Escherichia/Shigella (72), *Enterobacter* (56), *Klebsiella/Enterobacter* (31), *Sphingomonas* (97), *Acidovorax* (97), *Proteus* (42), *Bacteroides vulgatus* (74), *Bifidobacterium bifidum* (74), *Staphylococcus epidermidis* (54), *Veillonella parvula* (54), *Veillonella unspecified* (54), *Klebsiella oxytoca* (54), *Escherichia coli* (54), *Bifidobacterium breve* (54)*,* and decreased abundances for Bacteroidetes (48, 72, 76, 92), *Clostridiales* (54), *Bifidobacteraceae* (54), *Prevotella* (54), *Bacteroides* (29, 65, 72, 77, 82), *Parabacteroides* (29), *Ruminococcus* (25), *Haemophilus* (97), *Blautia* (97), *Erysipelatoclostridium* (97), *Gemella* (97), *Rothia* (97), *Streptococcus* (97), *Clostridium perfringens* (33), *Eubacterium rectale* (98), *Akkermansia muciniphila* (80), *Lactobacillus mucosae* (80), *Bacteroides fragilis* (80), *Actinomyces odontolyticus* (74), *Bifidobacterium longum* (50, 54, 74), *Bifidobacterium bifidum* (50, 54) *Bifidobacterium lactis* (50), *Escherichia coli* (30, 31) *Escherichia coli 8711* (80), *and Escherichia coli 17709* (80) were found. Conflicting results were found for Actinobacteria (28, 72), Firmicutes (28, 72, 92), *Clostridium* (29, 65, 73, 76, 98), *Enterococcus* (54, 77, 97), *Lactobacillus* (32, 33, 61, 65, 92), *Bifidobacterium* (29, 32, 54, 72, 82), *Citrobacter* (42, 56), *Staphylococcus* (54, 97), *Clostridium difficile* (33, 67), *Clostridium butyricum* (33, 49), *Enterococcus faecialis* (31, 54)*,* and *Bifidobacterium breve* (50, 54, 74) (Figure 6). One study found *Clostridium perfringens* abundance decreased compared to controls 3–4 weeks after ABX and increased 5–6 weeks after ABX (42). One study found a lower abundance of Bacteroides and a higher abundance of Actinobacteria, Proteobacteria, *Bacteroidetes* in children treated for more than seven days compared to these treated for two days (44). Another study found lower abundances of *Lactobacillus* and *Enterococcus* after more than seven days of treatment compared to less than seven days of treatment (68). Three studies did not compare bacterial abundances (66, 99, 104). One study, only investigating *Bifidobacterium* abundance, found no difference in abundance between the ABX group and controls (39).

### ARGs

ARGs were studied in two studies, which found a higher abundance of ARGs in ABX group including resistance to ABX rarely/never used in neonates and to multidrug-resistant organisms (MDROs) (54), higher abundance of ARGs and episomally encoded genes in the ABX group (98).

## Discussion

The first three years of life are pivotal for the development of the intestinal microbiota (14, 25), which, in turn, plays a crucial role in shaping the immune system. Moreover, early colonization of the intestine significantly impacts later microbial communities, so changes in this early period will have long-lasting consequences (115). A lower diversity of the intestinal microbiota has been associated with an increased risk of developing allergic diseases, type 1 diabetes, and rheumatic diseases (116–118). It is therefore concerning that all the ABX investigated in the studies in our review had profound effects on the intestinal microbiota in children associated with a decrease in alpha diversity. In some studies, this decrease was positively associated with duration of ABX. This effect was prolonged, persisting up to 12–24 months after stopping ABX for macrolides, and up to 36 months for ABX in the neonatal period.

In our review, we found that exposure to certain ABX (penicillins, penicillins plus gentamicin, cephalosporins, carbapenems, macrolides, and aminoglycosides, but not trimethoprim/sulfamethoxazole) is associated with decreased abundances of Actinobacteria (52, 70, 95), *Bifidobacteriales* (70, 95), *Bifidobacteriaceae* (52, 54, 95), and/or *Bifidobacterium* (52, 57, 70, 83, 87), and *Lactobacillus* (52, 70, 87, 88, 96). The direction of change in the abundance of *Enterobacteriaceae* depends on the ABX class but often an increase in *Enterobacteriaceae* other than *E. coli* is observed. These findings are in accordance with findings from a similar review in adults, which, after ABX, along with a decrease in alpha diversity, also found decreased abundances of *Bifidobacterium* and *Lactobacillus* and increased abundances of *Enterobacteriaceae,* other than *E. coli* (e.g., *Klebsiella, Citrobacter* and *Enterobacter*) (16). The results from our review, also align with results from a large *in vitro* study, which tested the effect of 144 ABX on 38 common human intestinal microbiota species (119). The study found that macrolides strongly inhibit the growth of most tested intestinal microbes, while beta-lactams have strain specific effects. This strain-specific effect of ABX might lead to large community composition disturbances with “killing-sensitive” strains more readily being eliminated from communities.

As mentioned above, almost all ABX are associated with a reduction in bacteria which have been identified as being beneficial. *Bifidobacterium* and *Lactobacillus* contribute to maintaining the gut barrier by producing high concentrations of short chain fatty acids (SCFA) such as acetate, propionate, and butyrate (120). However, SCFA not only serve as energy sources for the interstitial epithelium, but also have diverse effects on host physiology and immunity. In children, a decreased abundance of Actinobacteria has been associated with type 1 diabetes (121), while lower abundances of *Lactobacillaceae* and *Bifidobacteriaceae* have been associated with the development of allergic sensitization, eczema, or asthma (116). Reduced *Bifidobacterium* abundance has also been linked to childhood obesity (122, 123). Furthermore, it has been shown that peptidoglycans from *Bifidobacterium* can cross the blood-brain barrier and enhance neuronal maturation by influencing cytokine production by microglia (124). However, it's important to note that most of the studies investigating associations between variations in the intestinal microbiota composition and diseases are cross-sectional and therefore, it cannot be determined whether factors like ABX use, which may transiently decrease the abundance of different beneficial taxa, impact the development of these diseases. Nevertheless, a large meta-analysis has shown that ABX exposure in the first years of life is associated with an increased risk of developing atopic dermatitis, allergies, wheezing and asthma, obesity, rheumatological and neurodevelopmental diseases (12) and it is very likely that the mechanism behind this are changes in the microbiota (125).

The exact mechanism by which different intestinal bacteria influence the developing immune system is not yet clear but dysbiosis has been associated with a pro-inflammatory state (e.g., increased T-helper 17 cells (126). A decreased abundance of *Bacteroides uniformis* has been associated with increased production of interleukin (IL)-17, which is associated with increased neutrophil extracellular trap (NET) formation (114). These web-like structures are composed of DNA, histones, and antimicrobial proteins and released by activated neutrophils in response to infection or inflammation. NETs play an important role in fighting pathogens. In critically ill patients, progressive intestinal dysbiosis characterised by a high abundance of *Enterobacteriaceae* has been associated with a shift towards immature neutrophil populations with reduced NET formation (127). In contrast, dysregulated NET formation has been associated with various inflammatory and autoimmune diseases, e.g., rheumatological diseases (128). Other studies showed that the abundance of *Klebsiella pneumoniae* and *Streptococcus mitis* in the intestine positively correlates with the amount of natural killer cells in blood, the abundance of *B. uniformis* with immunoglobulin M levels as well as the erythrocyte sedimentation rate, and the abundance *Eubacterium eligens* with IL-4 and CD3 ^+^ CD8^+^ T cells levels (129).

Antimicrobial resistance is an increasing problem; it is estimated that by the year 2050, 10 million people will die annually because of infections with ABX-resistant bacteria (130). Therefore, another important finding in our review is the increase in ARG following ABX with all ABX classes, which persisted for as short as three weeks for some ABX, but up to four months after treatment for others. This is particularly important for ABX, such as amoxicillin and trimethoprim/sulfamethoxazole, which are often given for long-term prophylaxis. Even a transient increase in ARGs can have significant clinical implications, especially if a child develops a new infection during this period, as it may lead to infections that are harder to treat, requiring ABX with more side effects or which have broader spectrum activity, further exacerbating the problem of antimicrobial resistance.

The impact of ABX on the intestinal microbiota is multifaceted, influenced by both their spectrum of activity, and whether ABX are administered orally or intravenously. Formulation is also a factor: for example bacampicillin syrup alters bacterial abundance but tablets do not (131). ABX with a broad spectrum of activity and selective killing are the most dangerous to the intestinal microbiota and it is important to find drugs with a narrow spectrum of activity that inhibit pathogens but not intestinal commensals.

Other factors can mitigate ABX-induced dysbiosis. In infants, this includes breastfeeding and administration of pre-, pro- and postbiotics (132). Breastfeeding has been associated with an increased abundance of *Bifidobacterium, Staphylococcus,* and *Streptococcus* and lower abundances of *Enterococcus* and *Enterobacteriaceae* (133). Breast milk itself is an important source of pre-, pro- and postbiotics. A recent study showed that breastfeeding reduced the decrease in *B. infantis* which was seen after ABX and protected from ABX-associated increased asthma risk (134). The European Society for Paediatric Gastroenterology Hepatology and Nutrition recommends probiotics for the prevention of ABX-associated diarrhoea (132, 135), particularly *Saccharomyces boulardii* and *Lactobacillus rhamnosus* GG (136). In an RCT involving healthy children, the administration of *Bacillus subtilis* DE111 increased alpha diversity (137). Similarly, an observational study in preterm neonates showed an increase in alpha diversity, higher abundances of *Bifidobacterium* and *Lactobacillus*, and lower abundances of *Streptococcus* after the administration of a probiotic containing *Lactobacillus acidophilus* and *Bifidobacterium bifidum* (138). These findings, which contrast with the ABX-induced microbial disturbances observed in our systematic review, suggest that probiotics might help restore gut microbiota balance after ABX. A Cochrane review including 33 RCTs concluded that the administration of probiotics led to a reduction of ABX-associated diarrhoea from 19 to 8% with a number needed to treat of nine (132). However, many important questions remain open, such as the ideal timing, dosage, duration and strain selection for probiotics, as well as the benefit of co-administration with pre- and postbiotics.

### Strengths and limitations

A strength of this study is the comprehensive literature search, including children of all age groups and various ABX classes. However, the study is also subject to some limitations: First, most of the included studies were observational studies and only few RCTs were identified. Second, although most studies used longitudinal designs, the variability in follow-up periods, ranging from days to years, complicates inter-study comparisons. The timing of stool sampling is crucial, as changes in the bacterial composition of the intestinal microbiota can be transient or fluctuant. Third, a significant portion of the studies did not differentiate between various ABX or did not specify ABX. This may introduce bias into the results, as different ABX classes have been shown to have different effects. Therefore, the results of these studies have been pooled for this review. Even within an ABX class, the spectrum of activity differs and the effect on the intestinal microbiota will therefore be different. Fourth, except for five studies, all included participants had (suspected) infections, potentially introducing infections as a confounding factor. Furthermore, the analysis encompassed diverse age groups, though the impact of ABX on the microbiota is likely most pronounced in younger children, particularly neonates or infants. Lastly, microbiota research is largely influenced by the used analysis techniques. Molecular diagnostics are influenced by DNA extraction and library preparation method, used sequencing platforms and protocols and bioinformatic pipelines and tools. Culture-based diagnostics are influenced by the choice of culture media and incubation conditions and techniques for assessing ABX resistance.

In summary, ABX have profound effects on the intestinal microbiota, with notable differences between ABX classes. The duration of ABX likely influences the magnitude of these changes. Among those studied, macrolides have the most substantial impact while trimethoprim/sulfamethoxazole has the least pronounced effect. Important remaining questions include how long ABX-induced changes in the composition of the intestinal microbiota persist and the long-term effect of transient changes on health outcomes, particularly if they are given beyond the critical period of microbiota development in the first two to three years of life. Additionally, it is crucial to investigate whether ABX-resistant strains persist in the absence of selective pressure from ABX. Another area for further research is the development of remedies which can selectively protect intestinal microbiomes.
