# Supplementary material for: The effect of antibiotics on the intestinal microbiota in children - a systematic review
Source: Front Allergy. 2024 Oct 7;5:1458688. doi: 10.3389/falgy.2024.1458688 (PMC11491438; doi:10.3389/falgy.2024.1458688)
Supplement: Supplementary file 1 [file Table1.docx]

| Supplementary table 1 Summary of the findings of studies which investigated the association between antibiotic treatment and the composition of the intestinal  microbiota in children | | | | | | | | |
| --- | --- | --- | --- | --- | --- | --- | --- | --- |
| Author    Publication year    Country  Study design (level of evidence) | **No. of children (ABX, controls)**  **No. of stools samples (ABX, controls)**  **Participant characteristics**  **% female**  **Median/mean age/GA (range/IQR)**  **Antibiotic**  **Dose, frequency, duration**  **Route of administration**  **Previous ABX**  **% Probiotics** | **Sample type, storage conditions**  **DNA extraction kit**  **Stool analysis technique**  **Sequenced region for 16S rRNA and primers or library preparation kit**  **Sequencing platform**  **Sequencing length, depth**  **Database for taxonomic identification**  **Additional analysis** | **Time of stool testing** | **Findings** | | | | **Strengths**  **Limitations** |
|  |  |  |  | **Alpha diversity**  (time point, measure: ABX group/controls)  **Beta diversity** (time point, measure) | **Higher abundance in children with ABX**  (p< 0.05; 10 most abundant taxa/taxa > 0.1 % in either group, LDA score >4, relative abundance ratio >1) | **Lower abundance in children with ABX**    (p< 0.05; 10 most abundant taxa/taxa > 0.1 % in either group, LDA score >4, relative abundance ratio <1) | **Resistome**  **Other findings** |  |
| Hutchinson *et al.* (64)  2023  UK  Single centre, prospective cohort study (2b) | 29 (-,-)  1193 (-,-)  Hospitalised preterm neonates receiving ABX (reason ns)  34%  Mean GA 29 w (ns)  **Various ABX** (e.g. flucloxacillin plus gentamicin, ceftazidime, vancomycin)  ns, ns, ns  intravenous  ns  ns | Stool, sterile container, 4°C, then -80°C within 24 h  DNeasy PowerSoil Kit (*Qiagen*)  16S rRNA gene sequencing  V4, v4.SA501-508, v4.SB501-508, v4.SA701-  712, v4.SB701-71  500, 1786 reads/sample  MiSeq (*Illumina*)  ns | Daily samples between birth and 37/40 w of corrected GA or 12 w of life (whichever was sooner) | • **Decrease in diversity during ABX** (mean -0.71 units/w)  • **Increase in diversity after ABX** (0.2 units/w) | **Relative abundance between birth and discharge** | | • ns  • - | • Longitudinal sampling  • Large sample size³  • Small cohort²  • No control group  • Dose of ABX ns  • Frequency of ABX ns  • Duration of ABX ns  • No separate analysis of different ABX  • No information on interval without ABX before inclusion  • Database for taxonomic identification ns |
|  |  |  |  |  | **Family**  *Enterobacteriaceae* (increase of 11%/w during ABX) | **Family**  *Enterobacteriaceae* (decrease of 5%/w after ABX) |  |  |
| Li *et al.* (75)  2023  Denmark  Cross-sectional analysis (of a single centre prospective cohort study) (3b) | 662 (311, 351)  662 (ns, ns)  Infants with ‘common infections’ receiving ABX, infants with ‘common infections’ not receiving ABX as controls  49%  Median 1 y (range 11 m-2 y)  ns | Stool, 10% glycerol broth, −80 °C  NucleoSpin 96 Soil DNA Isolation Kit optimised for epMotion *(Macherey-Nagel)*  Shotgun metagenomic sequencing  Kapa HyperPrep kit *(KAPA Biosystems)*  NovaSeq *(Illumina)*  150, 58.9 ± 4.5 x 10^6^ reads/sample  Genome Taxonomy Database (GTDB), MetaPhlAn2  CARD v3.0.7 (ARG)  Platon v5.3 (plasmids) | < 15 d or 15-30 d or 1-3 m or 3-6 m after start of ABX | • ns  • **High beta-diversity** (dissimilar composition between ABX group and controls (< 15 d, PERMANOVA ns)) | **Relative abundance all ABX groups vs. controls** | | • **ARGs abundance**  Higher in ABX group (until 1 m after ABX); most abundant against tetracyclines, cephalosporines, macrolides  • **Plasmid abundance**  Higher in ABX group (until 1 m after ABX) | • Large cohort¹  • Large sample size³  • No ABX previous 1 y  • Analysis on species level  • Cross-sectional design  • Dose of ABX ns  • Frequency of ABX ns  • Duration of ABX ns  • Route of administration of ABX ns  • No samples before ABX |
|  |  |  |  |  | **Species**  *Escherichia coli* | **Species**  *Faecalibacterium prausnitzii*  *Haemophilus parainfluenzae* |  |  |
|  | **Penicillin** (ns)  ns, ns, ns  ns  ABX: ns  Controls: no ABX previous 1 y |  |  | • **Lower richness** in ABX group (< 15 d, ns)  • ns | No difference | No difference |  |  |
|  | **Ampicillin** (ns)  ns, ns, ns  ns  ABX: ns  Controls: no ABX previous 1 y |  |  | • **Lower richness** in ABX group (< 15 d, ns)  ns | No difference | No difference |  |  |
|  | **Macrolides** (15)  ns, ns, ns  ns  ABX: ns  Controls: no ABX previous 1 y |  |  | • **Lower richness** in ABX group (< 15 d, ns)  ns | No difference | No difference |  |  |
| Schwartz *et al*. (89)  2023  Niger  Retrospective cohort analysis (previous a community-based randomised, double-blinded, placebo-controlled trial) (3b) | 161 (78, 88)  384 (202, 182)  Infants with severe acute malnutrition receiving ABX or placebo  ns  ns (range 6-59 m)  **Amoxicillin**  80 mg/kg/d, divided into 2 doses, 7 d  Oral  ABX/controls: no ABX previous 7 d  ns | Stool, sterile containers, 2-8 °C during transport, then -80° C  ns  Shotgun metagenomic sequencing  ns  HiSeq (*Illumina*)  ns, 5.1 x 10^6^ reads/per sample  MetaPhlAn3 | 0 d, during ABX, 3, 7, 11 w after ABX  2 y (35) after ABX | • **Higher richness in ABX group after ABX** (2 y; 61 species/37 species)  • **Lower diversity in ABX group during ABX** (during ABX, Shannon index ns)  • **Higher diversity in ABX group after ABX** (2 y, Shannon index ns)  • **Longitudinal decrease** in diversity in ABX group (before ABX, Shannon index 2.2; during ABX, Shannon index 1.55)  • **High beta-diversity** (dissimilar composition between ABX group and controls (**during ABX**, Bray Curtis index ns) and longitudinally in ABX group (0 w vs. during ABX, Bray Curtis index ns)  • **No difference** in composition between ABX group controls **after ABX** (2 y, Bray Curtis index ns)) | **Relative abundance all ABX groups vs. controls** (time point in w) | | • **ARGs abundance**  Increase in ABX group from a baseline of 4800 reads per kilobase per 10^6^-6044 at 1 w; returned to baseline after 3 w  • - | • Double-blinded  • Placebo-controlled  • Longitudinal sampling  • Long follow-up⁶  • Analysis on species level  • Sex distribution of cohort ns  • Short interval without ABX before inclusion  • DNA extraction kit ns  • Library preparation kit ns  • Sequencing length ns |
|  |  |  |  |  | **Genus**  *Escherichia* (during ABX)  **Species**  *Weissella confusa* (104)  *Prevotella sp. 885* (104)  *Prevotella stercorea* (104)  *Holdemanella biformis* (104)  *Lactobacillus animalis* (104)  *Fusicatenibacter saccharivorans* (104)  *Catenibacterium mitsuokai* (104)  *Slackia isoflavoniconvertens* (104)  *Weissella cibaria* (104)  *Streptococcus macedonicus* (104)  *Gemmiger formicilis* (104)  *Actinomyces odontolyticus* (104) | **Genus**  *Klebsiella* (during ABX)  *Holdemanella* (during ABX)  *Dorea* (during ABX)  *Lactobacillus* (during ABX)  *Streptococcus* (during ABX)  **Species**  *Bifidobacterium bifidum* (104)  *Bifidobacterium longum* (104) |  |  |
| Barnett *et al.* (29)  2023  Netherlands  Cross-sectional analysis (of a multi-centre, prospective cohort study) (3b) | 1023 (23, 1000)  ns (ns, ns)  Non-hospitalised infants receiving ABX (reason ns), non-hospitalised infants not receiving ABX as controls  ns  Mean 32 d (SD ± 4 d)  **ns**  ns, ns, ns  ns  15% maternal ABX during pregnancy, 2% ABX via breast milk  ns | Stool, sterile tube, mailing of the sample the same day, then -80°C  Repeated bead beating and column-based purification  16S rRNA gene sequencing  V4, 515 F, 806 R  ns, ns  HiSeq (Illumina)  SILVA | 1 m of life | • **ABX associated with lower diversity** at 1 m (ns)  • ns | **Relative abundance ABX vs. controls** | | • ns  • Neither infant exposure to oral anti-fungals nor maternal exposure to antibiotics during pregnancy were associated with infant microbiota composition | • Large cohort¹  • Analysis on species level  • Cross-sectional design  • Sample number ns  • Sex distribution ns  • Reason for ABX ns  • ABX ns  • Dose of ABX ns  • Frequency of ABX ns  • Duration of ABX ns  • Route of administration of ABX ns  • Sequencing depth ns  • Sequencing length ns |
|  |  |  |  |  | **Family**  *Enterobacteriaceae*  **Genus**  *Clostridium sensu stricto 1* | **Genus**  *Bacteroides*  *Parabacteroides*  *Bifidobacterium*  **Species**  *Bacteroides caccae Bacteroides uniformis Bacteroides dorei*  *Parabacteroides distasonis*  *Parabacteroides merdae*  *Bifidobacterium longum*  *Bifidobacterium adolescentis* |  |  |
| Reyman *et al.* (83)  2022  Netherlands  Multi-centre,  non-blinded, non-placebo controlled, randomised controlled trial (1b) | 227 (147, 80)  1090 (147,943)  Hospitalised neonates with suspected early-onset sepsis randomised to different ABX, healthy neonates as controls  48%  ns (GA ≥36 w)  ns | Rectal swabs and/or stool, FaecalSwabTM kits, sterile faecal containers, immediately −20 °C, then −80 °C  Mag Mini DNA Isolation Kit  16S rRNA gene sequencing  V4, F515/R806  MiSeq (*llumina*)  150, ns  SILVA  Shotgun metagenomic sequencing (validation), Truseq Nano gel free library preparation kit  NovaSeq (*llumina*)  150, mean 1.38x10^6^ reads/sample  MetaPhlAn2, MEGARes  Targeted qPCR (ARGs) | Before ABX (median 1 d of life), immediately after ABX, 1, 4, 12 m of life | • **Lower in ABX group after ABX**  • Lowest immediately after ABX  • **Gradual recovery** until 1 y (after ABX, Shannon index 0.59/1.21)  • **High beta-diversity**  (dissimilar composition between ABX group and controls (1 m, PERMANOVA R² 3.0%; 4 m, PERMANOVA R² 1.4%; 1 y, PERMANOVA R² 1.1%)) | **Relative abundance all ABX groups vs. controls** (time point in d) | | • **ARG abundance**  Higher in ABX groups at 1 m of life (mean number of ARGs 9/31 vs. 7.5/31), no difference at later time points  • **ARGs with increased abundance in ABX groups**  Aminoglycosides  *aac(6')_aph(2''), aac(6')_Ib,*  *aac(6')_Ii aph(3')- III*  Beta-lactamases  *blaCMY-2, blaTEM*  Penicillin-binding protein *mecA*  Macrolides *ermB, ermbC*  Colistin *mcr-1;*  • **Breastfeeding**  Breastfeeding did not compensate for decreased *Bifidobacterium* abundance in ABX group | • Longitudinal sampling  • Large sample size³  • Long follow up⁶  • No blinding  • Dose of ABX ns  • Frequency of ABX ns  • No information on interval without ABX before inclusion for ABX group  • Sequencing depth ns |
|  |  |  |  |  | **Genus**  *Klebsiella* (1-122)  *Enterococcus* (1-121) | **Genus**  *Bifidobacterium* (1-36)  *Escherichia* (1-181)  *Staphylococcus* (1-229)  *Bacteroides* (1-255) |  |  |
|  | **Penicillin plus gentamicin** (49)  ns, ns, 48 h (mean)  Intravenous  ABX: ns  Controls: no ABX in first w of life |  |  | • **Lower diversity** in ABX group (ns, Shannon index ns/ns)  • **High beta-diversity**  (dissimilar composition between ABX group and controls (immediately after ABX, PERMANOVA R² 10.1%)) | Changes in abundance additional to the above | | • Lowest effect on ARGs  • 5 ARGs with increased abundance |  |
|  |  |  |  |  | **Genus**  *Acinetobacter*  *Klebsiella* | **Species**  *Escherichia coli* |  |  |
|  | **Amoxicillin/clavulanic acid plus gentamicin** (49)  ns, ns, 48 h (mean)  Intravenous  ABX: ns  Controls: no ABX in first w of life |  |  | • **Lower diversity** in ABX group  • Lowest among all ABX groups (ns, Shannon index ns/ns)  • **High beta-diversity**  (dissimilar composition between ABX group and controls (immediately after ABX, PERMANOVA R² 11.8%)) | **Genus**  *Klebsiella* | No difference | • Most persistent effects on ARGs (until 4 m)  • 10 ARGs with increased abundance |  |
|  | **Amoxicillin plus cefotaxime** (49)  ns, ns, 48 h (mean)  Intravenous  ABX: ns  Controls: no ABX in first w of life |  |  | • **Lower diversity** in ABX group (ns, Shannon index ns/ns)  • **Highest beta-diversity**  (most dissimilar composition between ABX group and controls, still observed at 4 m (immediately after ABX, PERMANOVA R² 14.7%; 4 m PERMANOVA R² 2.7%)) | **Genus**  *Acinetobacter* | **Genus**  *Akkermansia*  **Species**  *Escherichia coli* | • 10 ARGs with increased abundance |  |
| Kwon *et al*. (72)  2022  Korea  ns, cross-sectional study (3b) | 54 (20, 34)  54 (20, 34)  Hospitalised children with fever receiving ABX, healthy controls  35%  Mean 5 m (ns)  **Ampicillin/sulbactam** (ns)  150 mg/kg/d (ampicillin), ns, > 3 d  Intravenous  ABX group: ns  Controls: no previous ABX  **Cefotaxime** (ns)  150 mg/kg/d, ns, > 3 d  Intravenous  ABX group: ns  Controls: no previous ABX  ns | Stool, sterile container. -20°C, then -80°C  QIAamp Fast DNA Stool Mini Kit (Qiagen)  16S rRNA gene sequencing  V3-V4, F319 (5′- TCGTCGGCAGCGTCAGATGTGTATAAGAGACAGCCTACGGGNGGCWGCAG-3′), R806 (5′ -GTCTCGTGGGCTCGGAGATGTGTATAAGAGACAGGACTACHVGGG TATCTAATCC-3′)  ns, ns  MiSeq (*Illumina*)  Greengenes | 1 m after ABX | • **Lower diversity and richness** compared to controls (1 m, Shannon, Chao 1, observed OTUs ns)  • **High beta-diversity** (dissimilar composition between ABX group and controls (1 m, UniFrac distance ns)) | **Relative abundance ABX groups vs. controls** (abundance in % ABX group/controls) | | • ns  • **Metabolic pathways**  Higher gene expression in naphthalene degradation, glycolysis, gluconeogenesis, and lipoic acid metabolism in the ABX group compared to controls, lower gene expression in porphyrin and chlorophyll metabolism and fatty acid biosynthesis in ABX group compared to controls | • Healthy controls  • Cross-sectional design  • No separate analysis of different ABX  • No information on interval without ABX before inclusion for ABX group  • Sequencing depth ns  • Sequencing length ns |
|  |  |  |  |  | **Phylum**  Actinobacteria (22/14)  **Genus**  *Escherichia*  */Shigella* (30/16)  *Bifidobacterium* (21/13) | **Phylum**  Firmicutes (25/35)  Bacteroidetes (3/7)  **Genus**  *Bacteroides* (1/5) |  |  |
| Lebeaux *et al.* (74)  2022  Finland, USA  Multi-centre, prospective cohort study (2b) | 315 (ns, ns)¹  ns (ns, ns)  Non-hospitalised children from two cohorts (*cohort USA* n=282, *cohort Finland* n=33) receiving ABX (mostly for otitis media), non-hospitalised children from two cohorts not receiving ABX  39%  Mean 41 d at 1^st^ sample (SD 15 d)  **Various antibiotics** (e.g., amoxicillin, amoxicillin with clavulanate)  ns, ns, ns  ns  ns  ns | ***Cohort USA***  Stool, sterile container, home freezer, then -80°C  Zymo Fecal DNA extraction kit (*Zymo Research)*  Shotgun metagenomic sequencing  Nugen’s Ovation Ultralow V2 protocol  ns, ns  ns  MetaPhlAn3  ***Cohort Finland***  Stool, ns, -20 °C, shipping on dry ice, then -80 °C  QIAamp DNA Stool Mini Kit (*Qiagen*)  16S rRNA gene sequencing  V4, ns  HiSeq 2500 (*Illumina*)  ns, 48,131 reads/sample  Greengenes  Shotgun metagenomic sequencing  Nextera XT DNA Library Preparation Kit *(Illumina)*  HiSeq 2500 (*Illumina*)  101, 2.5 Gb/sample  MetaPhlAn 2.0  shortBRED (ARG) | Throughout the 1^st^ y of life (*cohort USA* mainly 6 w and 1 y of life, *cohort Finland* starting at 2 m) | • **Lower diversity** in ABX group compared to controls (1 y, Shannon ns)  • Bigger decrease in diversity in ABX group than in controls comparing the baseline sample to the 1 y sample  • Children exposed to 2 or more ABX between baseline and 1 y had a bigger decrease diversity  • The influence of age and antibiotic exposure within the first year explained 2.6% of the variation between samples | **Change in relative abundance ABX group between baseline and 1 y of life** (% change) | | • **ARG**  Bifidobacteria intrinsic *ileS* conferring resistance to mupirocin and *CfxA6* were increased in ABX group  • **Day care**  Among children attending day care, *Escherichia coli* and ARG abundance were positively associated with antibiotic use | • Longitudinal sampling  • Analysis on species level  • Overlapping cohort with Yassour et al. (n=33)  • Size of ABX group and control group ns  • Sample size ns  • Dose of ABX ns  • Frequency of ABX ns  • Duration of ABX ns  • Route of administration of ABX ns  • No information on interval without ABX before inclusion  • Sequencing depth ns (*cohort USA, cohort Finland)*  • Sequencing length ns (*cohort USA)*  • Sequencing platform ns (*cohort USA)* |
|  |  |  |  |  | **Species**  *Bacteroides vulgatus* (1.7)  Bifidobacterium bifidum (1.4) | **Species**  *Bacteroides fragilis* (1.6)  Actinomyces odontolyticus (0.008)  Bifidobacterium longum (1.6)  Bifidobacterium breve (1.1) |  |  |
| Xu *et al.* (97)  2022  USA  Multi-centre,  prospective cohort study (2b) | 68 (46, 22)  133 (ns, ns)  Hospitalised preterm neonates receiving ABX (reason ns), hospitalised preterm neonates not receiving ABX as controls  54%  Mean GA 31 w (range 24-35 w)  **Various ABX** (ampicillin nafcillin, gentamicin, tobramycin, vancomycin)  ns, ns, ns  ns  87% maternal ABX  ns | Stool, ns, -80°C  QIAamp PowerFecal Kit (*Qiagen*)  Shotgun metagenomic sequencing  Nextera XT DNA Library Preparation Kit  NextSeq 500 (*Illumina*)  159, 2 mio reads/sample  Kraken2 | 1, 3 w of life | • **Lower diversity** in ABX group than in controls (1 and 3 w, Shannon diversity ns)  • Decrease in beta-diversity from w 1 to w 3 in ABX group compared to controls (1 and 3 w, Bray Curtis ns) | **Relative abundance all ABX groups vs. controls** (time point in w of life) | | • ns  • **Metabolic pathways** Metabolic pathways required for short-chain fatty acid synthesis were increased in controls, but not in ABX group at 3 w of life | • Longitudinal sampling  • Reason for ABX ns  • Dose of ABX ns  • Frequency of ABX ns  • Duration of ABX ns  • Route of administration of ABX ns  • Storage medium ns |
|  |  |  |  |  | **Genus**  *Sphingomonas* (1)  *Acidovorax* | **Genus**  *Haemophilus* (1)  *Enterococcus* (1)  *Blautia* (1, 3)  *Erysipelatoclostridium* (3)  *Gemella* (3)  *Rothia* (3)  *Clostridiodes* (3)  *Clostridium* (3)  *Streptococcus* (1, 3)  *Staphylococcus* (1, 3) |  |  |
| Ainonen *et al.* (26)  2021  Finland  Single centre, cross-sectional analysis (3b) | 48 (21, 27)⁵  ns (ns, ns)  Hospitalised neonates receiving ABX (reason ns), hospitalised neonates not receiving ABX as controls  ns  ns  **Penicillin G plus tobramycin**  ns, ns, ns  Intravenous  ABX group: 66% had ABX between postnatal ABX and sampling, median 3 m before sampling  Controls: 40% had ABX between inclusion and sampling, median 2 m before sampling  100% (ABX group, *Lactobacillus reuteri*) | Stool, ns, -20°C  QIAamp Fast DNA stool mini kit (*Qiagen*)  16S rRNA gene sequenc6ing  V4-V5, R926, F519  Ion Torrent  400, ns  SILVA | 1 y of life | • **No difference** in diversity between ABX group and controls (1 y, Shannon 3.0/3.0)  • ns | **Relative abundance ABX group vs. controls** (time point in y of life) | | • ns  • **Intermittent ABX**  Additional ABX between inclusion and 1 y of life was not associated with changes in abundance or Shannon diversity at 1 y of life | • -  • Cross-sectional design  • Sample size ns  • Overlapping children with Tapiainen et al.⁵  • Reason for ABX ns  • Sex distribution of cohort ns  • Age distribution of cohort ns  • Dose of ABX ns  • Frequency of ABX ns  • Duration of ABX ns |
|  |  |  |  |  | **Species**  *Escherichia coli* (1) | **Phylum**  Bacteroidetes (1) |  |  |
| Bender *et al.* (30)  2021  USA  Single centre, prospective cohort study (2b) | 25 (7, 18)  51 (17, 34)  Hospitalised term and preterm neonates receiving ABX, hospitalised term and preterm neonates not receiving ABX as controls  40%  Mean GA 37 w (range 30-40 w)  **ns**  ns, ns, ns  ns  ABX group: 67% ABX at birth  Controls: 86% ABX at birth  ns | Stool, ns, -20°C, then -80°C  AllPrep DNA/RNA Mini Kit (Qiagen)  Shotgun metagenomic sequencing  Nextera XT DNA Library Preparation Kit (*Illumina*)  ns, mean 4,498,868 reads/sample  Nextseq (Illumina)  Kraken | Once a week during hospitalisation (duration ns) | • ns  • Longitudinal samples collected when subjects were not taking antibiotics showed more similarity to each other compared to samples collected during the period when subjects were starting or stopping antibiotics (Bray-Curtis 0.29 vs. 0.63) | **Relative abundance ABX vs. controls** (during ABX) | | • ns  • **Metabolic pathways**  Four microbial pathways were disrupted by antibiotics given at-birth (folate synthesis, glycerolipid metabolism, fatty acid biosynthesis, glycolysis) | • Longitudinal sampling  • Analysis on species level  • Small cohort²  • ABX ns  • Dose of ABX ns  • Frequency of ABX ns  • Duration of ABX ns  • Route of administration of ABX ns  • Storage medium ns  • Sequencing depth ns |
|  |  |  |  |  |  | **Species**  *Escherichia coli* |  |  |
| Chang *et al.* (40)  2021  Taiwan  Single centre, retropective cohort study (2b) | 24 (-,-)  67 (-,-)  Hospitalised preterm neonates receicing two different ABX for suspected bacterial infections  50%  Median GA 29 w (ns)  0% | Stool, ns, -80°C  QIAmp DNA stool mini kit (Qiagen)  16S rRNA gene sequencing  V3-V4, forward primer 5′ TCGTCGGCAGCGTCAGATGTGTATAAGA- GACAGCCTACGGGNGGCWGCAG, reverse Primer 5′ GTCTCGTGGGCTCGGAGATGTGTATAA- GAGACAGGACTACHVGGGTATCTAATCC  600, > 100’000 reads/sample  MiSeq (Illumina)  Greengenes | d 7, 14, 30 of life | • **No difference in diversity** between the AG and AC group  • Decrease in richness at 14 and 30 d compared to 7 d (Chao 1, 175/375, 185/375)  • **No difference** between AG and AC group | **Relative abundance AG group vs. AC group** (time point in d of life, abundance in % AG/AC) | | • ns  • - | • Longitudinal sampling  • No control group, only comparison of 2 ABX  • Dose of ABX ns  • Frequency of ABX ns  • Duration of ABX ns  • Route of administration of ABX ns  • Storage medium ns |
|  | **Ampicillin plus gentamicin** (AG, n=10)  ns, ns, 3 d  ns  80% maternal ABX |  |  |  |  | **Genus**  *Enterococcus* (7, 1/12) |  |  |
|  | **Ampicillin plus cefotaxime** (AC, n=14)  ns, ns, 7 d  ns  71% maternal ABX |  |  |  |  |  |  |  |
| Kim et al. (69)  2021  USA  Double-blind, placebo-controlled, randomised controlled trial (1b) | 22 (11,11)  ns (ns, ns)  Hospitalised preterm neonates receiving prophylactic ABX, hospitalised preterm neonates receiving placebo as controls  ns  Mean GA 32 w (range 28-34 w)  **Ampicillin plus gentamicin**  ns, 1x after birth, 48 h  Intravenous  ns  ns | Stool, ns, ns  ns  16S rRNA gene sequencing  ns, ns  ns, ns  ns  ns | 2 w of life | • **No difference**  • **No difference** | **Relative abundance all ABX group vs. controls** (time point in w of life) | | • ns  • - | • Double-blinded  • Placebo-controlled  • Small cohort²  • Sample number ns  • Sex distribution ns  • Dose of ABX ns  • No information on interval without ABX before inclusion for ABX group  • DNA extraction kit ns  • Sequenced region for 16S rRNA gene sequencing ns  • Primers ns  • Sequencing platform ns  • Sequencing depth ns  • Sequencing length ns  • Database for taxonomic identification  • Short follow-up⁷ |
|  |  |  |  |  | **Phylum**  Actinobacteria (2) |  |  |  |
| Gong *et al.* (57)  2021  China  ns, prospective cohort study (2b) | 20 (10, 10)  120 (ns, ns)  Hospitalised preterm neonates receiving ABX due to high risk of infection (e.g. premature rupture of membrane), hospitalised preterm neonates not receiving ABX as controls  40%  Mean GA 34 w (ns)  **Cefotaxime**  100 mg/kg/d, divided into 2 doses, 7 d  ns  ns  0% | Stool, sterile container, -80°C  Cetyl trimethyl ammonium bromide method  16S rRNA gene sequencing  V3-V4, 341F-806R, 5’-CCTAYGGGRBGCASCAG and 5’-GGACTACNNGGGTATCTAAT  ns, ns  IonS5TMXL (*ThermoFisher Scientific*)  SILVA | 1, 3, 10 d of life | • **No difference**  • ns | **Relative abundance ABX group vs. controls** (time point in d of life) | | • ns  • **Metabolic pathways**  Lower replication and repair function, nucleotide metabolism function, and purine metabolism pathway in ABX group compared to controls | • -  • Small cohort²  • Route of administration of ABX ns  • No information on interval without ABX before inclusion/maternal ABX  • Sequencing length ns  • Sequencing depth ns |
|  |  |  |  |  | **Familiy**  *Enterobacteriaceae* (10)  **Genus**  *Parabacteroides* (10) | **Genus**  *Bifidobacterium* (10) |  |  |
| Russell *et al*. (86)  2021  USA  ns, non-blinded, non-placebo-controlled, randomised trial/prospective cohort study (2b) | 91 (66, 25)  693 (ns, ns)  Hospitalised preterm neonates receiving ABX for various (suspected) infections or being randomised to ABX or placebo  51%  ns (GA range 23-32 w)  **Various ABX** (e.g. vancomycin, oxacillin, gentamicin)  ns, ns, ns  ns  ns  0% | Stool, ns -80°C  E.Z.N.A Stool Extraction Kit (*Omega Bio-tek*)  16S rRNA gene sequencing  V3-V4, 341F, 806R  600, 10,000 reads/sample  MiSeq (*Illumina*)  SILVA | Weekly during hospitalization (ns) | • **No difference**  • **No difference** | ns | ns | • ns  • - | • Large sample size³  • Dose of ABX ns  • Frequency of ABX ns  • Duration of ABX ns  • Route of administration of ABX ns  • No information on interval without ABX before inclusion |
| Akagawa et al. (27)  2020  Japan  Multi centre, prospective cohort study (2b) | 35 (23, 12)  90 (ns,ns)  Hospitalised children receiving prophylactic ABX for urinary tract infection with vesicoureteral reflux grade III or higher, hospitalised children urinary tract infection with vesicoureteral reflux grade II or lower not receiving prophylactic ABX for urinary tract infection as controls  51%  Median 5 m (IQR 4.4-7.2 m)  **Trimethoprim-sulfamethoxazole**  0.2 mg/d, 1x/d, prophylactic  Oral  ABX/controls: 100% had ceftriaxone and cefditoren pivoxil for 14 d before  ABX group: 73%  Controls: 77% | Stool, sterile container, -80°C  NucleoSpin! DNA Stool Kit  16S rRNA gene sequencing  V1-V5, ns  ns, ns  Ion PGM Sequencer  Ion Plus Fragment Library Kit, Ion Xpress Barcode Adapters Kit (*Thermo Scientific*) | Before and during ABX, 1-2, 3-4, 5-6 m after ABX | • **Decrease in diversity** 2 w after start of treatment compared to before (Shannon 2.9/1.4), recovered within 1-2 m | **Relative abundance ABX group vs. controls of all time points together** (% ABX/controls) | | • ns  • - | • Longitudinal sampling  • Small cohort²  • Sequencing depth ns  • Sequencing length ns  • 100% had previous ABX |
|  |  |  |  |  | **Order**  Enterobacteriales (20/13) |  |  |  |
| Lindberg *et al.* (76)  2020  USA  Single centre,  Prospective cohort study (2b) | 9 (-,-)  29 (-,-)  Hospitalised preterm neonates with NEC and without NEC receiving ABX  ns  Mean GA 25 w (range 23-27 w)  **ns**  ns, ns, 2-31 d  ns  ns | Stool, sterile container, -80°C  MoBio Power Soil kit (MoBio)  16S rRNA gene sequencing  V4, 515F, 806R  300, ns  MiSeq (Illumina)  Greengenes | Weekly until discharge (range 16-48 d of life) | • **Decrease in richness** **with each day of ABX** (decrease of OTU count of 0.5/day of ABX)  • **Decrease in diversity with each day of ABX** (decrease of Shannon of 0.01/day of ABX) | **Relative abundance relative to days of ABX** (decrease in % per day of ABX) | | • ns  • - | • Longitudinal sampling  • No control group  • Small cohort²  • Low sample number⁴  • Sex distribution of cohort ns  • ABX ns  • Dose of ABX ns  • Frequency of ABX ns  • Duration of ABX ns  • Route of administration of ABX ns  • No information on interval without ABX/maternal ABX before inclusion  • Sequencing depth ns |
|  |  |  |  |  |  | **Genus**  *Clostridium* (1) |  |  |
| Eck *et al.* (48)  2020  Netherlands    Multi-centre,  Prospective cohort study (2b) | 45 (21, 24)  ns (ns, ns)  Hospitalised neonates receiving ABX for suspected neonatal sepsis, hospitalised neonates not receiving ABX as controls  56%  Mean GA 40 w (ns)  **Various ABX** (penicillin plus gentamicin (n=11), amoxicillin plus gentamicin (n=8), amoxicillin plus ceftazidime (ns), amoxicillin/clavulanate plus gentamicin (ns))  ns, ns, 7 d  Intravenous  ABX group: 19% maternal ABX  Controls: 12% maternal ABX  ns | Stool, sterile container, -20°C  EasyMAG  16S-23S IS profiling  phylum-specific fluorescently-labeled PCR primers  GeneAmp PCR System 9700  IS-pro (IS-diagnostics) | 1 w, 1 m, 3 m of life | • **Lower diversity** **within the Bacteroidetes phylum** in the ABX group than in controls (1 w, 1 m, 3 m, Shannon ns)  • ns | **Colonisation rate ABX group vs. controls** (time point in w or m of life) | | • ns  • - | • Longitudinal sampling  • Small cohort²  • Sample size ns  • ABX ns  • Dose of ABX ns  • Frequency of ABX ns  • No separate analysis of different ABX |
|  |  |  |  |  |  | **Phylum**  Bacteroidetes (1 w, 1 m, 3 m) |  |  |
| Jia *et al.* (68)  2020  China  Single centre, prospective cohort study (2b) | 101 (51, 50)  558 (ns, ns)  Hospitalised preterm neonates receiving ABX (reason ns),  hospitalised term neonates not receiving ABX  40%  ABX group: mean GA 32 w  Controls: mean GA 39 w  **ns**  ns, ns, mean 29 d, < 7 (short-term (ST), ≥ 7d (long-term (LT))  ns  ns  ABX group: Mean probiotic use for 15 d  Controls: Mean probiotic use for 3 d | Stool, sterile tube, -20°C, then -80°C  QIAamp Fast DNA Stool Mini Kit *(Qiagen)*  16S rRNA gene sequencing  V3-V4, 338F, 806R  ns, ns  MiSeq (Illumina)  SILVA | Within 48 h after birth, 7, 14, 21, 42, 70, 90 d of life | • ns  • ns | **Relative abundance (LT vs. ST)** | | • ns  • - | • Large sample size³  • Longitudinal sampling  • Dose of ABX ns  • Frequency of ABX ns  • Route of administration of ABX ns  • No information on interval without ABX before inclusion  • Sequencing depth ns  • Sequencing length ns |
|  |  |  |  |  |  | **Genus**  *Lactobacillus Enterococcus* |  |  |
| Lu *et al.* (77)  2020  China  ns, prospective cohort study (2b) | 16 (6, 10)  48 (30, 18)  Hospitalised preterm neonates receiving ABX (reason ns), hospitalised preterm neonates not receiving ABX as controls  44%  Mean GA 35 w (SD ± 0.7 w)  **β-lactam ABX** (e.g. piperacillin-tazobactam, piperacillin-sulbactam, cefuroxime, cefoperazone-sulbactam sodium)  ns, ns, < 7d  ns  ns  0% | Stool, ns, -80°C  E.Z.N.A.® Stool DNA Kit (*Omega*)  16S rRNA gene sequencing  V4, 515F, 806R  ns, ns  NovaSeq (*Illumina*)  NCBI, RDP  LC–MS metabolomics | d 3, 7, 14 of life | • **Lower diversity** in ABX group compared to controls (d 7, Shannon ns)  • Lower diversity in ABX group on d 7 compared to d 14 (Shannon ns) | **Relative abundance ABX vs. controls** (time point in d of life) | | • ns  • **Metabolic pathways ABX group vs. controls**  Increase in some amino acids were in ABX group on d 3 of life (serine (18 fold), tyrosine (31 fold), and lysine (40 fold)), decrease in other amino acids (propionic acid (45 fold))  Bilde acids and carbohydrates (taurocholic acid (73 fold) were increased in ABX group on d 7 of life; citraconic acid (23 fold) increased in the ABX group on d 14, malonic acid (50 fold) and trehalose (11 fold) were decreased in ABX group on d 14 | • Longitudinal sampling  • Small cohort²  • Low sample number⁴  • Short follow-up⁷  • Reason for ABX ns  • ABX ns  • Dose of ABX ns  • Frequency of ABX ns  • Duration of ABX ns  • Route of administration of ABX ns  • No information on interval without ABX before inclusion for ABX group  • Storage medium ns  • No separate analysis of various ABX |
|  |  |  |  |  | **Genus**  *Enterococcus* (ns)  *Lactococcus* (3) | **Genus**  *Bacteroides* (3) |  |  |
| Rooney *et al.* (85)  2020  Canada  ns, retrospective cross-sectional study (3b) | 72 (-,-)  67 (-,-)  Term and preterm neonates receiving ABX for various reasons (e.g. suspected sepsis, abdmoninal infections)  49%  Mean GA 35 w  ns | Stool, sterile tube, -80°C  DNeasy PowerSoil Kit *(Qiagen)*  16S rRNA gene sequencing  V4, forward primer 5’-TCCTACGGGAGGCAGCAGT-3’, reverse primer 5’-GGACTACCAGGGTATCTAATCCTGTT-3’  150, 3736 reads/sample  MiSeq *(Illumina*)  Greengenes | Within 1 w after ABX | • **No difference** in diversity between treatment groups  • ns | **Relative abundance compared between ABX groups** (AT, AC, ATM) | | • ns  • Each additional day of ABX was associated with 16% lower obligate anaerobes and 18% lower butyrate- producers | • -  • Cross-sectional design  • Dose of ABX ns  • Frequency of ABX ns  • Route of administration of ABX ns  • No information on interval without ABX before inclusion  • Short follow-up⁷ |
|  | **Ampicillin plus tobramycin** (AT, n=52)  ns, ns, median 3 d (range 1-8 d)  ns  ns |  |  |  | No difference | No difference |  |  |
|  | **Ampicillin plus cefotaxime** (AC, n=8)  ns, ns, median 4 (range 2-8 d)  ns  ns |  |  |  | No difference | No difference |  |  |
|  | **Ampicillin plus tobramycin plus metronidazole** (ATM, n=12)  ns, ns, median 7 d (range 1-10 d)  ns  ns |  |  |  | No difference | No difference |  |  |
| Hinterwirth *et al.* (62)  2020  Burkina Faso  Community-based,  double-blinded, placebo-controlled randomised controlled trial (1b) | 62 (31, 31)¹  122 (62, 60)  Healthy children randomised to ABX or placebo  ns  ns (range 6-59 m)  **Azithromycin**  10 mg/kg/d, 1x/d on d 1, then 5 mg/kg/d, 1x/d for 4 d  Oral  ns  ns | Rectal swabs, stool nucleic acid collection and sterile tube with Norgen stool preservative *(Norgen, Ontario, Canada),* transport at ambient temperature, then −80 °C  Norgen stool DNA isolation kit (*Norgen*)  Shotgun metagenomic sequencing, NEBNext Ultra II DNA Library Prep Kit (*New England BioLabs)*  NovaSeq *(Illumina)*  150, ns  RDP, GreenGenes | 0 d (before ABX),  5 d after ABX | • ns  • ns | **Relative abundance ABX group vs. controls** (time point) | | • ns  • **Diarrhoea**  No difference in diarrhoea prevalence between ABX/Controls | • Longitudinal sampling  • Healthy children  • Blinded, placebo-controlled  • Analysis on species level  • Sex distribution of cohort ns  • No information on interval without ABX before inclusion  • Short follow-up⁷  • Sequencing length and depth ns |
|  |  |  |  |  | No difference | **Species**  *Campylobacter hominis* (5 d)  *Campylobacter ureolyticus* (5 d)  *Campylobacter jejuni* (5 d) |  |  |
| Zwittink *et al.* (102)  2020  Netherlands  Single centre, prospective cohort study (2b) | 63 (35 ,28)  263 (ns, ns)  Hospitalised term and preterm neonates receiving ABX for suspected early-onset sepsis  46%  ns (GA range 32-42 w)  **Amoxicillin plus ceftazidime**  100 mg/kg/day (each), divided into 2 doses, < 3 (short-term (ST), n=22), > 5 d (long-term (LT), n=13)  Intravenous  68% maternal ABX  ns | Stool, ns, -20°C, then -80°C  QIAamp Fast DNA Stool Mini Kit (*Qiagen)*  16S rRNA gene sequencing  V3-V4, ns  300, ns  MiSeq (Illumina)  SILVA | 1,2,3,4,6 w of life | • **No difference**  • ns | **Relative abundance ABX vs. controls** (time point in w of life) | | • ns  • - | • -  • Storage medium ns  • Sequencing depth ns |
|  |  |  |  |  | **Genus**  *Enterococcus* (LT only, 2)  *Lactobacillus* (1) | **Genus**  *Bifidobacterium* (1,2) *Streptococcus* (1)  *Escherichia-Shigella* (1) |  |  |
|  |  |  |  |  | **Relative abundance LT vs. ST group** (time point in w of life) | |  |  |
|  |  |  |  |  | **Genus**  *Clostridium* sensu stricto 1 (1)  *Enterococcus* (2) | **Genus**  *Veillonella* (1)  *Bifidobacterium (4,6)* |  |  |
| Korpela *et al.* (71)  2020  Netherlands  Multi-centre, prospective  cohort study (2b) | 40 (22,18)  163 (ns,ns)  RSV-infected infants receiving ABX (mostly for otitis media), RSV-infected infants not receiving ABX as controls  40%  Median 2.3 m (ns)  ns | Stool, container ns,  immediate freezing at -20 °C, then -70 °C  Repeated bead-beating method, RSC Blood DNA Kit AS1400 *(ns)*  16S rRNA gene sequencing  V3-V4, ns  MiSeq *(Illumina)*  300, ns  ns | d 1 of hospitalisation  every 2 d during hospitalisation  1, 3, 6 m after hospitalisation |  |  | | • ns  • **Microbiota age**  not affected by ABX but increased during recovery period, reaching a higher level compared with controls 1 m after ABX | • Longitudinal sampling  • Small cohort²  • Dose of ABX ns  • Frequency of ABX ns  • Primers ns  • Sequencing depth ns  • Database for taxonomic identification ns |
|  | **Amoxicillin** (n=20)  ns, ns, mean 5 d  Oral  ABX/controls: no previous ABX |  |  | • **Decreased diversity during ABX** in ABX group  • Recovered rapidly  • No difference 6 m after ABX (inverse Simpson index ns)  • ns | **Relative abundance ABX groups vs. controls** (time point, fold-change) | |  |  |
|  |  |  |  |  | **Order**  Enterobacteria (during ABX, 6)  **Phylum**  Firmicutes (after ABX, 2)  **Family**  *Veillonellaceae* (after ABX, ns) *Ruminococcaceae* (after ABX, ns) *Lachnospiraceae* (after ABX, ns)  *Clostridiaceae* (after ABX, ns)  **Genus**  *Megasphaera* (after ABX, 100)  *Dialister* (after ABX, 20)  *Coprococcus* (after ABX, 10) | **Family**  *Coriobacteriaceae* (during ABX, 77)  *Bacteroidaceae* (during ABX, 42)  *Clostridiaceae* (during ABX, 37)  *Veillonellaceae* (during ABX, 37)  *Streptococcaceae* (during ABX, 4)  **Genus**  *Bifidobacterium* (during ABX, 2)  *Enterococcus* (during ABX, ns) |  |  |
|  | **Macrolides** (n=20)  ns, ns, mean 5 d  Oral  ABX/controls: no previous ABX |  |  | • ns  • ns | **Genus**  *Subdoligranulum* (after ABX, 26)  *Salmonella* (after ABX, 45)  *Bacillales* (after ABX, 45)  *Alphaproteobacteria* (after ABX, 25) | **Genus**  *Bifidobacterium* (after ABX, 59) |  |  |
| Gasparrini *et al.* (54)  2019  USA  Single centre, prospective  cohort study (2b) | 58 (41, 17)  437 (ns, ns)  Hospitalised preterm neonates receiving ABX, preterm neonates not receiving ABX as controls  53%  ABX group median GA 25 w (IQR 24-27 w), controls median GA 36 w (IQR 36-37 w)  **Various ABX** (Ampicillin, cefotaxime, gentamicin, meropenem, vancomycin, others)  ns, ns, ns  ns  ABX: ns  Controls: no previous ABX  ns | Stool, ns  PowerSoil DNA Isolation Kit *(MoBio Laboratories)*  Shotgun metagenomic sequencing, Nextera DNA Library Prep Kit *(Illumina)*  NextSeq *(Illumina)*  150, 2.5 mio reads/sample  NCBI Reference Sequence Database, MetaPh1An2 | 5-10 times between birth and 21 m of life | • **Lower diversity** in ABX group vs. controls (at 1, 2, 4, 5, 8, 9 m of life, Shannon index ns)  • As the number of ABX courses increases, diversity decreases  • High beta-diversity  (dissimilar composition between ABX group and controls (ns, Bray Curtis index ns)  • Dissimilar composition between neonates receiving ABX in the first w of life only and neonates receiving ABX in the first w of life and after the first w of life (ns, Bray Curtis index ns)) | **Relative abundance ABX group vs. controls** (time point in d of life) | | • Higher abundance of **ARGs** in ABX group including ARGs to ABX rarely used in neonates (ciprofloxacin, chloramphenicol) and to multidrug-resistant bacteria (tigecycline, colistin); prolonged carriage of e.g. *oqxA, oqxB, catI, fosA5 and cdeA*), near absence of e.g. *abeM*; stool with high abundance of ARGs dominated by few species (especially *Escherichia coli*)  • **Main factors influencing microbiota development:** ABX, GA at birth, chronological age | • Longitudinal sampling  • Long follow up⁶  • Analysis on species level  • No separate analysis of different ABX  • Dose of ABX ns  • Frequency of ABX ns  • Duration of ABX ns  • Route of administration of ABX ns  • No information on interval without ABX before inclusion for ABX group  • Storage medium ns  • Storage temperature ns  • Prematurity  • Hospitalisation |
|  |  |  |  |  | **Family**  *Enterococcae* (1-4)  *Enterobacteriaceae* (1-3)  **Genus**  *Enterococcus* (1-4)  *Staphylococcus* (0-2)  *Veillonella* (1-4)  **Species**  *Staphylococcus epidermidis* (0-1)  *Enterococcus faecialis* (1-4)  *Veillonella parvula* (1-4)  *Veillonella* unspecified (1-4)  *Klebsiella oxytoca* (1-2)  *Escherichia coli* (1-4)  *Bifidobacterium breve* (4-5) | **Family**  *Bifidobacteriaceae* (1-12)  **Genus**  *Bifidobacterium* (1-5)  *Prevotella* (8-16)  **Species**  *Bifidobacterium bifidum* (2-6)  *Bifidobacterium breve* (0, 2, 3)  *Bifidobacterium longum* (1-9) |  |  |
| Bourke *et al.* (36)  2019  Zimbabwe  Multi-centre,  non-blinded, non-placebo-controlled,  randomised controlled trial  (1b) | 72 (36, 36)  140 (ns,ns)  Non-hospitalised HIV-positive children receiving antiretroviral therapy randomised to continuing or stopping prophylactic ABX  ns  Median 7.9 y (IQR 4.6-6.1)  **Trimethoprim/sulfamethoxazole**  5-15 kg 200/40 mg/d sulfamethoxazole/trimethoprim, 15-30 kg 400/80 mg  >30 kg 800/160 mg/d, 1x/d, prophylactic  Oral  ABX/controls: no ABX for a median of 2 y pre-randomisation  ns | Stool, ns  MoBio DNA Extraction Kit (*Qiagen*)  Shotgun metagenomic sequencing, Illumina TruSeq  Nano DNA Library Prep kits *(Illumina)*  HiSeq *(Illumina)*  125; 10,507,352-11,074,046 reads/sample  MetaPhlan2  HUMANn2 (functional gene and metabolic pathway analysis) | 84 and 96 w after randomisation | • **No difference**  • **No difference** | **Relative abundance ABX group vs. controls** (time point in w after randomisation) | | • ns  • **Microbiome function**  mevalonate pathway I less abundant in the ABX group  • **Intestinal inflammation**  Association between lower fecal inflammation and microbiom findings (abundance differences, function) | • Longitudinal sampling  • Analysis on species level    • Non-blinded  • No placebo  • Sex distribution of cohort ns  • Long interval between randomisation and sampling⁸  • Storage medium ns  • Storage temperature ns |
|  |  |  |  |  | **Species**  *Alistipes onderdonkii* (96)  *Eggerthella lenta* (96)  *Clostridium bartlettii* (96)  *Haemophilus parainfluenzae* (96)  *Streptococcus mutans* (96)  *Streptococcus parasanguinis* (96)  *Streptococcus vestibularis* (96) | **Family**  *Enterobacteriaceae* (96) |  |  |
| D`Souza *et al.* (47)  2019  South Africa  Multi-centre,  non-blinded, non-placebo-controlled,  randomised controlled trial (1b) | 63 (34, 29)  163 (ns, ns)  HIV–exposed, uninfected (HEU) infants receiving prophylactic ABX; HEU infants not receiving ABX  ns  6 w at 1^st^ sample  **Trimethoprim/sulfamethoxazole**  < 5 kg 20/100 mg/d trimeth- oprim/sulfamethoxazole 1x/d, 5-15 kg 40/200 mg/d, 1x/d, prophylactic  Oral  ABX/controls: no previous antibiotics  ns | Stool, disposable wooden stick, cryovials, freezing previous 6- 8 h, then -80 °C  PowerSoil DNA Isolation Kit *(MoBio Laboratories)*  Shotgun metagenomic sequencing, Nextera DNA Library Prep Kit (*Illumina)*  NextSeq *(Illumina)*  150; 3,000,000 reads/sample  MetaPh1An2  HUMANn2 (metabolic pathway analysis), ShortBRED (ARG) | 6 w, 4, 6 m of life | • **Longitudinal increase** in ABX group (ns, Shannon index ns)  • **Microbial taxa diversity lower** than in controls (all time points, Bray Curtis index ns) | **Relative abundance ABX group vs. controls** | | • **ARG genes with increased abundance in ABX groups** (after ABX) Trimethoprim *dfr*  Sulfamethoxazole *sul*  • **Functional pathways**  No difference between ABX and controls  • **HIV**  maternal CD4 T-cell count and reported illnesses not associated with microbiota findings | • Longitudinal sampling  • Non-blinded  • No placebo  • Sex distribution of cohort ns |
|  |  |  |  |  | No difference | No difference |  |  |
| D’Agata *et al.* (42)  2019  USA  Single centre, prospective cohort study (2b) | 47 (23, 24)  337 (ns, ns)  Hospitalised preterm neonates receiving ABX (reason ns), hospitalised neonates not receiving ABX as controls  55%  Mean GA 28 w (ns)  **ns**  ns, ns, ns  ns,  ns  ns | Stool, ns, -80°C  MoBio Power Fecal DNA extraction kit (*Qiagen*)  16S rRNA gene sequencing  V4, ns  200, ns  Miseq (*Illumina*)  QIIME | Weekly samples in the first 6 w of life | • ns  • ns | **Relative abundance ABX group vs. controls** (time point in d of life) | | • ns  • - | • Longitudinal sampling  • ABX ns  • Dose of ABX ns  • Frequency of ABX ns  • Duration of ABX ns  • Route of administration of ABX ns  • No information on interval without ABX/maternal ABX before inclusion  • Sequencing depth ns |
|  |  |  |  |  | **Genus**  *Proteus* (3-6)  *Citrobacter* (3-6)  **Species**  *Clostridium perfringens* (5-6) | **Species**  *Clostridium perfringens* (3-4) |  |  |
| Fouhy *et al.* (51)  2019  Ireland  Single centre, prospective cohort study (2b) | 70 (ns, ns)  159 (ns, ns)  Non-hospitalised children receiving ABX (reason ns), healthy children as controls  43%  1 y at 1^st^ sample  **ns**  ns, ns, ns  ns,  ns  ns | Stool, ns, 4°C until transfer to labroratory, then -80°C  QIAmp Fast DNA Stool Mini Kit (*Qiagen*)  16S rRNA gene sequencing  V4-V5, forward primer 5’ TCGTCGGCAGCGTCAGATGTGTATAAGAGACAG AYTGGGYDTAAAGNG, reverse primer 5’GTCTCGTGGGCTCGGAGATG TGTATAAGAGACAGCCGTCAATTYYTTTRAGT  500, ns  MiSeq (*Illumina*)  SILVA | 1, 2, 4 y of life | • ns  • **No difference** at 1 y | ns | ns | • ns  • - | • Longitudinal sampling  • Non-hospitalised children, healthy controls  • ABX ns  • Dose of ABX ns  • Frequency of ABX ns  • Duration of ABX ns  • Route of administration of ABX ns  • No information on interval without ABX/maternal ABX before inclusion  • Sequencing depth ns |
| D’Haens et al. (45)  2019  Netherlands  ns, prospective cohort study (2b) | 19 (14, 5)  ns (ns,ns)  Hospitalised preterm neonates receiving ABX as a prophylaxis for central venous line removal‐associated sepsis, hospitalised preterm neonates not receiving ABX  ns  Mean GA 28 w (SD 1 w)  **Vancomycin**  10 mg/kg, 2 doses  ns  ABX group/controls: “Most” neonates received amoxicillin/ceftazidime before  ns | Stool, ns, ns  ns  16S rRNA gene sequencing  V3-V4  300, 13776 samples/read  MiSeq (*Illumina*)  ns | 5 d before ABX, 7 d after ABX, 6 w of life | • **Lower diversity at d 7** **compared to before** (5 d before ABX, 7 d after ABX, Chao 1, phylogenetic diversity ns)  • **No difference** between ABX group and controls **at 6 w** | **Relative abundance** (before ABX/d 7) | | • ns  • - | • Longitudinal sampling  • Sample number ns  • Sex distribution ns  • Route of administration of ABX ns  • Storage medium ns  • Storage temperature ns  • DNA extraction kit ns  • Database for taxonomic identification ns  • “Most” neonates received previous ABX |
|  |  |  |  |  | **Genus**  *Staphylococcus* (ns) | **Family**  *Commamonadaceae* (2.5/0.0)  **Genus**  *Pseudomonas* (3.5/0.05)  *Bifidobacterium* (ns) |  |  |
| Doan *et al.* (103)  2018  Niger  Community-based,  double-blinded, placebo-controlled,  cluster- randomised controlled trial (1b) | 20 (10, 10)¹  20 (10,10)  Healthy children randomised to ABX or placebo  51%  Mean 33 m (range 1-60 m)  **Azithromycin**  20 mg/kg, 1x every 6 m  Oral  ‘relatively antibiotic-naïve’ children  ns | Rectal swabs, sterile tube, immediately placed on ice, storage at -20 °C in Niger, then -80 °C  Norgen stool DNA isolation kit *(Norgen)*  Shotgun metagenomic sequencing, NEBNext Ultra II DNA Library Prep Kit (*New England BioLabs)*  HiSeq 4000 *(Illumina)*  125, ns  NCBI database | 6 m after ABX | • **Lower diversity** in ABX group vs. controls (at 6 m, Shannon index 22.2/26.7; inverse Simpson index 9.1/12.4)  • **Lower dissimilarity** within the ABX group compared to controls (at 6 m, Euclidean distance ns) | ns | ns | • ns  • **Other taxonomic groups**  No difference in beta diversity of fungi, viruses, nematodes, plathelminth, ciliophora, apicomplexa, Amoebozoa between ABX group and controls  • **Gamma diversity**  Lower in ABX group vs. controls | • Healthy children  • Placebo-controlled  • Cross-sectional design  • Small cohort²  • Low sample number⁴  • Long interval between randomisation and sampling ⁸  • Sequencing depth ns |
| Oldenburg *et al.* (79)  2018  Burkina Faso  Community-based, double-blinded, placebo-controlled,  randomised controlled trial (1b) | 124 (93,31)  239 (179, 60)  Healthy children randomised to different ABX or placebo  54%  Median 36 m (IQR 21-51 m)  ns | Rectal swabs, sterile tube, ambient temperature, then -80 °C  Norgen stool DNA isolation kit  *(Norgen)*  16S rRNA gene sequencing  V3-V4  ns  ns, ns  GreenGenes | 0 d (before ABX)  5 d after ABX |  | **Relative abundance ABX group vs. controls** (no p-values provided) | | • ns  • - | • Longitudinal sampling  • Healthy children  • Placebo-controlled  • Short follow up⁷  • Relatively low doses of ABX, because no established infections  • Rural area with high morbidity and mortality  • Short interval without ABX before inclusion in 7% of children⁹  • Sequencing platform, length and depth ns  • No p-values provided for relative abundance analysis |
|  | **Amoxicillin** (n=31)  50 mg/kg/d, divided into 2 doses, 5 d  Oral  ABX/controls: 7% had ABX previous < 1 m |  |  | • **No difference**  • ns | **Genus**  *Fusobacterium*  *Catenibacterium*  *Succinivibrio* | **Genus**  *Treponema*  *Sutterella*  *Oscillospira* |  |  |
|  | **Azithromycin** (n=31)¹  10 mg/kg/d, 1x/d on d 1, then 5 mg/kg, 1x/d for 4 d  Oral  ABX/controls: 7% had ABX previous < 1 m |  |  | • **Lower diversity** in ABX group vs. controls (d 5, inverse Simpson index 6.6/9.8)  • ns | **Genus**  *Klebsiella*  *Finegoldia*  *Butyricimonas*  *Anaerococcus*  *Bacteroides* | **Genus**  *Treponema*  *Sutterella*  *Succinivibrio*  *Ruminobacter*  *Anaerovibrio* |  |  |
|  | **Cotrimoxazole** (n=31)  240 mg/d, 1x/d, 5 d  Oral  ABX/controls: 7% had ABX previous < 1 m |  |  | • **No difference**  • ns | **Genus**  *Clostridium* | **Genus**  *Roseburia* |  |  |
| Wei *et al.* (95)  2018  Denmark  Single centre, double-blinded, placebo-controlled  randomised controlled trial (1b) | 59 (29, 30)  116  Hospitalised or non-hospitalised children with recurrent asthma-like symptoms randomised to ABX or placebo  36%  Mean 2 y (SD 0.6)  **Azithromycin**  10 mg/kg/d, 1x/d, 3 d  Oral  ns  ns | Stools, ns  PowerMag Soil DNA Isolation Kit (Eppendorf)  16S rRNA gene sequencing  515F, 806R  MiSeq *(Illumina)*  250, ns  GreenGenes | 14 d after randomisation, 13-39 m after ABX | • **Lower diversity** in ABX group vs. controls (**at d 14**, Shannon index 2.96/3.41)  • **No difference at second sampling**  • **High beta-diversity**  (dissimilar composition between ABX group and controls (at d 14, UniFrac distances R2 = 4.2%), no difference at second sampling) | **Relative abundance ABX group vs. controls** (time point in d after randomisation) | | • ns  • **Asthma**  Azithromycin reduced episode duration by half. | • Longitudinal sampling  • Placebo-controlled  • Long follow up⁶  • No information on interval without ABX before inclusion  • Storage medium ns  • Storage temperature ns  • Sequencing depth ns |
|  |  |  |  |  | **Genus**  *Clostridium_XlVa* (14) | **Phylum**  Actinobacteria (14)  **Order**  Bifidobacteriales (14)  **Family**  *Bifidobacteriaceae* (14)  *Clostridiaceae_1* (14)  **Genus**  *Bifidobacterium* (14)  *Clostridium_sensu_stricto* (14)  *Dialister* (14) |  |  |
| Tapiainen *et al.* (92)  2018  Finland  Single centre, prospective cohort study (2b) | 76 (29, 47)  ns (ns, ns)  Hospitalised neonates receiving ABX (reason ns), hospitalised neonates not receiving ABX as controls  51%  Mean GA 40 w (SD 1 w)  **Various ABX** (e.g. penicillin (plus aminoglycoside)  ns, ns, ns  ns,  ns  ABX group: 100% (*Lactobacillus reuteri)*  Controls: 0% | Stool, ns, -20°C  QIAamp Fast DNA stool mini kit (*Qiagen*), MOBio Powersoil DNA Extraction kit  16S rRNA gene sequencing  V4-V5, R926, F519  Ion Torrent  ns,ns  Human Intestinal 16S rRNA gene reference taxonomy | Daily samples during hospitalisation, 6 m of life | • **No difference**  • **High beta-diversity** (dissimilar composition between ABX group and controls (during hospital stay and at 6 m, Bray Curtis index ns) | **Relative abundance ABX group vs. controls** (time point in d or w of life) | | • ns  • - | • Longitudinal sampling  • overlappping children with Ainonen et al.⁵  • Reason for ABX ns  • Dose of ABX ns  • Frequency of ABX ns  • Duration of ABX ns  • Route of administration of ABX ns  • Sequencing length ns  • Sequencing depth ns |
|  |  |  |  |  | **Phylum**  Firmicutes (2 d)  **Genus**  *Lactobacillus* (2 d, 4 d) | **Phylum**  Bacteroidetes (2 d, 6 m)  **Genus**  *Bacteroides* (2 d, 4 d) |  |  |
| Dahl et al. (43)  2018  Norway  Single centre, cross-sectional analysis (from a prospective cohort study)  (3b) | 519 (-,-)  1247 (-,-)  Hospitalised and non-hospitalised preterm and term neonates receiving and not receiving ABX (reason for ABX ns)  47%  ns (range GA 23-44 w)  **ns**  ns, ns, ns  ns  26% maternal ABX  ns | Stool, ns, -20°C  Customised DNA extraxtion protocol (FastPrep-tubes and instrument (Qbiogene), silica particles (Merck), Biomek 2000 Workstation)  16S rRNA gene sequencing  V4, 515F, 806R  ns, ns  HiSeq (Illumina)  Greengenes | 10 d of life | • **Diversity not associated with ABX** at 10 d  • ns | Not done | Not done | • ns  • - | • Large cohort¹  • Large sample size³  • Cross-sectional analysis  • No control group  • ABX ns  • Dose of ABX ns  • Frequency of ABX ns  • Duration of ABX ns  • Route of administration of ABX ns  • No information on interval without ABX before inclusion for ABX group  • Sequencing depth ns  • Sequencing length ns |
| Doan *et al.* (46)  2017  Niger  Community-based,  double-blinded, placebo-controlled,  cluster-randomised controlled trial (1b) | 80 (40, 40)¹  80 (40, 40)  Healthy children randomised to ABX or placebo  61%  Mean 3 y (SD 2-4 y)  **Azithromycin**  20 mg/kg, single dose  Oral  ‘relatively antibiotic-naïve’ children  ns | Rectal swabs, sterile tube, immediately placed on ice, storage at -20°C in Niger, then -80°C  Norgen stool DNA isolation kit *(Norge)*  16S rRNA gene sequencing  V3–V4, ns  ns  600, 234.845 reads/sample  Greengenes | 0 d (before ABX), 5 d after ABX | • **Lower diversity** in ABX group vs. controls (at d 5, Shannon index 10.6/15.4)  • **No difference** | **Relative abundance ABX group vs. controls** (time point in d after ABX) | | • ns  • No change in abundance of Clostridium after ABX  • **Gamma-diversity**  Decreased in ABX group after ABX | • Longitudinal sampling  • Healthy children  • Placebo-controlled  • Short follow up⁷  • Primers ns  • Sequencing platform ns |
|  |  |  |  |  | **Genus**  *Blautia* (5) | **Genus**  *Anaerovibrio* (5)  *Peptoniphilus* (5)  *Succinivibrio* (5)  *Megasphaera* (5) |  |  |
| Parker *et al.* (80)  2017  India  Community-based,  double-blinded,  placebo-controlled, randomised controlled trial (1b) | 120 (60, 60)  240 (120,120)  Healthy children randomised to ABX or placebo  ns  ns (range 6-11 m)  **Azithromycin**  10 mg/kg/d, 1x/d. 3d  Oral  ABX/controls: 25% had ABX previous < 1 m before randomisation, 9% had other ABX during the study  ns | Stool, ns  QIAamp DNA Stool Mini Kit *(Qiagen)*  16S rRNA gene sequencing  V4, 515F, 806R  MiSeq *(Illumina)*  ns, 7500 reads/sample  ns | 0 d (before ABX), 12 d after ABX | • **Lower richness** in ABX group than in controls (at d 12, OTU count 68.1/ 73.6)  • **No difference in diversity**  • **High beta-diversity**  (dissimilar composition between ABX group and controls (at d 12, Unifrac distance R2 0.02)) | **Relative abundances ABX group vs. controls** (time point in d after randomisation) | | • ns  • **Diarrhoea**  No difference in diarrhoea prevalence at d 14 between groups  • **Prophylactic effect/therapeutic effect**  ABX group less likely to become colonised by bacterial pathogens between d 0 and 14 (e.g. *Escherichia coli, Campylobacter*); reduced prevalence of bacterial enteropathogens (including ETEC) by d 14  • **Microbiota age**  ABX did not influence microbiota age (Unifrac distance to adult samples) | • Samples before and after ABX  • Healthy children  • Placebo-controlled  Analysis on OTU species level  • Short follow up⁷  • Sex distribution of cohort ns  • Short interval without ABX before inclusion  • Storage medium ns  • Storage temperature ns  • Sequencing length ns  • Database for taxonomic identification ns |
|  |  |  |  |  | **Order**  Lactobacillales (12)  **Family**  *Clostridiaceae* (12) | **Phylum**  Proteobacteria (12)  Verrucomicrobia (12)  **Class**  Verrucomicrobiae (12)  Betaproteobacteria (12)  **Genus**  *Escherichia* (12)  *Akkermansia* (12)  *Peptostreptococcus* (12)  *Campylobacter* (12)  **OTU**  *Escherichia coli 8711* (12)  *Escherichia coli 17709* (12)  *Akkermansia muciniphila* (12)  *Lactobacillus mucosae* (12)  *Bacteroides fragilis* (12)  *Peptostreptococcus* (12) |  |  |
| Zwittink *et al.* (101)  2018  Netherlands  Single centre,  prospective cohort study (2b) | 15 (10, 5)  95 (ns, ns)  Hospitalised preterm neonates with clinical suspicion of bacterial infection receiving ABX, hospitalised preterm neonates as controls  20%  Mean GA 36 w (range 34-37 w)  **Amoxicillin plus ceftazidime**  ns, ns, < 3 d (5), > 5 d (5)  Intravenous  ns  ns | Stool, temporally stored at -20 °C, then -80 °C  Repeated bead beating plus phenol/chloroform method  16S rRNA gene sequencing  V3-V5, 375F, 926Rb  454 pyrosequencing *(Roche)*  ns, 4085 reads/sample  SILVA | 6-7 samples between birth and 6 w of life | • ns  • ns | **Relative abundances ABX group vs. controls** (time point in w of life) | | • ns  • **ABX duration** associated with differences between groups | • Longitudinal sampling  • Small cohort²  • Dose of ABX ns  • Frequency of ABX ns  • No information on interval without ABX before inclusion  • Sequencing length ns |
|  |  |  |  |  | **Genus**  *Enterococcu*s (2)  *Clostridium* (ns) | **Genus**  *Bifidobacterium* (< 3 for those treated <3 d)/< 6 for these treated > 5 d) |  |  |
| Forsgren *et al.* (50)  2017  Finland  Multi-centre, prospective cohort study (2b) | 118 (25, 93)  ns (ns,ns)  Hospitalised preterm and term neonates receiving ABX (reason ns), hospitalised preterm and term neonates not receiving ABX as controls  30%  Median GA 39 w (range 33–42)  **ns**  ns, ns, ns  ns  21% maternal ABX  Total 48% (ABX, controls ns) | Stool, ns, -80°C  KingFisher DNA extraction system (*Thermo Fisher Scientific*), InviMag Stool DNA kit (*Stratec Molecular*)  qPCR for *Bifidobacterium Bidiobacterium adolescentis, Bidiobacterium bifidum, Bidiobacterium breve, Bidiobacterium catenulatum, Bidiobacterium lactis, Bidiobacterium longum, Bidiobacterium infantis; Clostridium coccoides, Clostridium leptum, Clostridium difficile, Clostridium perfringens, Staphylococcus aureus, Akkermansia muciniphila* | immediately after birth, 2-4 w of life, 6 m of life | • ns  • ns | **Prevalence ABX group vs. controls (OR)** | | • ns  • - | • -  • Reason for ABX ns  • ABX ns  • Dose of ABX ns  • Frequency of ABX ns  • Duration of ABX ns  • Route of administration of ABX ns  • No information on interval without ABX before inclusion for ABX group  • Storage medium ns |
|  |  |  |  |  |  | **Species**  *Bifidobacterium bifidum (0.27)*  *Bifidobacterium breve (0.27)*  *Bifidobacterium lactis (0.43)*  *Bifidobacterium longum (0.14)* |  |  |
| Itani *et al.* (65)  2017  Lebanon  Single centre, prospective cohort study (2b) | 66 (46,20)  127 (ns, ns)  Hospitalised preterm neonates receiving ABX for various infections,  hospitalised preterm neonates not receiving ABX  42%  Mean GA 33 w (SD ±2.1)  **Various ABX** (e.g. ampicillin and cefotaxime, amikacin, vancomycin, imipenem, ceftazidime)  ns, ns, 61% < 48 h, 39% > 7 d  ns  ns  ns | Stool, sterile tube, -80°C  bead-beating method  qPCR  for *Staphylococcus,*  *Enterococcus,*  *Enterobacteriaceae,*  *Lactobacillus/Leuconostoc /Pediococcus,*  *Bifidobacterium.*  *Prevotella* group.  *Clostridium coccoides* group*.*  *Clostridium leptum* group.  *Clostridium* of Cluster I.  *Clostridium* of Cluster XI  various primers | Weekly during hospitalization (ns) | • ns  • ns | **Colonisation rate ABX vs. controls** (time point in w of lfe) | | • ns  • - | • -  • Dose of ABX ns  • Frequency of ABX ns  • Duration of ABX ns  • Route of administration of ABX ns  • No information on interval without ABX before inclusion  • No separate analysis of different ABX |
|  |  |  |  |  | **Genus**  *Clostridium* (2,3) | **Genus**  *Lactobacillus* (1)  *Bacteroides/Prevotella* (1)  *Clostridium* (1) |  |  |
| Zhu et al. (100)  2017  China  Single centre, prospective cohort study (2b) | 36 (24, 12)  ns (ns, ns)  Hospitalised preterm neonates receiving two different ABX (reason ns), hospitalised preterm neonates not receiving ABX as controls  61%  Mean GA 34 w (ns) | Stool, sterile tube, -80°C  QIAamp FAST DNA Stool Mini-Kit (Qiagen)  16S rRNA gene sequencing  V3-V4, 338F, 5′-ACTCCTACGG-GAGGCAGCA-3′, 806R, 5′-GGACTACHVGGGTWTCTAAT-3′  250, mean 16.136 samples/read  MiSeq (Illumina)  RDP, SILVA | 3, 7 d of life |  | **Relative abundance** (d 3 vs. d 7 or PM/PT vs. controls) | | • ns  • **Metabolic pathways**  No changes at d 3, but significant changes at d 7, L-tyrosine and citric acid became dominant in PM group | • -  • Small cohort²  • Sample number ns  • Route of administration of ABX ns  • No information on interval without ABX/maternal ABX before inclusion for ABX groups  • Short follow-up⁷ |
|  | **Penicillin plus moxalactam** (PM, n=12)  20U/kg/d, divided into 2 doses, 7 d  ns  ABX: ns  Controls: no ABX  0% |  |  | • **Lower diversity at d 7 than at d 3** (Shannon ns)  • No difference in diversity between PM and controls or between PM and PT  • **High beta-diversity** (dissimilar composition between PM and controls (d 3, principal coordinates analysis ns) | **Phylum**  Proteobacteria (d 3 vs. d 7)  Bacteroidetes (PM vs. controls, d3 & d 7)  Actinobacteria (PM vs. controls, d 3)  **Genus**  *Klebsiella* (d 3 vs. d 7)  *Lactobacillus* (PM vs. controls, d 3)  *Sphingomonas* (PM vs. controls, d 3)  *Bacteroides* (PM vs. controls, d 3)  *Lactobacillus* (PM vs. controls, d 3)  *Escherichia*-*Shigella* (PM vs. controls, d 7) | **Phylum**  Firmicutes (d 3 vs. d 7)  **Genus**  *Clostridium* (PM vs. controls, d 3) |  |  |
|  | **Piperacillin-tazobactam** (PT, n=12)  150 mg/kg/d, divided into 2 doses, 7 d  ns  ABX: ns  Controls: no ABX |  |  | • **Lower diversity at d 7 than at d 3** (Shannon ns)  • No difference in diversity between PT and controls or between PM and PT  • **High beta-diversity** (dissimilar composition between PT and controls (d 3, principal coordinates analysis ns) | **Phylum**  Firmicutes (d 3 vs. d 7)  Bacteroidetes (PT vs. controls, d 3 & d 7)  Actinobacteria (PT vs. controls)  **Genus**  *Klebsiella* (d 3 vs. d 7)  *Enterococcus* (PT vs. PM, d 3 & d 7) | **Phylum**  Proteobacteria (d 3 vs. d 7)  **Genus**  *Klebsiella* (PT vs. controls, d 7)  *Clostridium* (PT vs. PM, d 7) |  |  |
| Korpela *et al.* (70)  2016  Finland  Community-based,  prospective cohort study (2b) | 142 (ns, ns)  257 (ns, ns)  Non-hospitalised children receiving ABX for various infections (88% respiratory), non-hospitalised children who had not received ABX for > 2 y as controls  ns  ns (range 2-7 y)  ns (included children are part of a larger cohort, which randomised children to receiving normal milk or milk plus *Lactobacillus rhamnosus* GG) | Stool, collected at home, immediate transport, then -70 °C  Promega Wizard Genomic DNA Purification Kit *(Promega)*  16S rRNA gene sequencing  V4–V6, S-D-Bact-0564-a- S-15/S, Univ-1100-a-A-15  454 Titanium *(Roche)*  ns, mean 8801 reads/sample  Greengenes  qPCR (ARG) | 1-2 samples 0-24 m after ABX |  |  | | • **Macrolide resistance** elevated after ABX, declined linearly until it reached a low baseline level 6-12 m after ABX  • **Specific genes**  Abundance of *ermF* and *ermB* genes correlated negatively with time since the last macrolide course, abundance of bsh genes correlated positively with time since the last macrolide course  • **Age**  Similar microbiota changes in 2–4- and 5–7-y-olds  •**Other health outcomes**  Early-life ABX was associated with asthma & ↑ BMI | • (Partly) longitudinal sampling  • Non-hospitalised children  • Long follow up⁶  • Sex distribution of cohort ns  • Dose of ABX ns  • Frequency of ABX ns  • Duration of ABX ns  • Route of administration of ABX ns  • Sequencing length ns |
|  | **Macrolides** (azithromycin, clarithromycin, n=ns)  ns, ns, ns  ns  ABX: mean of 3-13 lifetime ABX courses  Controls: no ABX previous 2 y |  |  | • **Lower richness** in ABX group observed (between <6 and 12-24 m, number of species-like phylotypes ns)  • **Distinct composition** on phylum- and genus-level in ABX group (at 6 m, principal coordinates analysis ns) | **Relative abundance ABX groups vs. controls** (time point in m after ABX fold-change) | |  |  |
|  |  |  |  |  | **Phylum**  Bacteroidetes (<6 2)  Proteobacteria (<6 2)  **Order**  Bacteroidales (<6 2)  Lactobacillales (6-24 2)  **Genus**  *Eggerthella* (<6 11)  *Bacteroides* (<6 2)  *Parabacteroides* (<6, 2)  *Eubacterium* (<6, 3)  *Clostridium* (<6, 3)  *Parabacteroides* (6-12, 1.1)  *Unassigned Rikenellaceae* (6-12, 1.2)  *Dialister* (12-24, 1.1) | **Phylum**  Actinobacteria (<6, 0.2)  **Order**  Bifidobacteriales (<6, 0.2)  Coriobacteriales (<6, 0.2)  Clostridiales (<6, 0.5)  Gemellales (6-12, 0.8)  **Genus**  *Bifidobacterium* (<6, 0.2)  *Unassigned*  *Collinsella* (<6 0.3; 6-12, 0.7)  *Unassigned Gemellaceae* (6-12, 0.8)  *Dialister* (6-12, 0.9)  *Collinsella* (12-24, 0.5) |  |  |
|  | **Penicillins** (amoxicillin with or without clavulanic acid and penicillin V, n=ns)  ns, ns, ns  ns  ABX/controls: mean of 3-13 lifetime ABX courses  Controls: no ABX previous 2 y |  |  | • **Lower richness** in ABX group observed (between <6 and 6-12 m, number of species-like phylotypes ns)  • **Distinct composition** on phylum- and genus-level in ABX group (6 m, 6-12 m, 12-24 m, principal coordinates analysis ns) | **Phylum**  Bacteroidetes (<6 1.5)  **Genus**  *Parabacteroides* (<6 3)  *Unassigned Rikenellaceae* (<6 1.2)  *Dialister* (<6 1.1)  *Parabacteroides* (12-24 1.61)  *Dialister* (ns 1.43) | **Phylum**  Unassigned Actinobacteria (<6 0.7)  **Order**  Gemellales (<6 0.8; 6 -12 0.83; 12-24 0.84)  **Family**  *Unassigned Gemellaceae* (<6 0.8; 6-12 0.82 ; 12-24 0.84)  **Genus**  *Collinsella* (<6 0.8; 6-12 0.88 ; 12-24, 0.60)  *Lactobacillus* (<6 0.1; 6-12 0.09)  *Parabacteroides* (6-12, 0.96) |  |  |
| Romano-Keeler *et al.* (84)  2016  USA  Single centre, cross-sectional analysis (3b) | 24 (12,12)  44 (ns, ns)  Hospitalised term and preterm neonates with NEC and other intestinal diseases receiving vancomycin, hospitalised term and preterm neonates with NEC and other intestinal diseases receiving other ABX than vancomycin as controls  54%  Mean GA 29 w (range 24-39 w)  **Vancomycin**  ns, ns, ns  ns  ABX group: 100% also had other ABX  Controls: 92% had other ABX  ns | Intestinal tissue, stool, sterile container, immediate cryopreservation  Modified Qiagen protocol  16S rRNA gene sequencing  V1-V3, 5F (5’-TGGAGAGTTTGATCCTGGCTCAG-3’) and 532R (5’-TACCGCGGCTGCTGGCAC-3’)  ns, > 400 reads/sample  454 FLX Titanium *(Roche*)  SILVA | Intestinal tissue during surgery at a mean age of 25 d, first post-operative stool | • **No difference**  • ns | **Relative abundance in vancomycin group compared to other ABX** | | • ns  • - | • -  • Cross-sectional analysis  • Dose of ABX ns  • Frequency of ABX ns  • Duration of ABX ns  • Route of administration of ABX ns  • Sequencing length ns |
|  |  |  |  |  | **Genus**  *Staphylococcus* (intestinal tissue only) |  |  |  |
| Gibson *et al.* (55)  2016  USA  Single centre, prospective cohort study (2b) | 84 (51, 33)  401 (ns, ns)  Hospitalised preterm neonates receiving ABX within the first 24 h after birth (early) and subsequently for various infections, hospitalised preterm neonates only receiving ABX within the first 24 h of life (early) as controls  58%  Median GA 27 w (IQR 25-29 w)  ns | Stool, ns, briefly at 4°C, then -80°C  PowerMax Soil DNA Isolation Kit (*MoBio Laboratories*)  16S rRNA gene sequencing  V4, 515F/806R  250, ns  MiSeq (*Illumina*)  Shotgun metagenomic sequencing  ns  150, 1 mio reads/sample  HiSeq *(Illumina*)  Greengenes, MetaPhlAn 2.0 | 2 d before ABX, 2 d after ABX |  | **Relative abundance ABX group vs. controls** | |  | • Species level analysis  • Storage medium ns  • Library preparation kit for shotgun metagenomic sequencing ns |
|  | **Gentamicin** (early only n=24, early and subsequent ABX n=49)  ns, ns, early only mean duration of 2 d (IQR 1-2) early and subsequent mean duration of 8 d (IQR of 4-13 d)  ns  ns |  |  | • **No difference** | ns | ns | • **Enriched ARG genes after ABX** (Pearson correlation of 1.00)  *evgA*  *emrK* |  |
|  | **Ampicillin** (early only n=30, early and subsequent ABX n=49)  ns, ns, early only mean duration of 2 d (IQR 2-2) early and subsequent mean duration of 2 d (IQR of 2-2 d)  ns  ns |  |  | • **Reduced species** richness before vs. immediately after ABX | ns | ns | • **Enriched ARG genes after ABX** (Pearson correlation of 1.00)  *MFS efflux*  *β-lactamase (unknown)*  *ABC efflux*  *araC*  *emrD* |  |
|  | **Vancomycin** (early only n=5, early and subsequent ABX n=48)  ns, ns, early only mean duration of 0 d (IQR 0-0 d) early and subsequent mean duration of 8 d (IQR of 5-13 d)  ns  ns |  |  | • **Reduced species** **richness** before vs. immediately after ABX | ns | ns | • **Enriched ARG genes after ABX** (Pearson correlation of 1.00)  *evgA*  *emrK* |  |
|  | **Meropenem** (early and subsequent ABX n=19)  ns, ns, IQR of 0-6 d  ns  ns |  |  | • **Reduced species** **richness** before vs. immediately after ABX | **Species**  *Staphylococcus epidermidis* |  | • **Enriched ARG genes after ABX** (Pearson correlation of 1.00)  *mecA*  *norA*  *dfrC*  *fluoroquinolone resistant gyrA*  *qacA* |  |
|  | **Cefotaxime** (early and subsequent ABX n=15)  ns, ns, IQR of 0-1 d  ns  ns |  |  | • **Reduced species** **richness** before vs. immediately after ABX |  | **Species**  *Escherichia coli* | • **Enriched ARG genes after ABX** (Pearson correlation of 1.00)  *β-lactamase (CMY-LAT-MOX)*  *β-lactamase (unknown)*  *MFS efflux*  *Small multidrug resistance*  *ABC efflux*  *robA* |  |
|  | **Ticarcillin–clavulanate** (early and subsequent ABX n=15)  ns, ns, IQR of 0-2 d  ns  ns |  |  | • **Reduced species** **richness** before vs. immediately after ABX | **Species**  *Klebsiella pneumoniae* |  | • **Enriched ARG genes after ABX** (Pearson correlation of 1.00)  *MFS efflux*  *β-lactamase (unknown)*  *ABC efflux*  *araC*  *emrD*  *Antibiotic resistant folP*  *Aminocoumarin resistant parE*  *Mutant porin ompF* |  |
|  | **Clindamycin** (early and subsequent ABX n=12)  ns, ns, IQR of 0-0 d  ns  ns |  |  | • **Reduced species** **richness** before vs. immediately after ABX | ns | ns | • ns |  |
|  | **Cefazolin** (early and subsequent ABX n=9)  ns, ns, IQR of 0-0 d  ns  ns |  |  | • **Reduced species** **richness** before vs. immediately after ABX | ns | ns | • ns |  |
|  | **Trimethoprim-sulfamethoxazole** (early and subsequent ABX n=4)  ns, ns, IQR of 0-0 d  ns  ns |  |  | • **Reduced species** **richness** before vs. immediately after ABX | ns | ns | • ns |  |
| Chernikova *et al.* (41)  2016  USA  ns, prospective cohort study (2b) | 9 (-,-)  49 (-,-)  Hospitalised preterm neonates receiving ABX  33%  Mean GA 26 w (range GA 24-29 w)  **Ampicillin plus gentamicin (plus vancomycin or cefotaxime or nafcillin, or**  **piperacillin/tazobactam or clindamycin or macrodantin)**  ns, ns, range of 2-8 d  intravenous  67% maternal ABX  ns | Stool, sterile container, -80°C  Powersoil bacterial DNA isolation kit (*MoBio*)  16S rRNA gene sequencing  V4-V6, 518F, 1064R  ns, ns  GS-FLX Titanium  SILVA | Weekly during hospitalisation | • **Lower diversity** in neonates receiving ABX for >2 d compared to neonates receiving ABX for < 2 d (all time points, Simpson diversity ns)  • Diversity decreased with increasing duration of ABX  • Diversity increased after the end of ABX  ns | **Relative abundance** (at the day of ABX administration vs. other time points) | | • ns  • - | • Longitudinal sampling  • No control group  • Small cohort²  • Low sample number⁴  • Dose of ABX ns  • Frequency of ABX ns  • Sequencing length ns  • Sequencing depth ns |
|  |  |  |  |  | **Genus**  *Ralstonia*  *Propionibacterium*  *Bradyrhizobium*  *Serratia*  *Bacteroides* |  |  |  |
| Bokulich *et al.* (115)  2016  USA  Single centre,  prospective cohort study (2b) | 43 (25, 18)  ns (ns, ns)  Hospitalised neonates receiving ABX (ear/eye/respiratory infections), hospitalised neonates not receiving ABX as controls  33%  ns  **Various antibiotics** (e.g. penicillins, macrolides, cephalosporins, aminoglycosides)  ns, ns, ns  Intravenous/oral/intramuscular/local  ns  ns | Stool & rectal swabs, sterile containers, placed on ice, stored at -80 °C previous 24-48 h  Power-Soil-htp 96 Well Soil DNA Isolation Kit *(MoBio Laboratories)*  16S rRNA gene sequencing  V4, ns  MiSeq *(Illumina)*  151, ns  Greengenes, RDP | 12-24 h of life  every m in 1^st^ year of life  every 2 m during 2^nd^ and 3^rd^ year of life  whenvever possible before, during and after ABX | • **No difference** before, during or after ABX  • **Difference in composition** between ABX group and controls (ns, permutational MANOVA R2 < 0.01) | **Relative abundance ABX group vs. controls** (time point in m of life) | | • ns  • **Microbiota maturation**  delayed in ABX group (during 6-12 m of life, due to depleted Enterobacteriaceae,  Lachnospiraceae, Erysipelotrichaceae) | • Longitudinal sampling  • Long follow up⁶  • Small cohort²  • Sample size ns  • Mean/median GA ns  • No separate analysis of different ABX  • Dose of ABX ns  • Frequency of ABX ns  • Duration of ABX ns  • No information on interval without ABX before inclusion  • Primers ns  • Sequencing length and depth ns |
|  |  |  |  |  | No difference | **Order**  Clostridiales (3-9)  **Genus**  *Ruminococcus* (3) |  |  |
| Arboleya *et al.* (28)  2016  Spain  Single centre,  prospective cohort study (2b) | 40 (35, 5)  ns (ns, ns)  Hospitalised preterm and term neonates receiving ABX (reason ns), hospitalised preterm and term neonates not receiving ABX  ns  ns  **Various antibiotics**  ns, ns, ns  ns  ns  ns | Stool, ns, immediately at -20°C  16S rRNA gene sequencing  V3, ns  ns, ns  RDP | 24-48 h of life  10, 30, 90 d of life | • ns  • ns | **Relative abundance ABX group vs. controls** (time point in d of life) | | • ns  • - | • Longitudinal sampling  • Small cohort²  • Sample size ns  • Mean/median GA ns  • Sex distribution of cohort ns  • No separate analysis of different ABX  • Dose of ABX ns  • Frequency of ABX ns  • Duration of ABX ns  • Route of administration of ABX ns  • No information on interval without ABX before inclusion  • Storage medium ns  • Primers ns  • Sequencing length and depth ns |
|  |  |  |  |  | **Phylum**  Proteobacteria (30) | **Phylum**  Actinobacteria (30)  Firmicutes (30) |  |  |
| Yassour *et al.* (98)  2016  Finland  Community-based,  prospective cohort study (2b) | 39 (20, 19)¹  1069 (ns, ns)  Non-hospitalised infants receiving ABX for various infections (91% otitis media), healthy infants as controls  44%  2 m at 1^st^ sample  **Various antibiotics**  ns, ns, ns  Oral  ABX: ns  Controls: no (previous) ABX  ns | Stool, ns, -20 °C, shipping on dry ice, then -80 °C  QIAamp DNA Stool Mini Kit (*Qiagen*)  16S rRNA gene sequencing  V4, ns  HiSeq 2500 (*Illumina*)  ns, 48,131 reads/sample  Greengenes  Shotgun metagenomic sequencing  Nextera XT DNA Library Preparation Kit *(Illumina)*  HiSeq 2500 (*Illumina*)  101, 2.5 Gb/sample  MetaPhlAn 2.0  shortBRED (ARG) | every m between 2-36 m of life | • **Lower richness** in ABX group than controls (12-36 m of life, Chao 1 ns)  • Low Bacteroides abundance was associated with a low $\alpha$-diversity  • ABX group had more species dominated by a single-strain compared with controls | No difference | **Genus**  *Clostridium clusters 4 and 14a* (3 y of life)  **Species**  *Eubacterium rectale* | • 11/39 children had ARG (at 2 m of life) before any ABX  • **Chromosomally encoded genes** in 3 cases the abundance of ARG increased during ABX, then decreased  • **Episomally encoded genes (=mobile genes)** abundance increased during ABX, but did not decrease afterwards  • **Longitudinal microbiota stability** ABX children had less stable microbial communities compared with controls  • **Strain patterns**  Single-colonisation species were less similar previous ABX children, while multiple-colonisation species were not different between ABX children and unexposed | • Longitudinal sampling  • Large sample number³  • Long follow up⁶  • Analysis on species level  • Overlapping cohort with Lebeaux *et al.*  • No separate analysis of different ABX  • Dose of ABX ns  • Frequency of ABX ns  • Duration of ABX ns  • No information on interval without ABX in ABX group before inclusion  • Analysis of abundances focussed on Clostridium clusters 4 and 14a as illustrative examples  • Storage medium ns  • Primers ns  • Sequencing length ns |
| Ward *et al.* (94)  2016  USA  Multi-centre,  prospective cohort study (2b) | 166 (ns, ns)  405 (ns, ns)  Hospitalised preterm neonates with and without NEC and term neonates receiving ABX (reason ns), term neonates not receiving ABX as controls  48%  ABX group median GA 26 w (range 23-27 w), controls median GA 39 w (IQR 38-41 w)  **Ampicillin plus gentamicin**  ns, ns, < 6 (short-term (ST), 7-14 d (long-term (LT))  ns  ABX/controls: 72% of preterm infants mother´s had perinatal ABX  ns | Stool, ns  QIAGEN AllPrep DNA/RNA Mini Kit *(Qiagen)*  Shotgun metagenomic sequencing ns  ns  > 80, ns  MetaPhlAn2 | 3-22 d of life | • **Lower in LT group compared with ST group** (at 17-22 d of life, Shannon index 0.91/1.39)  • No difference between LT and ST group at 3-16 d of life  • ns | **Relative abundance ABX group vs. controls** (time point in d of life, ABX group) | | • ns  • **NEC**  Colonisation by Escherichia coli risk factor for development of NEC | • Longitudinal sampling  • Large sample size  • Analysis on species level  • Number of children in ABX group/controls ns  • Dose of ABX ns  • Frequency of ABX ns  • Route of administration of ABX ns  • Short follow up⁷  • Storage medium ns  • Storage temperature ns  • Sequencing platform and depth ns |
|  |  |  |  |  | **ST group**  **Class**  Bacilli (3-9, ST)  **Order**  Clostridiales (17-22, ST)  Bacteroidales (10-16, LT)  **Genus**  *Veillonella* (17-22, ST)  *Klebsiella* (17-22, ST)  *Bacteroides* (3-9, LT)  *Streptococcus (*10-16, LT)  **Species**  *Escherichia coli* (17-22) | No difference |  |  |
| Zhou et al. (99)  2015  USA  Single centre,  cross-sectional analysis (previous a prospective case-control study) (3b) | 26 (ns, ns)  ns (ns, ns)  Hospitalised preterm neonates with and without NEC receiving ABX, preterm neonates not receiving ABX as controls  45%  Mean GA 28 w (range 24-31 w)  **ns** (broad spectrum antibiotics)  ns, ns, ns  Intravenous  ABX/controls: 26% of infants mother´s had perinatal ABX  Controls: no ABX previous 5 days  ns | Stool, specimen bag, 4°C for max. 24 h, then -80 °C  QIAgen QIAamp DNA Stool Mini kit *(Qiagen)*  16S rRNA sequencing  V3-V5, 357F, 926R  ns  ns, > 1000 reads/sample  ns | 5 d after ABX | • **Lower richness** (at d 5, number of bacteria ns) **and diversity** (at d 5, Shannon index ns) in ABX group compared with controls  • ns | ns | ns | • ns  • **NEC**  lower microbial diversity and compared with non-NEC infants | • -  • Cross-sectional design  • Size of ABX group/controls ns  • Small cohort²  • Sample size ns  • No separate analysis of different ABX  • ABX ns  • Dose of ABX ns  • Frequency of ABX ns  • Duration of ABX ns  • Short follow up²  • Sequencing platform and length ns  • Database for taxonomic identification ns  • No abundance analysis |
| Dardas *et al.* (44)  2014  USA  Single centre,  prospective cohort study (2b) | 27  48  Hospitalised preterm neonates receiving ABX (reason ns)  56%  Mean GA 29 w (ns)  **Various antibiotics** (e.g. ampicillin, gentamicin)  ns, ns, 2 d of ABX (short-term, ST) vs. ≥7 d of ABX (long-term, LT)  ns  ns  ns | Rectal swabs, sterile swabs, nucleic acid stabilizing reagent, -80 °C  Zymo Fecal DNA kit *(Zymo Research)*  16S rRNA pyrosequencing  V1-V3, 454  ns  200, ns  GreenGenes | 10 ± 1 and 30 ± 2 d of life | • **Lower richness** (10 d of life, 13.6/12.7) **and diversity** (10 d of life, Shannon index 0.8/0.6) in LT group compared with ST group, no difference at 30 d  • Biggest beta-diversity between LT group at d 10 and ST group at d 30 (d 10 and d 30, Bray Curtis index ns) | **Relative abundance LT vs. ST** (time point in d of life) | | • ns  • - | • -  • No control group  • Small cohort²  • Low sample number³  • No separate analysis for different ABX  • Dose of ABX ns  • Frequency of ABX ns  • Route of administration of ABX ns  • No information on interval without ABX before inclusion  • Short follow up⁷  • Sequencing platform and depth ns |
|  |  |  |  |  | **Phylum**  Bacteroidetes (10) | **Phylum**  Actinobacteria (10, 30)  Proteobacteria (10, 30)  Bacteroidetes (30) |  |  |
| Greenwood *et al.* (58)  2014  USA  Multi-centre,  prospective cohort study (2b) | 74 (61, 13)  ns (ns, ns)  Hospitalised preterm neonates with and without NEC receiving ABX, hospitalised preterm neonates not receiving ABX as controls  44%  Short-term (ST) ABX group median GA 28 w (range 24-32 w), long-term (LT) ABX group median GA 25 w (range 25-31 w), controls median GA 31 w (24-32 w)  **Ampicillin plus gentamicin**  ns, ns, 1-4 d of ABX (ST) vs. 5-7 d of ABX (LT)  ns  ns  ns | Stool, Storage medium ns, refrigerator, then -80°C  Phenol-chloroform or QiaAmp DNA stool kit *(Qiagen)*  16S rRNA sequencing  V3-V5, ns  454 FLX Titanium platform *(Roche)*  ns, 1.3x10^6^ reads/sample  ns | 1, 2, 3 w of life | • **Lower diversity** in ABX group than in controls (at 2 and 3 w of life, inverse Simpson index ns)  • Duration of ABX correlated with the decrease in diversity  • ns | **Relative abundance ABX group vs. controls** (time point in d of life treatment group) | | • ns  • **Other outcomes in ABX group** more cases of NEC, sepsis, or death compared with controls | • Longitudinal sampling  • Sample size ns  • Dose of ABX ns  • Frequency of ABX ns  • No information on interval without ABX before inclusion  • Short follow up⁷  • Primers ns  • Sequencing length ns  • Database for taxonomic identification ns |
|  |  |  |  |  | **Genus**  *Enterobacter* (0-7 ST)  *Enterobacter* (8-14 ST, LT) | **Genus**  *Enterococcus* (0-7 ST)  *Staphylococcus* (0-7 ST) |  |  |
| Rosa *et al.* (73)  2014  USA  Single centre,  prospective cohort study (2b) | 58 (-, -)  922 (-, -)  Hospitalised preterm neonates receiving ABX (reason ns)  58%  Median GA 27 w (IQR 26-29 w)  **Various antibiotics** (beta-lactams, aminoglycosides, vancomycin)  ns, ns, median 14 d  Intravenous  ns  ns | Stool, Storage medium ns, temporarily at 4 °C, then -80 °C  ns  16S rRNA gene sequencing  V3-V5, 357F, 926R  454 FLX Titanium platform *(Roche)*  400-450, >500.000 reads/sample  RDP | All stools between birth and discharge (mean 61 d of life) | • ns  • ns | **Relationship** between taxa abundance and total antibiotic use, mixed model regression analysis | | • ns  • - | • Longitudinal sampling  • Large sample number³  • No control group  • No separate analysis for different ABX  • Dose of ABX ns  • Frequency of ABX ns  • No information on interval without ABX before inclusion  • DNA extraction kit ns |
|  |  |  |  |  | **Class**  Gammaproteobacteria (only in infants born after 26 w gestation) | **Class**  Clostridia (only infants born after 28 w gestation) |  |  |
| Jenke *et al.* (67)  2013  Germany  Multi-centre,  prospective cohort study (2b) | 68 (ns, ns)  248 (ns, ns)  Hospitalised preterm neonates with and without NEC receiving ABX, hospitalised preterm neonates not receiving ABX as controls  57%  ns (GA < 27 w)  ns  ns, ns, ns  ns  ns  ns | Stool, Storage medium ns, temporarily at 4°C, then -80°C  QIAamp DNA stool mini kit *(Qiagen)*  qPCR for Bifidobacteria, *Escherichia coli, C. difficile*, *Bacteroides fragilis* group, *Lactobacilli*, and total bacteria based on 16S rDNA gene sequences | 7, 14, 21, 28 d of life | • ns  • ns | **Relationship** between taxa abundance and ABX (d of life) | | • ns  • - | • Longitudinal sampling  • Analysis on species level  • Size of ABX group/controls ns  • Median/mean GA ns  • ABX ns  • Dose of ABX ns  • Frequency of ABX ns  • Duration of ABX ns  • Route of administration of ABX ns  • No information on interval without ABX before inclusion  • Short follow up⁷ |
|  |  |  |  |  | **Species**  *Clostridium difficile* (7) | No difference |  |  |
| Ferrais *et al.* (49)  2012  France  Multi-centre,  prospective cohort study (2b) | 76 (41, 35)  271 (ns, ns)  Hospitalised preterm neonates receiving ABX (reason ns), hospitalised preterm neonates not receiving ABX as controls  ns  Median GA 33 w (IQR 29-34 w)  **Various antibiotics** (amoxicillin, cefotaxime, aminoglycosides (gentamicin or amikacin), vancomycin, metronidazole, macrolides (josamycin or erythromycin), imipenem, oxacillin)  ns, ns, median of 5 d  ns  ABX/controls: 42% maternal ABX  ns | Stool, sterile tube, -80°C  Culture (quantification)  Columbia agar base, sheep blood 5%, whole milk 5%, colistin (10 mg/L), neutral red (40 mg/L) for clostridia, C. difficile supplement *(bioMerieux)*  Limit of sensitivity 10³ CFU/g stool  PCR-TTGE for Clostridia  LPW58, LPW81 | Weekly during hospitalisation (discharge at a median of 1 m of life) | • ns  • ns | **Colonisation rate ABX group vs. controls** (%) | | • ns  • No relationships between the incidence of intestinal clostridial colonisation at 1 w or at hospital discharge and ABX | • Longitudinal sampling  • Analysis on species level  • Dose of ABX ns  • Frequency of ABX ns  • Route of administration of ABX ns  • Duration of follow up ns  • No separate analysis for different ABX  • Colonisation rate only analysed for Clostridium species |
|  |  |  |  |  | **Species**  *Clostridium butyricum* (23/11) | No difference |  |  |
| Westerbeek *et al.* (104)  2012  Netherlands  Single centre,  case-control analysis (previous a randomised controlled trial) (3b) | 113 (85, 28)  ns (ns, ns)  Hospitalised preterm neonates receiving ABX (reason ns), hospitalised preterm neonates not receiving ABX as controls  ns  ns (GA < 32 w)  **ns** (‘broad-spectrum antibiotics’)  ns, ns, ns  ns  ns  ns | Stool, sterile tube, -20 °C  Fluorescent in situ hybridisation 5′-labelled with Cy3 (*Biolegio BV*), counterstaining with 4′,6-diamidino-2-phenyl- indole  Olympus AX70 epifluorescence microscope *(ns)* | 2, 7, 14 and 30 d of life | ns  ns | ns | ns | Antibiotics decreased the total bacteria count (in ABX group was 0.13 as high as in controls) | • Longitudinal sampling  • Sample size ns  • Median/mean GA ns  • ABX ns  • Dose of ABX ns  • Frequency of ABX ns  • Duration of ABX ns  • Route of administration of ABX ns  • No separate analysis for different ABX  • Only total bacterial count analysed  • No information on interval without ABX before inclusion  • Short follow up⁷ |
| Jacquot *et al.* (66)  2011  France  Single centre,  prospective cohort study (2b) | 29 (24, 5)  342 (ns, ns)  Hospitalised preterm neonates receiving ABX (reason ns), hospitalised preterm neonates not receiving ABX as controls  Median GA 27 w (IQR 27-29 w)  ns  **Various antibiotics** (penicillin G, ampicillin, cefotaxime, amikacin, vancomycin)  ns, ns, median 13 d  ns  ABX/controls: 72% maternal ABX  ns | Stool, sterile container, -20°C  MasterPure Gram Positive DNA Purification Kit *(Epicentre)*  16S rRNA gene sequencing  V2-V3, HDA1, HDA2  ns  ns, ns  ns | Every 3 d between 3 d and 2 m of life | • Richness was inversely correlated with the duration ABX (at 6 w, number of OTUs ns)  • ns | ns | ns | • ns  • - | • Longitudinal sampling  • Small cohort²  • Reason for ABX ns  • Dose of ABX ns  • Frequency of ABX ns  • Route of administration of ABX ns  • Sequencing platform, length and depth ns  • Database for taxonomic identification ns  • No abundance analysis of ABX group  • No separate analysis of different ABX |
| Savino *et al.* (88)  2011  Italy  Single centre, prospective cohort study | 26 (-,-)  ns (ns,ns)  Hospitalised children receiving ABX for pneumonia  58%  Mean age 118 d (SD ± 25 d)  **Ceftriaxon**  50 mg/kg/d, ns 5 d  Intramuscular  ns  No probiotics within 1 w | Stool  Culture for *Enterobacteriaceae*, *Lactobacilli*, Gram negative bacteria, *Clostridium difficile*  Chromogenic Coliform Agar, Rogosa Bios Agar, Gram Negative Anaerobe Selective Medium, *Clostridium difficile* Agar  Aerobic, 37°C, 24 h  Anaerobic, ns, 3 d | d 0 (before ABX), d 5 of ABX, 15 d after ABX | • ns  • ns | **Total bacterial count before vs during ABX/after ABX** | | • ns  • - | • Longitudinal sampling  • Small cohort²  • No control group  • No information on interval without ABX before inclusion |
|  |  |  |  |  | **Family**  *Enterobacteriaceae* (after ABX)  **Genus**  *Lactobacillus* (after ABX) | **Family**  *Enterobacteriaceae* (during ABX)  **Genus**  *Enterococcus* (during and after ABX)  *Lactobacillus* (during ABX) |  |  |
| Tanaka *et al.* (91)  2009  Japan  Single centre,  prospective cohort study (2b) | 23 (5 ,18)  ns (ns, ns)  Hospitalised neonates receiving ABX (reason ns), hospitalised neonates not receiving ABX as controls  Ns  Mean GA 39 w (range 36-41 w)  **Cefalexin**  200 mg/kg/d, divided into 4 doses, 4 d  Oral  ns  ns | Stool, sterile tube, refridgerator (temp. ns), then -80°C  QIAamp DNA Stool Mini-Kit *(Qiagen, Hilden, Germany)*    16S rRNA gene sequencing  V1–V3, 50-Cy5-  labelled 8UA (50-AGAGTTTGABXCCTGGCTCAG-30)  ns  ns, ns  RDP, GenBank database  qPCR | 1, 2, 3, 4, 5 d, 1, 2 m of life | • **Lower diversity** in ABX group than in controls (at 2 m of life, Shannon index ns)  • Longitudinal decrease during ABX  • ns | **Relative abundance** (time point in d/m of life) | | • ns  • - | • Longitudinal sampling  • Small cohort²  • Sample size ns  • Reason for ABX ns  • No information on interval without ABX before inclusion  • Sequencing platform, length and depth ns |
|  |  |  |  |  | **Family**  *Enterobacteriaceae* (1 m, 2 m)  **Genus**  *Enterococcus* (3 d, 1 m) | **Genus**  *Bifidobacterium* (3 d, 5 d) |  |  |
| Fouhy *et al.* (52)  2012  Ireland  Single centre,  prospective cohort Study (2b) | 18 (9,9)  ns (ns, ns)  Hospitalised neonates receiving ABX (reason ns), hospitalised neonates not receiving ABX as controls  ns  ns  **Ampicillin plus gentamicin**  ns, ns, 2-9 d  Intravenous  ns  ns | Stool, ns  QIAamp DNA stool mini kit *(Qiagen)*  16S rRNA gene sequencing  V4, one forward primer, i.e., F1 (5= AYTGGGYDTAAAGNG), and a combination of 4 reverse primers, R1 (5= TACCRGGGTHTCTAABXCC), R2 (5= TACCAGAGTABXCTAABX TC), R3 (5= CTACDSRGGTMTCTAABXC), and R4 (5= TACNVGGGTABX CTAABXCC)  Genome Sequencer FLX platform *(Roche)*  239, ns  Ribosomal Database Project (RDP) | 1 and 2 m after ABX | • **Lower richness** in ABX group compared with controls (at 1 m and 2 m, Chao 1 243/364, 2 334/490)  • Lower diversity in ABX compared with controls (at 1 m and 2 m, Shannon index 1 3.6/3.9, 2 3.8/4.6)  • No difference between ABX and controls in diversity at 1 m  • ns | **Relative abundance ABX group vs. controls** (time point in m %) | | • ns  • - | • Longitudinal sampling  • Small cohort²  • Sample size ns  • GA ns  • Sex distribution of cohort ns  • Reason for ABX ns  • Dose of ABX ns  • Frequency of ABX ns  • No information on interval without ABX before inclusion  • Storage medium ns  • Storage temperature ns  • Sequencing depth ns |
|  |  |  |  |  | **Phylum**  Proteobacteria (1 54/37;  2 44/23)  **Family**  *Enterobacteriaceae* (1 55/37; *45/24)*  *Peptostreptococcaceae* (1; 23/2)  **Genus**  *Clostridium* (1; 7/4)  *Enterococci* (ns)  *Clostridium* (2; 7/2) | **Phylum**  Actinobacteria (1; 3/24)  **Family**  *Bifidobacteriacea* (1; 3/24)  **Genus**  *Lactobacillus* (1; 1/4)  *Bifidobacterium* (1; 5/25) |  |  |
| Mangin *et al.* (78)  2010  Chile  Single centre,  prospective cohort study (2b) | 50 (31, 11)  ns (ns, ns)  Hospitalised children receiving ABX for acute bronchitis, healthy children as controls  45%  Mean 18 m (range 12-23 m)  **Amoxicillin**  50 mg/kg/d, divided into 3 doses, 7 d  Oral  ABX/controls: no ABX previous 4 w  ns | Stool, Storage medium ns, transport at 4 °C, then -30 °C  Guanidium isothiocyanate, mechanical bead beating method  PCR-TTGE for Bifidobacterium & total bacterial count based on 16S rDNA gene sequences,  Bif164f, Bif662r, Bia339f, Bia788r | 0 d (before ABX), d 7 of ABX | • ns  • ns | **Occurrence rate ABX group vs. controls** (time point in d; %) | | • ns  • **Total count**  No difference in total Bifidobacterium count between ABX group and controls | • Longitudinal sampling  • Analysis on species level  • Sample size ns  • Only *Bifidobacterium* analysed  • Short interval without ABX before inclusion⁹  • Short follow-up⁷ |
|  |  |  |  |  | - | **Species**  *Bifidobacterium*  *adolescentis* (during ABX; 0/36)  *Bifidobacterium bifidum* (during ABX; 23/5) |  |  |
| Parm *et al.* (81)  2010  Estonia  Multi-centre,  Non placebo, blinded, controlled, cluster-randomised, trial (1b) | 276  ns  Hospitalised (preterm) neonates receiving ABX for suspected early-onset neonatal sepsis, healthy neonates as controls  43%  Mean GA 31 w (SD ±5 w)  ns | Rectal swabs, transport swabs, -20 °C for < 1 w  Culture for various gram-positive and gram-negative bacteria  blood agar, MacConkey agar  Aerobic, 37°C, 24-48 h  Saboraud agar  Anaerobic, 25°C, > 1 w | Twice weekly during hospitalisation or d 60 of hospitalisation | • ns  • ns |  | | • ns  • Early empiric ABX regimen was a risk factor for the number of days colonised per 100 ICU days of all major Gram negative microorganisms (*K. pneumonia, Serratia, Acinetobacter*) | • Longitudinal sampling  • Analysis on species level  • No control group  • No placebo, only comparisons between two ABX regimens  • Sample size ns  • Short interval without (other) ABX before inclusion |
|  | **Penicillin G (plus gentamicin)** (n=137)  Penicillin G  50,000-75,000 IU/kg, divided into 2-3 doses, median 72 h  Intravenous  Gentamicin  4-5 mg/kg every 1-2 d  median 72 h  Intravenous  22% maternal ABX  ABX/controls: no other ABX during the previous 24 h |  |  |  | **Colonisation rate Penicillin vs. Ampicillin** (time point in d %; odds ratios) | |  |  |
|  |  |  |  |  | **Genus**  *Enterococcus* (6-6 ns, 2.99; 13-16 ns, 2.99)  *Acinetobacter* (10-16; 6/0, ns)  **Species**  *Staphylococcus aureus* (3-5 ns, ns)  **Difference in days colonised per 100 ICU days Penicillin vs. Ampicillin**  **Genus**  *Enterococcus* (+22.9)  *Serratia* (+3.4)  *Acinetobacter* (+2.3)  **Species**  *Staphylococcus epidermidis* (+8.5) | No difference  **Difference in days colonised per 100 ICU days Penicillin vs. Ampicillin**  **Species**  *Klebsiella pneumoniae* (-7.8)  *Staphylococcus haemolyticus* (-12.2) |  |  |
|  | **Ampicillin (plus gentamicin)** (n=139)  50-75 mg/kg/d, divided into 2-3 doses, median 64 h  Intravenous  Gentamicin  4-5 mg/kg every 1-2 d  median 64 h  Intravenous  22% maternal ABX  ABX/controls: no other ABX during the previous 24 h |  |  |  |  |  |  |  |
| Butel *et al*. (39)  2007  France  Multi-centre,  prospective cohort study (2b) | 52 (15, 37)  Mean 5 samples/infant  Hospitalised preterm neonates receiving ABX (reason ns), hospitalised preterm neonates not receiving ABX as controls  ns  Mean GA 33 w (range 30-35 w)  **ns**  ns, ns, ns  ns  ABX/controls: 48% maternal ABX  Total 40% received fermented formula containing *Bifidobacterium breve*  C50 and *Streptococcus thermophilus* (ABX, controls ns) | Stool, sterile tube, immediate freezing at -80 °C  Bead-beating method  PCR-TTGE for Bifidobacterium  S-G-Bif-164-a-S-18 and S- G-Bif-662-a-A-18  Culture for *Bifidobacterium*  trypticase-soy broth  Wilkins-Chalgren agar  Anaerobic, ns, 5 d  Limit of sensitivity 10³ CFU/g stool | Twice weekly during hospitalisation (discharge at a median 17 d of life) | • ns  • ns | No difference | No difference | • ns  • Colonisation by *Bifidobacterium* was not affected by antibiotics given to mothers or infants | • Longitudinal sampling  • Sample size ns  • Sex distribution of cohort ns  • Reason for ABX ns  • ABX ns  • Dose of ABX ns  • Frequency of ABX ns  • Duration of ABX ns  • Route of administration of ABX ns  • Temperature of anaerobic incubation ns  • Duration of follow up ns  • Only *Bifidobacterium* analysed |
| Penders *et al.* (82)  2006  Netherlands  Cross-sectional analysis (previous a community-based, prospective cohort study) (3b) | 973 (28, 945)  ns (ns, ns)  Non-hospitalised infants receiving ABX (reason ns), healthy infants as controls  48%  ns (1 m)  **ns (‘mainly Amoxicillin’)**  ns, ns, ns  Oral  ns  ns | Stool, sterile tube, then -20°C  QIAamp DNA stool mini kit *(Qiagen)*  qPCR for Bifidobacterium, *Escherichia coli, C. difficile*, Bacteroides fragilis group, lactobacilli & total bacterial count based on 16S rDNA gene sequences,  Various primers | 1 m of life | • ns  • ns | **Colonisation rate and total bacterial count ABX group vs. controls** (%; log₁₀ CFU/g stool) | | • ns  • Lower counts of Bifidobacterium were also observed after oral administration of the antimycotic miconazole | • Large cohort¹  • Cross-sectional design  • Reason for ABX ns  • ABX ns  • Dose of ABX ns  • Frequency of ABX ns  • Duration of ABX ns  • No information on interval without ABX before inclusion  • Short follow-up⁷  • No separate analysis for different ABX |
|  |  |  |  |  | - | **Genus**  *Bifidobacterium* (98/100; 10.3/10.7)  *Bacteroides* (82/82; 6.4/9.3) |  |  |
| Bonnemaison *et al.* (34)  2003  France  Single centre,  prospective cohort study (2b) | 30 (20, 10)  ns (ns, ns)  Hospitalised preterm and term neonates receiving ABX for suspected maternofoetal infection, hospitalised preterm and term neonates not receiving ABX as controls  ns  ABX group mean GA 33 w (SD ±5 w), controls mean GA 33 w (SD ±2 w)  ns | Stool/rectal swab, sterile tubes, +4°C, then –20°C  Cultures for *Klebsiella, Escherichia coli, Proteus, Enterobacter, Citrobacter, Enterococcus, Staphylococcus, Pseudomonas aeruginosa*  Trypticase-soy agar, Drigalski agar, Chapman agar, Cocosel agar, Sabouraud’s agar (*bioMérieux*), Mueller-Hinton  Anaerobic, 37°C, 48 h  ns | 0 d (before ABX), 3, 7, 10 d of life | • ns  • ns |  | | • ns  • - | • Longitudinal sampling  • Small cohort²  • Sex distribution of cohort ns  • Dose of ABX ns  • Frequency of ABX ns  • Short follow-up⁷  • Limit of sensitivity of culture ns  • No p-values provided |
|  | **Amoxicillin plus netilmicin** (n=10)  ns, ns, 48-72 h  ns  ABX/controls: 40 % maternal ABX |  |  |  | **Colonisation rate ABX groups vs. controls** (time point of life in d %, no p-values provided) | |  |  |
|  |  |  |  |  | **Family**  *Enterobacteriaceae* (3 60/50; 7 90/60; 10; 100/60)  **Genus**  *Enterococcus* (7 50/10) | **Genus**  *Staphylococcus* (3 50/90;  7 60/100; 10 60/90)  *Enterococcus* (3 0/10) |  |  |
|  | **Amoxicillin plus cefotaxime plus netilmicin** (n= 10**)**  ns, ns, 48-72 h  ns  ABX/controls: 40 % maternal ABX |  |  |  | **Genus**  *Enterococcus* (7 20/10) | **Family**  *Enterobacteriaceae* (3 20/50;7 20/60; 10 40/60)  **Genus**  *Staphylococcus* (3 30/90)  *Enterococcus* (10 20/50) |  |  |
| Hall *et al.* (61)  1990  England  Single centre,  prospective cohort study (2b) | 42 (30, 12)  ns (ns, ns)  Hospitalised preterm neonates receiving ABX (reason ns), hospitalised preterm neonates not receiving ABX as controls  ns  Median GA 32 w (range 25-33 w)  ns  ns, ns, ns  Intravenous  ABX/controls: no ABX for > 48h  ns | Stool, bottle with buffered salt solution Cary Blair medium (*Oxoid*), fridge or cool environment  Culture for *Lactobacillus, Bifidobacterium,* Coliforms  McConkey´s agar, acetate agar, medium based on deMan *et al.*  ns | 10, 30 d of life | • ns  • ns | **Colonisation rate and total bacterial count ABX group vs. controls** (time point in d %, log₁₀ CFU/g stool) | | • ns  • - | • Longitudinal sampling  • Small cohort²  • Sample size ns  • Reason for ABX ns  • Information on ABX ns  • Reason for ABX ns  • ABX ns  • Dose of ABX ns  • Frequency of ABX ns  • Duration of ABX ns  • Limit of sensitivity of culture ns  • No separate analysis for different ABX  • Short interval without ABX before inclusion  • Short follow-up⁷ |
|  |  |  |  |  | No difference | **Genus**  *Lactobacillus* (10 7/33, 8.2/7.7) |  |  |
| Holton et al. (63)  1989  UK  Single centre, single-blind, non-placebo-controlled, randmoised controlled trial (1b) | 135 (95,40)  416 (ns,ns)  Hospitalised neonates with suspected sepsis randomised to two different ABX, neonates not receiving ABX as controls  39%  ns  ns | Stool, ns, refrigerator  Culture for Clostridium difficile  Blood agar  Anaerobic, 37°C, 48 h | Weekly during hospitalization | • ns  • ns | **Colonisation rate ABX group vs. controls** (% ABX/controls) | | • ns  • - | • -  • Age distribution ns  • Route of administration ns  • No information on interval without ABX before inclusion |
|  | **Cefotaxime (n=48)**  50 mg/kg/d, divided into 2 doses, mean 4 d  ns  ns |  |  |  |  | **Species**  *Clostridum difficile* (13/40) |  |  |
|  | **Penicillin plus netilmicin (n=47)**  60mg/kg/d, divided into 2 doses, mean 4 d  Netilmicin  4 mg/kg/d divided into 2 doses  ns  ns |  |  |  |  | **Species**  *Clostridum difficile* (21/40) |  |  |
| Tullus *et al.* (93)  1988  Sweden  Multi-centre,  cross-sectional study (3b) | 953 (348, 605)  ns (ns, ns)  Hospitalised neonates receiving ABX (reason ns), hospitalised neonates not receiving ABX as controls  ns  ABX group mean GA 32 w (range 25-33 w), controls mean GA 40 w (range 38-42 w)  ns | Rectal swab, cryopreservative medium, -80°C  Culture for *Klebsiella/Enterobacter, Pseudomonas, Proteus, Citrobacter, Acinetobacter, Escherichia coli*  Endo agar, Colonisation factor Antigen agar  ns, ns, ns  ns | Day of discharge (ns) | • ns  • ns |  | |  | • Large cohort¹  • Analysis on species level  • Cross-sectional design  • Sample size ns  • Sex distribution of cohort ns  • Reason for ABX ns  • Dose of ABX ns  • Frequency of ABX ns  • Duration of ABX ns  • Route of administration of ABX ns  • Reason for ABX not specified  • Temperature of incubation ns  • Duration of incubation ns  • Limit of sensitivity of the culture ns  • Timing of sampling ns  • No information on interval without ABX before inclusion  • Duration of follow up ns |
|  | **Ampicillin (plus gentamicin)** (n=197**)**  ns, ns, ns  ns  ns |  |  |  | **Colonisation rate ABX group vs. controls** (%) | | • Resistance of *Escherichia coli, Klebsiella, Enterobacter* |  |
|  |  |  |  |  | **Genus**  *Klebsiella/Enterobacter* (48/38) | **Species**  *Escherichia coli* (9/17) |  |  |
|  | **Cefuroxime** (n= 95**)**  ns, ns, ns  ns  ns |  |  |  | **Genus**  *Citrobacter* (ns)  *Pseudomonas* (ns)  *Proteus* (ns)  *Acinetobacter* (ns) | **Species**  *Escherichia coli* (7/17) | • No resistance of *Escherichia coli* |  |
| Bennet *et al.* (32)  1987  Sweden  Single centre,  prospective cohort study (2b) | 166 (106, 60)¹  ns (ns, ns)  Hospitalised preterm and term neonates receiving ABX various infections (suspected sepsis, post-surgery, dermatological infection), hospitalised preterm and term neonates not receiving ABX as controls  ns  Median GA 34 w (range 28-42 w)  **Various antibiotics**  **Benzylpenicillin**  100 mg/kg/d, ns, ns  ns  **Cloxacillin**  100 mg/kg/d, ns, ns  Intravenous  ns  **Flucloxacillin**  50 mg/kg/d, ns, ns  Oral  ns  **Ampicillin**  200 mg/kg/d, ns, ns  Intravenous  ns  **Cefuroxime**  150 mg/kg/d, ns, ns  Intravenous  ns  **Cefoxitin**  100 mg/kg/d, ns, ns  Intravenous  ns  **Gentamicin**  5-7.5 mg/kg/d, ns, ns  Intravenous  ns  ns | Stool, non-sterile plastic containers, anaerobic Storage medium ns, ns  Culture for *Clostridium, Klebsiella/Enterobacter, Escherichia coli, S. faecalis, S. epidermidis*,  Blood agar  Aerobic/anaerobic, ns, ns  ns | d 4 of ABX, then every 2 w | • ns  • ns | **Colonisation ABX group vs. controls** (number of isolates of resp. bacteria) | | • ns  • - | • Longitudinal sampling  • Sample size ns  • Sex distribution of cohort ns  • Overlapping cohort with other Bennet-study  • No separate analysis for different ABX  • Frequency of ABX ns  • Duration of ABX ns  • No information on interval without ABX before inclusion  • Anaerobic Storage medium ns  • Storage temperature ns  • Temperature of incubation ns  • Duration of incubation ns |
|  |  |  |  |  | - | **Genus**  *Bacteroides* (16/51)  *Bifidobacterium* (43/60)  *Lactobacillus* (21/29) |  |  |
| Bennet *et al.* (31)  1986  Sweden  Single centre,  prospective cohort study (2b) | 164 (104, 60)¹  ns (ns, ns)  Hospitalised preterm and term neonates receiving ABX various infections (suspected sepsis, post-surgery, dermatological infection); hospitalised preterm and term neonates not receiving ABX as controls  ns  ns (GA range 30-39 w)  **Various antibiotics**  **Benzylpenicillin**  100 mg/kg/d, ns, ns  ns  **Cloxacillin**  100 mg/kg/d, ns, ns  Intravenous  ns  **Flucloxacillin**  50 mg/kg/d, ns, ns  Oral  ns  **Ampicillin**  200 mg/kg/d, ns, ns  Intravenous  ns  **Cefuroxime**  150 mg/kg/d, ns, ns  Intravenous  ns  **Cefoxitin**  100 mg/kg/d, ns, ns  Intravenous  ns  **Gentamicin**  5-7.5 mg/kg/d, ns  Intravenous  ns  ns | Stool, anaerobic Storage medium, ns  Culture for *Clostridium, Klebsiella/Enterobacter, Escherichia coli, S. faecalis, S. epidermidis*, anaerobic bacteria  blood agar  Aerobic/anaerobic, ns, ns  ns | d 4 of ABX, then every 2 w | • ns  • ns | **Colonisation rate ABX group vs. controls** (%) | | • ns  • - | • Longitudinal sampling  • Analysis on species level  • Sample size ns  • Sex distribution of cohort ns  overlapping cohort with other Bennet-study  • Frequency of ABX ns  • Duration of ABX ns  • No information on interval without ABX before inclusion  • No separate analysis for different ABX  • Storage temperature ns  • Temperature of incubation ns  • Duration of incubation ns |
|  |  |  |  |  | **Genus**  *Klebsiella/Enterobacter* (ns) | Anaerobic bacteria (ns)  **Species**  *Escherichia coli* (28/54)  *Enterococcus faecalis* (except in cephalosporin groups, ns) |  |  |
| Butel *et al.* (38)  1986  France  ns,  prospective cohort study (2b) | 12 (-, -)  ns (-, -)  Hospitalised infants receiving ABX for respiratory infections  ns  ns (range birth-1 y)  **Erythromycin**  75 mg/kg/d, divided into 2-3 doses, 7-10 d  Oral  ABX/controls: no ABX within 15 d  ns | Stool, ns, -80°C  Culture for *Enterobacter, Staphylococcus, Pseudomonas aeruginosa, Streptococcus, Fusobacterium, Bacteroides, Lactobacillus*  Trypticase-soy broth  Drigalski agar, Chapman no. 110 agar, cetrimide agar  Aerobic, 37°C, 48 h  Columbia agar with nalidixic acid, Wilkins-Chalgren agar, Rogosa agar  Anaerobic, 37°C, 6 d  ns | 0 d (before ABX)  every 2 d during ABX | • ns  • ns | **Total bacterial count before vs. during ABX** (change in log₁₀ CFU/g stool, no p-values provided) | | • ns  • No effect on *streptococci* and anaerobes | • Longitudinal sampling  • Small cohort²  • Number of samples ns  • Sex distribution of cohort ns  • Short follow-up⁷  • No molecular diagnostics  • No analysis on species level  • p-values ns  • Short interval without ABX before inclusion  • Storage medium ns  • Limit of sensitivity of the culture ns |
|  |  |  |  |  | ns | **Genus**  *Enterobacter* (10³-10⁵-fold) |  |  |
| Welkon *et al.* (96)  1986  USA  ns,  pre-post intervention study (4) | 21 (-, -)  ns (-, -)  Hospitalised children receiving ABX for various infections (ns)  ns  Mean 3.7 y (ns)  **Imipenem-cilastatin**  <3 y 60 mg/kg/d, >3 y 100 mg/kg/d, ns, mean 4 d  Intravenous  ABX/controls: no ABX previous 14 d  ns | Stool/rectal swab, anaerobic salt broth, agar  Culture for *Escherichia coli, Klebsiella, Enterobacter, Proteus, Pseudomonas, Lactobacillus, Enterococcus,* other streptococci*, S. aureus, Bacteroides,* other Gram negative Bacilli*, Clostridium. difficile,* other clostridium  Trypticase-soy sheep blood agar, enterococcoceal agar, MacConkey agar, cetrimide agar  Aerobic, ns, ns  Anaerobic GasPak jars, Schaedler KV agar, lactobacillus agar, cycloserine-cefoxitin-fructose agar, imipenem agar  Anaerobic, 38°C, 48 h  Limit of sensitivity 10³ CFU/g stool | 0 d (before ABX)  every 2-3 d, end of ABX | • ns  • ns | **Total bacterial count before vs. during ABX** (log₁₀ CFU/g stool, no p-values provided) | | • No resistance to imipenem  • - | • Longitudinal sampling  • Analysis on species level  • No control group  • Sex distribution of cohort ns  • Frequency of ABX ns  • No molecular diagnostics  • p-values ns  • Short interval without ABX before inclusion⁹  • Short follow-up⁷  • Temperature of aerobic incubation ns  • Duration of aerobic incubation ns |
|  |  |  |  |  | **Genus**  *Enterococcu*s (6.9/10.2)  *Proteus* (4.6/7.4)  *Pseudomonas* (6.2/7.8)  *Enterobacter* (6.0/7.9) | **Genus**  *Klebsiella* (5.6/5.2)  *Lactobacillus* (6.5/5.8)  Other streptococci (6.8/5.2)  **Species**  *Escherichia coli* (8.8/ 7.7)  *Staphylococcus aureus* (6.1/4.1)  *Bacteroides fragilis* (9.3/8.9) |  |  |
| Borderon *et al.* (105)  1986  France  ns,  pre-post intervention study (4) | 18 (-, -)  Ns (-, -)  Children hospitalised with infections  ns  ns (range 20 d-15 y)  **Imipenem-cilastatin**  60-100 mg/kg/day, ns, ns  Intravenous  ABX/controls: 44% had other ABX before (switch to imipenem-cilastin)  ns | Stool, ns  Culture for *Enterobacter, Pseudomonas, S. aureus*, group D streptococci  Drigalski agar, mannitol-salt agar, aesculin agar  Aerobic, 37°C, 24 h  Limit of sensitivity 10² CFU/g stool | 0 d (before ABX), 3-7 and 7-14 d of ABX  2-3 d after ABX | • ns  • ns | No effect on the count of Enterobacteria | | • In 1 child *Pseudomonas aeruginosa* was resistant to Imipenem  • - | • Longitudinal sampling  • Small cohort²  • No control group  • Sex distribution of cohort ns  • Frequency of ABX ns  • Duration of ABX ns  • Short follow-up⁷  • Storage medium ns  • Storage temperature ns  • No molecular diagnostics  • No analysis on species level  • Different ABX before inclusion |
| Sakata *et al.* (87)  1986  Japan  ns,  prospective cohort Study (2b) | 69 (54, 15)  ns (ns, ns)  Hospitalised children receiving ABX for various infections (respiratory, urinary tract), children hospitalised for endocrinologic/developmental investigations as controls  ns  Mean 3.2-8.4 y (respective ABX groups)  ns | Stool, sterile tube, 4°C  Culture for *Eubacterium, Peptococcaceae, Veillonella, Megasphaerae, Bacillus, Pseudomonas, Acteroidaceae, Bifidobacterium, Clostridium, Enterobacteriaceae, Lactobacillus, Staphylococcus, Streptococcus*  Trypticase soy blood agar, DHL agar, TABXAC agar, PEES agar Potato dextrose agar, Nalidixic acid-cetrimide agar  Aerobic, ns, ns  Medium 10, Glucose-blood-liver agar, modified Eggert-Gagnon alkaline blood agar, Bismuth sulfite agar, Neomycin Nagler agar, Modified Veillonella agar, Modified lactobacillus selection agar  Anaerobic, ns, ns  Limit of sensitivity 10² CFU/g stool | 0 d (before ABX)  4-6 of ABX  and 3-6 d after ABX | • ns  • ns |  | | • ns  • - | • Longitudinal sampling  • Sample numbers ns  • No information on interval without ABX before inclusion  • Frequency of ABX ns  • Duration of ABX ns  • Short follow-up⁷  • Temperature of aerobic incubation ns  • Duration of aerobic incubation ns  • No molecular diagnostics  • No analysis on species level |
|  | **Ampicillin** (6)  40-50 mg/kg/d, ns, ns  Oral  ns |  |  |  | **Total bacterial count before vs. during ABX** (log₁₀ CFU/g stool) | |  |  |
|  |  |  |  |  | No difference | **Genus**  *Bifidobacterium* (10/7)  *Streptococcus* (9/7)  *Lactobacillus* (7/6) |  |  |
|  | **Penicillin V** (5)  50.000 U/kg/d, ns, ns  Oral  ns |  |  |  |  | **Genus**  *Bifidobacterium* (10.5/8.5)  *Streptococcu*s (9/6)  *Lactobacillus* (7.5/5.5) |  |  |
|  | **Cefaclor** (6)  40-50 mg/kg/d, ns, ns  Oral  ns |  |  |  |  | **Family**  *Enterobacteriaceae* (9/5.5)  **Genus**  *Bifidobacterium* (10.5/9) |  |  |
|  | **Erythromycin** (6)  40-50 mg/kg/d, ns, ns  Oral  ns |  |  |  |  | **Family**  *Enterobacteriaceae* (9/6)  **Genus**  *Bifidobacterium* (10.5/9) |  |  |
|  | **Gentamicin** (3)  60 mg/kg/d, ns, ns  Oral  ns |  |  |  |  | **Family**  *Enterobacteriaceae* (7/1)  **Genus**  *Streptococcus* (8/1)  *Clostridium* (6/1)  *Lactobacillus* (6/1) |  |  |
|  | **Ampicillin** (6)  150-200 mg/kg/d, ns, ns  Intravenous  ns |  |  |  |  |  |  |  |
|  |  |  |  |  |  | **Genus**  *Bifidobacterium* (10.5/8)  *Streptococcu*s (8/5.5)  *Lactobacillus* (6.5/6) |  |  |
|  | **Methicillin** (8)  150-200 mg/kg/d, ns, ns  Intravenous  ns |  |  |  |  | **Genus**  *Bifidobacterium* (10.5/7)  *Streptococcu*s (9/8)  *Lactobacillus* (6/5.5) |  |  |
|  | **Cefpiramide** (7)  60 mg/kg/d, ns, ns  Intravenous  ns |  |  |  |  | **Family**  *Enterobacteriaceae* (9/4)  *Bacteroidaceae* (10.5/9.5)    **Genus**  *Bifidobacterium* (10/6)  *Lactobacillus* (4/1.5)  *Staphylococcus* (3/1.5) |  |  |
|  | **Ceftazidime** (7)  80 mg/kg/d, ns, ns  Intravenous  ns |  |  |  |  | **Family**  E*nterobacteriaceae* (9/3.5)  **Genus**  *Bifidobacterium* (10.5/8.5)  *Lactobacillus* (6.5/5.5) |  |  |
| Guggenbichler *et al.* (60)  1984  Austria  ns,  pre-post intervention study (4) | 20 (-, -)  ns (-, -)  Hospitalised children receiving ABX for various infections (sepsis, meningitis, gastrointestinal, urinary tract infection)  ns  ns (range 2-18 m)  **Cefoperazone, ceftriaxone, cefotaxime**  ns, ns, ns  Intravenous  ABX/controls: no ABX previous 2 w  ns | Stool, non-sterile container with sterile water, agar  Culture (investigated bacteria ns)  Blood agar base with 5% sheep blood, McConkey agar, Pfizer selective enterococcus agar, Sabouraud agar  Aerobic, ns, 24 h  ns | 0 d (before ABX), every 2 d until 1 w after ABX | • ns  • ns | **log10 CFU/g stool** (no p-values provided) | | • Resistances to cefoperazone after ABX: *Klebsiella/Enterobacter, Citrobacter, Serratia, Escherichia coli*  • Resistances to ceftriaxone after ABX: *Klebsiella/Enterobacter, Citrobacter, Serratia, Escherichia coli* and *Pseudomonas aeruginosa*  • No resistances to cefotaxime | • Longitudinal sampling  • No control group  • Small cohort²  • Sex distribution of cohort ns  • Dose of ABX ns  • Frequency of ABX ns  • Duration of ABX ns  • Short follow-up⁷  • Temperature of incubation ns  • Limit of sensitivity of the culture ns  • No analysis on species level  • No molecular diagnostics  • p-values ns  • Short interval without ABX before inclusion |
|  |  |  |  |  | **Genus**  *Klebsiella/Enterobacter* (ns)  **Species**  *Pseudomonas aeruginosa* (ns)  *Citrobacter freundii* (ns)  *Serratia marcescens* (ns)  *Escherichia coli* (ns) | ? |  |  |
| Borderon *et al.* (35)  1985  France  ns,  prospective cohort study (2b) | 27 (15, 12)  ns (ns, ns)  Hospitalised children receiving ABX for various infections (respiratory, urinary tract), healthy children as controls  ns  ns (range 1 m-13 y)  **Amoxicillin/clavulanic acid**  40-50 mg/kg/d, divided into 3-4 doses, 6-21 days  Oral  ABX: received amoxicillin or trimethoprim/sulfamethoxazole before  Controls no previous ABX  ns | Stool, sterile tube, 4°C for max. 24 h  Culture for *Enterobacteriaceae, Pseudomonas,* Group D streptococcus, *Staphylococcus*  Chapman agar, Sabouraud’s agar, Mueller-Hinton, Esculin acid agar  ns, ns, ns  Limit of sensitivity 10² CFU/g stool | 0 d (before ABX), 7 d then weekly, 2-3 d after ABX (none had sample taken before AND after ABX) | • ns  • ns | **Colonization rate before vs. after ABX** (no p-values provided) | | • ns  • - | • Longitudinal sampling  • Small cohort²  • Sample numbers ns  • Sex distribution of cohort ns  • Short follow-up⁷  • Temperature of incubation ns  • Duration of incubation ns  • No comparison between ABX group and controls performed, no p-values provided  • Different ABX before inclusion |
|  |  |  |  |  | **Family**  Enterobacteriaceae | **Species**  Group D streptococcus |  |  |
| Lambert-Zechovsky N *et al.* (106)  1984  France  ns,  pre-post intervention study (4) | 16 (-, -)  ns (-, -)  Hospitalised infants receiving ABX various infections (sepsis, respiratory, gastrointestinal)  ns  ns (<3 y n =9, >3 y n=7)  **Cefoperazone** **plus gentamicin, tobramycin or amikacin**  100 mg/kg/d, ns, ns  Intravenous  ns  ns | Stool, ns  Culture for *Escherichia coli, Klebsiella, Enterobacteria, Pseudomonas, Staphylococcus,* Group D streptococcus, anaerobic bacteria  Enterosel agar, Chapman agar, Man, Rogosa, Sharpe agar, Drigalski agar, Hektoen Enteric agar, Cetrimide agar  Aerobic, 30/37°C, 24h  Anaerobic, 37°C, 48/96 h  Limit of sensitivity 10² CFU/g stool | 0 d (before ABX), d 4 and 7 of ABX, 4 d after ABX | • ns  • ns | **Colonisation rate** (% of children with a change before vs. after ABX, no p-values provided) | | • ns  • Reduction of total bacterial count (below 10² CFU/g stool) in 44%  • Normalisation 5-10 d after stop of cefoperazone | • Longitudinal sampling  • Analysis on species level  • Small cohort²  • No control group  • Sample numbers ns  • Sex distribution of cohort ns  • Frequency of ABX ns  • Duration of ABX ns  • No information on interval without ABX before inclusion  • Short follow-up⁷  • Storage medium ns  • Storage temperature ns  • No molecular diagnostics  • p-values ns |
|  |  |  |  |  | - | **Genus**  *Staphylococcus* (66)  **Species**  *Escherichia coli* (81)  Group D streptococcus (57) |  |  |
| Lambert-Zechovsky N *et al.* (107)  1984  France  ns,  pre-post intervention study (4) | 11 (-, -)  ns (-, -)  Hospitalised children (ICU or nephrology) receiving ABX for various infections (respiratory gastrointestinal, urinary tract infection)  ns  ns (<2 y n=2, > 6 n=9)  **Amoxicillin-clavulanic acid**  82.5 mg/kg/d, divided into 3 doses, ns  Oral/intravenous  ns  ns | Stool, ns  Culture for *Escherichia coli, Klebsiella, Enterobacteria, Pseudomonas, Staphylococcus,* Group D streptococcus, anaerobic bacteria  Enterosel Agar, Chapman agar, Man, Rogosa, Sharpe agar, Drigalski agar, Hektoen Enteric agar, Cetrimide agar  Aerobic, 30/37°C, 24h  Anaerobic, 37°C, 48/96 h  Limit of sensitivity 10² CFU/g stool | 0 d (before ABX)  d 4 of ABX | • ns  • ns | **Total bacterial count before vs. during ABX** (log₁₀ CFU/g stool, no p-values provided) | | • ns  • Increase in abundance of *C. albicans* (before vs. after ABX) | • Longitudinal sampling  • Analysis on species level  • Small cohort²  • No control group  • Sample numbers ns  • Sex distribution of cohort ns  • Duration of ABX ns  • No information on interval without ABX before inclusion  • Short follow-up⁷  • Storage medium ns  • Storage temperature ns  • No molecular diagnostics  • p-values ns |
|  |  |  |  |  | **Genus**  *Klebsiella* (ns)  **Species**  *Escherichia coli* (ns) | - |  |  |
| Fujita *et al.* (53)  1984  Japan  Prospective cohort study (2b) | 19 (9, 10)  ns (ns, ns)  Hospitalised children receiving ABX for various infections or as prophylaxis, children hospitalised for endocrinological investigations as controls  ns  Mean 5.4 y (range 1–11 y)  **Trimethoprim/sulfamethoxazole**  115-200/577-1000 mg/m²/d, ns, 4-6 d  ns  ns  ns | Stool, transport medium with CO_2_, 4°C until culture  Culture for *Bifidobacterium, Eubacterium, Bacteroides, Clostridium, Veillonella, Lactobacillus, Enterobacteriaceae, Streptococcus, Staphylococcus, Pseudomonas,* anaerobic bacteria  Trypticase soy blood agar, Deoxycholate Hydrogen sulfide Lactose agar, TATAC agar (*Streptococcus*), PEES agar (*Staphyloccus*), potato dextrose agar, Nalidixic Acid Cetrimide agar  Aerobic, 37°C, 48 h  Medium 10, ‘BL agar’ (Anaerobes), ‘EG agar’ (Anaerobes), Bifidobacterium selective agar, neomycin-brilliant green-taurocholate-blood agar, Neomycin Nagler agar, Modified Veillonella selective agar, Modified Lactobacillus selective agar  Anaerobic, 37°C, 3/5 d  Limit of sensitivity 10² CFU/g stool | 0 d (before ABX)  d 4,5,6 of ABX, 3,4,5,6 d after ABX | • **No difference in bacterial counts** between ABX group and controls  • No difference in bacterial counts before vs. after ABX  • ns | **Total bacterial count before ABX vs. during ABX** (log₁₀ CFU/g stool) | | • ns  • Abundance of *Enterobacteriaceae* and *Veillonella* returned to normal after stop of ABX | • Longitudinal sampling  • Small cohort²  • Sample numbers ns  • Sex distribution of cohort ns  • Frequency of ABX ns  • Route of administration of ABX ns  • No information on interval without ABX before inclusion  • Short follow-up⁷  • No molecular diagnostics  • No analysis on species level |
|  |  |  |  |  | - | **Genus**  *Enterobacter* (8.0/4.2)  *Veillonella* (6.7/3.5) |  |  |
| Sunakawa *et al.* (90)  1984  Japan  Single centre, prospective cohort study (2b) | ns  ns  Hospitalised children receiving ABX (reason ns)  ns  ns (< 2y)  ns | Stool, sterile container, immediate processing  Culture for *Escherichia coli, Staphylococcus aureus, Streptococcus faecalis, Bateroides, Clostridium*  BTB medium, blood agar medium, PEA-azide medium, mannitol-salt mediuman,, Sabouraud medium  Aerobic, 37°C, 48 h | Before, during, after ABX | • ns  • ns | **Bacterial count during ABX** (no p-values provided) | | • ns  •**Total bacterial counts**  Changes in bacterial counts normalized after the discontinuation of ABX | • -  • Cohort size ns,  • Sample size ns  • No control group  • Sex distribution ns  • Reason for ABX ns  • No information on interval without ABX before inclusion |
|  | **Ampicillin** (n=1)  100 mg/kg/d, divided into 3 doses, ns  Intravenous  ns |  |  |  |  | **Genus**  *Clostridium*  **Species**  *Streptococcus faecalis*  *Staphylococcus aureus* |  |  |
|  | **Various cephalosporins** (cephalothin, cephem, cefmenoxime, ceftazidime, n=ns)  100 mg/kg/d, divided into 3 doses, ns  Intravenous  ns |  |  |  |  | **Genus**  *Staphyloccus*  *Klebsiella*  *Clostridium*  **Species**  *Escherichia coli* |  |  |
| Blakey *et al.* (33)  1982  Australia  Single centre,  prospective cohort study (2b) | 28 (20/8, 8/20)  143 (ns, ns)  Hospitalised preterm and term neonates receiving ABX (reason ns), hospitalised preterm and term neonates not receiving ABX as controls  75%  Mean GA 30 w (range 25-36 w)  **Various antibiotics** (Penicillin, penicillin plus gentamicin, others)  ns, ns, ns  Intravenous  ns  ns | Stool, ns  Cooked-meat broth with Schaedler broth  Horse-blood agar, MacConkey agar, mannitol salt agar, Columbia agar  Aerobic/anaerobic, 37°C, 24-48 h  Electronmicroscopy (viruses) | d 1 of hospitalisation, then every 2 d during the first 3 w of life | • ns  • ns | **Colonisation rate ABX group vs. controls** (time point in d of life % children of ABX group/controls) | | • ns  • **Colonisation**  Bacteroides the most prevalent on d 4 of life, then declined after d 12, *Escherichia coli* and *Klebsiella* common after d 4 and persisted, low prevalence of *Clostridium* | • Longitudinal sampling  • Analysis on species level  • Small cohort²  • 12 children switched into the control group (day 12)  • Reason for ABX ns  • Dose of ABX ns  • Frequency of ABX ns  • Duration of ABX ns  • No information on interval without ABX before inclusion  • Short follow-up⁷  • Storage conditions ns  • Only investigated colonisation rates, not abundance  • No molecular diagnostics  • p-values ns |
|  |  |  |  |  | - | **Genus**  *Lactobacillus* (< 11 0/25; > 12 0/15)  **Species**  *Clostridium butyricum* (< 11 5/50)  *Clostridium perfringens* (< 11 10/50)  *Clostridium difficile* (< 11 0/25) |  |  |
| Grylack et al. (59)  1982  USA  Single centre, double-blind, non-placebo-controlled, randomised trial (1b) | 100 (-,-)  980 (-,-)  Hospitalised preterm neonates with suspected NEC randomised to two different ABX  ns  Mean GA 33 w (SD ± 3.75 w)  **Gentamicin** (n=50)  10 mg/kg/d, divided into 4 doses, 3 w  Oral  ns  **Colistin** (n=50)  4 mg/kg/d, divided into 4 doses, 3 w  Oral  ns  ns | Stool  Culture for *Staphylococcus aureus, Staphylococcus epidermidis, Escherischia coli, Enterobacter, Proteus, Klebsiella, Citrobacter, Acinetobacter, Group B Streptococcus, Serratia*  Sheep blood agar, MacConkey agar, desoxycholate agar  Aerobic, ns, ns  ns | 1, 11, 21 d of life | • ns  • ns | **Most dominant bacterial organism (in both ABX groups)** | | • 36% of all organisms identified in the stools of the gentamicin group were resistant to gentamicin  • 20% of all organisms identified in the stools of the colistin group were resistant to colistin  • 17% of the gram-negative species were resistant to colistin  • 9% of the gram-negative species were resistant to gentamicin  • The number of resistant organisms in the stools of either group did not increase from day 1 to day 21 of the study period  • **Total bacterial growth**  Bacterial growth of any species was more common for the colistin group at 1 d | • Longitudinal sampling  • Large sample size³  • No control group  • No information on interval without ABX/maternal ABX before inclusion for ABX group |
|  |  |  |  |  | **Species**  *Staphylococcus epidermidis*  Gram-negative bacteria (increase between 1- 21 d of life) |  |  |  |
| Bingen *et al.* (110)  1982  France  Single centre, prospective cohort study (2b) | 149 (85,64)  ns (ns,ns)  Hospitalised children receiving ABX for sepsis or urinary tract infections, hospitalised children not receiving ABX as controls  ns  ns  **Ampicillin plus gentamicin or tobramycin**  Ampicillin 100-300 mg/kg/d, ns,ns  Oral  Gentamicin 3mg/kg(d, ns, ns  Intramuscular  Tobramycine 3 mg/kg/d, ns, ns  Intramuscular  ns  ns | Stool, ns  Culture for *Staphylococcus, Enterococcus, Lactobacillus, Enterbacteria, Salmonella, Shigella, Pseudomonas, Streptococcus*  Chapman agaer, Man–Rogosa–Sharpe agar, Drigalski agar, Hektoen agar, gelatin salt agar,  Aerobic, 30/37°C, 24/48h  Anaerobic, 37°C, 48/96h  ns | Every 3 d  (start and end point ns) | • ns  • ns | **Total bacterial count before ABX vs. during ABX** (no p-values provided) | | • ns  • - | • Longitudinal sampling  • Analysis on species level  • Sample number ns  • Sex distribution of cohort ns  • Frequency of ABX ns  • Duration of ABX ns  • No information on interval without ABX before inclusion  • Short follow-up⁷  • Storage conditions ns  • No comparison between ABX group and controls  • p-values ns |
|  |  |  |  |  | **Genus**  *Enterobacter*  **Species**  *Klebsiella pneumoniae*  *Escherichia coli* |  |  |  |
| Lambert-Zechovsky *et al.* (108)  1981  France  Single centre,  Cross-sectional analysis (3b) | 62 (21, 41)  ns (ns, ns)  Hospitalised children receiving ABX (reason ns), hospitalised children not receiving ABX as controls  ns  ns (<3 m 28, >3 m 34)  **Colistin plus gentamicin**  50-100,000 IU/kg/d, ns, ns  Oral/intravenous, gentamicin intramuscular  ns  ns | Stool, ns  Culture for *Escherichia coli, Klebsiella, Enterobacteria, Pseudomonas, Staphylococcus,* Group D streptococcus*,* anaerobic bacteria  Enterosel agar, Chapman agar, Man, Rogosa, Sharpe agar, Drigalski agar, Hektoen Enteric agar, Cetrimide agar  Aerobic, 30/37°C, 24h  Anaerobic, 37°C, 48/96 h  Limit of sensitivity 10² CFU/g stool | Every 3 d during hospitalisation | • ns  • ns | **Total bacterial count before ABX vs. during ABX** (no p-values provided) | | • ns  • - | • Longitudinal sampling  • Analysis on species level  • Sample number ns  • Sex distribution of cohort ns  • Reason for ABX ns  • Frequency of ABX ns  • Duration of ABX ns  • No information on interval without ABX before inclusion  • Short follow-up⁷  • Storage conditions ns  • No comparison between ABX group and controls  • No molecular diagnostics |
|  |  |  |  |  | **Genus**  *Proteus*  *Staphylococcus*  **Species**  *Staphylococcus aureus* | **Genus**  *Enterobacter*  *Klebsiella*  **Species**  *Escherichia coli* (only in children receiving colistin plus gentamicin) |  |  |
| Lambert-Zechovsky *et al.* (109)  1981  France  ns, prospective cohort study (2b) | 51 (10, 41)  ns (ns, ns)  ns  ns  ns  **Cefotaxime plus gentamicin**  Cefotaxime 50-100 mg/kg/d divided into 2 doses, ns  Intravenous  Gentamicin  ns, ns, ns  ns  Some children previously had ampicillin plus gentamicin (ns)  ns | Stool, ns  Culture for *Escherichia coli, Klebsiella, Enterobacteria, Pseudomonas, Staphylococcus,* Group D streptococcus*,* anaerobic bacteria  Enterosel agar, Chapman agar, Man, Rogosa, Sharpe agar, Drigalski agar, Hektoen Enteric agar, Cetrimide agar  Aerobic, 30/37°C, 24h  Anaerobic, 37°C, 48/96 h  Limit of sensitivity 10² CFU/g stool | Every 3 d  (start and end point ns) | • ns  • ns | **Total bacterial count before ABX vs. during ABX** (log₁₀ CFU/g stool, no p-values provided) | | • No resistance of *Escherichia coli* to cefotaxime | • Longitudinal sampling  • Analysis on species level  • Description of study participants ns  • Age distribution of cohort ns  • Sex distribution of cohort ns  • Reason for ABX ns  • Duration of cefotaxime administration ns  • Dose of gentamicin ns  • Frequency of gentamicin ns  • Duration of gentamicin ns  • Route of administration of gentamicin ns  • Storage conditions ns  • Exact timing of stool testing ns  • No molecular diagnostics  • p-values ns |
|  |  |  |  |  | **Genus**  Group D streptococcus (ns) | **Species**  *Escherichia coli* (ns) |  |  |
| Mathieu *et al.* (24)  1979  France  ns, prospective cohort study (2b) | 27 (6, 21)  ns (ns, ns)  Hospitalised children receiving ABX for sepsis, respiratory infections, ENT infection  ns  ns (range 2 d to 9 y)  **Amikacin (plus ampicillin, colistin and others)**  Amikacin 15 mg/kg/d, divided into 3 doses, ns  Intramuscular  ns, | Stool, ns  Culture for *Escherichia coli, Klebsiella, Enterobacteria, Proteus, Pseudomonas, Staphylococcus, Enterococcus faecalis,* anaerobic bacteria  ns  ns, ns, ns  Limit of sensitivity 10² CFU/g stool | Before and after ABX (ns) | • ns  • ns | **Total bacterial count before ABX vs. during ABX** (no p-values provided) | | • ns  • - | • Longitudinal sampling  • Analysis on species level  • Sample numbers ns  • Sex distribution of cohort ns  • Reason for ABX ns  • No information on interval without ABX before inclusion  • Storage conditions ns  • Temperature of incubation ns  • Duration of incubation ns  • No molecular diagnostics  • No comparison between ABX group and controls  • p-values ns |
|  |  |  |  |  | **Genus**  *Enterobacter*  **Species**  *Klebsiella pneumoniae* | **Species**  *Escherichia coli* |  |  |
| Bourrillon *et al.* (37)  1978  France  Prospective cohort study (2b) | 62 (41, 21)  ns (ns, ns)  Hospitalised children receiving ABX (reason ns), hospitalised children not receiving ABX as controls  ns  ns (<1 m 23, 1 m-1 y 17, >1 y 22)  ns | Stool, ns  Culture for *Escherichia coli, Klebsiella, Enterobacteria, Proteus, Pseudomonas, Staphylococcus, Enterococcus faecalis,* anaerobic bacteria  ns  ns, ns, ns  Limit of sensitivity 10² CFU/g stool | Every 3 d during hospitalisation |  | **Total bacterial count before ABX vs. during ABX** (log₁₀ CFU/g stool) | | • ns  • - | • Longitudinal sampling  • Analysis on species level  • Sample numbers ns  • Sex distribution of cohort ns  • Reason for ABX ns  • No information on interval without ABX before inclusion  • Short follow-up⁷  • Storage conditions ns  • Temperature of incubation ns  • Duration of incubation ns  • No molecular diagnostics  • p-values ns |
|  | **Colistin plus gentamicin or amikacin** (27)  50-100,000 IU/kg/d, divided into 3 doses, ns  Oral/intravenous, gentamicin intramuscular  ns |  |  | • No difference in bacterial counts between ABX group and controls  • ns | No difference | No difference |  |  |
|  | **Pristinamycin plus gentamicin** (6)  50-100 mg/kg/d, divided into 3 doses, ns  Oral, gentamicin intramuscular  ns |  |  | • ns  • ns | **Genus**  *Pseudomonas* (ns) | **Genus**  *Staphylococcus* (ns)  **Species**  *Enterococcus faecalis* (ns)  *Escherichia coli* (ns) |  |  |
|  | **Ampicillin plus gentamicin** (n=17)  50-300 mg/kg/d divided into 3-6 doses, ns  Oral, gentamicin intramuscular  Ns |  |  | • ns  • ns | **Genus**  *Klebsiella* (10^5^/10^8^)  **Species**  *Enterococcus faecalis* (ns) | **Species**  *Escherichia coli* (ns) |  |  |
| Goldmann *et al.* (56)  1978  USA  Single centre,  prospective cohort study (2b) | 63 (51, 12)  ns  Hospitalised infants receiving ABX (reason ns), hospitalised infants not receiving ABX as controls  ns  ns  **Various antibiotics**  ns, ns, 67% > 3 d  ns  ns  ns | Stool/rectal swabs, ns  Culture for *Escherichia coli, Klebsiella, Enterobacter, Citrobacter, Pseudomonas, Acinetobacter, Neisseria, Staphylococcus aureus, Staphylococcus epidermidis,* Alpha streptococcus*, Enterococcus,* Group B streptococcus  Trypticase soy broth, sheep blood agar, chocolate agar, MacConkey agar  5% CO_2_, 35°C, 48 h | 0 d, then every 3 d during hospitalisation (mean 17 d) | • ns  • ns | **Colonisation rates ABX group vs. controls** | | • ns  • - | • Longitudinal sampling  • Sample numbers ns  • Age distribution of cohort ns  • Sex distribution of cohort ns  • Reason for ABX ns  • Dose of ABX ns  • Frequency of ABX ns  • Duration of ABX ns  • Route of administration of ABX ns  • No information on interval without ABX before inclusion  • Short follow-up⁷  • Storage conditions ns  • No separate analysis for different ABX  • Only investigated colonisation rates, not abundance  • No molecular diagnostics  • No analysis on species level |
|  |  |  |  |  | **Genus**  *Klebsiella*  *Enterobacter*  *Citrobacter* |  |  |  |
| Moustardier *et al.* (112)  1965  France  Multi-centre, prospective control study (2b) | 55 (-,-)  ns (-,-)  Hospitalised and non-hospitalised children receiving ABX (reason ns)  ns  ns | Stool, ns  Culture  ns  ns, ns, ns | Before and after ABX (ns) | • ns  • ns | **Total bacterial count before vs. after ABX** (no p-values provided) | | • ns  • - | • Longitudinal sampling  • Sample numbers ns  • No control group  • Age distribution of cohort ns  • Sex distribution of cohort ns  • Reason for ABX ns  • Dose of ABX ns (streptomycin, chloramphenicol, tetracyclines, erythromycin)  • Frequency of ABX ns  • Route of administration of ABX ns  • No information on interval without ABX before inclusion  • Storage conditions ns  • Culture medium ns  • Temperature of incubation ns  • Duration of incubation ns  • No molecular diagnostics  • p-values ns |
|  | **Penicillin** (n=8)  200000-800000 U.O./d, ns, 4-8 d  ns  ns  ns |  |  |  | No difference | No difference |  |  |
|  | **Streptomycin** (n=10)  ns, ns, 3-30 d  ns  ns  ns |  |  |  | **Genus**  *Enterococcus*  *Staphylococcus* | **Genus**  *Proteus* |  |  |
|  | **Chloramphenicol** (n=17)  ns, ns, 3-30 d  ns  ns  ns |  |  |  | **Genus**  *Enterococcus*  *Staphylococcus* | **Genus**  *Proteus* |  |  |
|  | **Tetracyclines** (n=10)  ns, ns, ns  ns  ns  ns |  |  |  | **Genus**  *Enterococcus*  *Staphylococcus* | **Genus**  *Proteus*  **Species**  *Escherichia coli* |  |  |
|  | **Erythromycin** (n=10)  ns, ns, ns  ns  ns  ns |  |  |  | **Genus**  *Enterococcus*  *Staphylococcus* | **Genus**  *Proteus* |  |  |
| Brignoli *et al.* (111)  1958  Italy  Multi-centre, prospective control study (2b) | 12 (-,-)  ns (-,-)  Hospitalised children receiving ABX (reason ns)  ns  ns (range 3 d to 8 m)  **Spiramycin**  50-72 mg/kg/d, divided into 3 doses, 6 d  ns  ns  ns | Stool, sterile tube, -4°C  Culture for various gram-positive and gram-negative bacteria  Drigalski agar, Sabouraud’s agar, 1%-Glucose agar  Anaerobic, 37°C, 24-48 h | d 2, 4, 6 during ABX, d 2, 4, 6 after ABX | • ns  • ns | **Total bacterial count before vs. after ABX** (no p-values provided) | | • ns  • - | • Longitudinal sampling  • No control group  • Sample numbers ns  • Sex distribution of cohort ns  • Route of administration of ABX ns  • No information on interval without ABX before inclusion  • No molecular diagnostics  • p-values ns |
|  |  |  |  |  | **Genus**  *Enterococcus*  *Paracolobactrum*  *Micrococcus*  **Species**  *Alcaligenes faecalis*  *Escherichia coli*  *Enterobacter aerogenes*  *Bacillus subtilis* | **Genus**  *Proteus*  **Species**  *Escherichia coli* |  |  |

¹ Large cohort: > 500 children

² Small cohort: < 50 children

³ Large sample number: > 500 samples

⁴ Low sample number: < 50 samples

⁵ Overlapping children

⁶ Long follow up: > 1 y

⁷ Short follow up: ≤ 1 m

⁸ Long interval between ABX and first sampling: ≥6 m

⁹ Short interval without ABX before inclusion: < 1 m without other ABX before inclusion

| ALL – acute lymphoblastic leukaemia | OTU – operational taxonomic unit |
| --- | --- |
| ARG – antibiotic resistance gene(s) | PCR/qPCR – polymerase chain reaction/quantitative polymerase chain reaction |
| ABX – antibiotic treatment | PERMANOVA - Permutational multivariate analysis of variance |
| d – day(s) | RDP – ribosomal database project |
| GA – gestational age | rRNA – ribosomal ribonucleic acid |
| IQR – interquartile range | RT/RCT – randomised trial/randomised controlled trial |
| m – month(s) | RSV – respiratory syncytial virus |
| NCBI – National Center for Biotechnology Information | SD – standard deviation |
| NEC – necrotising enterocolitis | w – week(s) |
| ns – not specified | y – year(s) |

**Supplementary table 2** Analysis of the risk of bias of the included studies.

| Author | Selection bias | Performance  bias | Confounding | Detection bias | Attrition bias | Inappropriate/  no statistical analysis |
| --- | --- | --- | --- | --- | --- | --- |
| Hutchinson *et al.* (64) | - | - | - | - | - | - |
| Li *et al.* (75) | - | - | - | - | - | - |
| Schwartz *et al*. (89) | - | ns | - | - | + | - |
| Barnett *et al.* (29) | + | - | - | - | + | - |
| Reyman *et al.* (83) | + | - | - | - | + | - |
| Kwon *et al*. (72) | - | - | + | - | + | - |
| Lebeaux *et al.* (74) | + | - | + | - | + | - |
| Xu *et al.* (97) | - | - | - | - | - | - |
| Ainonen *et al.* (26) | - | - | - | - | + | - |
| Bender *et al.* (30) | - | - | - | - | + | - |
| Chang *et al.* (40) | - | - | - | - | - | - |
| Kim et al. (69) | - | - | ns | - | ns | - |
| Gong *et al.* (57) | - | ns | - | - | ns | - |
| Russell *et al*. (86) | - | - | - | - | + | - |
| Akagawa et al. (27) | - | - | - | - | - | - |
| Lindberg *et al.* (76) | - | ns | - | - | ns | - |
| Eck *et al.* (48) | - | - | - | - | - | - |
| Jia *et al.* (68) | - | - | - | - | - | - |
| Lu *et al.* (77) | - | - | - | - | - | - |
| Rooney *et al.* (85) | - | - | - | + | - | - |
| Hinterwirth *et al.* (62) | - | - | - | - | - | - |
| Zwittink *et al.* (102) | - | ns | - | - | + | - |
| Korpela *et al.* (71) | - | ns | - | - | - | - |
| Gasparrini *et al.* (54) | - | - | - | - | - | - |
| Bourke *et al.* (36) | - | - | - | - | + | - |
| D`Souza *et al.* (47) | - | ns | + | - | - | - |
| D’Agata *et al.* (42) | - | ns | - | - | - | - |
| Fouhy *et al.* (51) | - | - | - | - | + | - |
| D’Haens et al. (45) | - | - | ns | - | ns | - |
| Doan *et al.* (103) | - | - | - | - | - | - |
| Oldenburg *et al.* (79) | - | + | - | - | - | - |
| Wei *et al.* (95) | + | - | - | - | ns | - |
| Tapiainen *et al.* (92) | - | - | - | - | - | - |
| Dahl et al. (43) | - | ns | - | - | ns | - |
| Doan *et al.* (46) | - | - | - | - | - | - |
| Parker *et al.* (80) | - | - | - | - | - | - |
| Zwittink *et al.* (101) | - | - | ns | - | - | - |
| Forsgren *et al.* (50) | - | - | - | - | - | - |
| Itani *et al.* (65) | - | ns | - | - | + | - |
| Zhu et al. (100) | - | - | - | - | ns | - |
| Korpela *et al.* (70) | **-** | **-** | **-** | **-** | **-** | **-** |
| Romano-K. *et al.* (84) | - | - | - | - | ns | - |
| Gibson *et al.* (55) | - | - | - | - | - | - |
| Chernikova *et al.* (41) | + | - | - | - | + | - |
| Bokulich *et al.* (115) | - | - | - | - | + | - |
| Arboleya *et al.* (28) | - | - | + | - | - | - |
| Yassour *et al.* (98) | - | - | - | - | - | - |
| Ward *et al.* (94) | - | - | - | - | + | - |
| Zhou et al. (99) | - | - | - | - | - | - |
| Dardas *et al.* (44) | - | ns | - | - | + | - |
| Greenwood *et al.* (58) | - | ns | - | - | - | - |
| La Rosa *et al.* (73) | - | ns | - | - | - | - |
| Jenke *et al.* (67) | - | ns | - | - | - | - |
| Ferraris *et al.* (49) | - | - | - | - | - | - |
| Westerbeek *et al.* (104) | - | ns | - | - | - | - |
| Jacquot *et al.* (66) | - | + | - | - | + | - |
| Savino *et al.* (88) | - | - | + | ns | - | - |
| Tanaka *et al.* (91) | - | - | - | - | ns | - |
| Fouhy *et al.* (52) | - | ns | - | - | ns | - |
| Mangin *et al.* (78) | - | ns | + | - | - | - |
| Parm *et al.* (81) | - | + | - | ns | - | - |
| Butel *et al*. (39) | - | - | - | - | ns | - |
| Penders *et al.* (82) | + | - | - | - | ns | - |
| Bonnemaison *et al.* (34) | - | - | - | + | + | - |
| Hall *et al.* (61) | - | - | - | + | + | - |
| Holton et al. (63) | - | + | - | - | - | - |
| Tullus *et al.* (93) | - | ns | - | + | - | - |
| Bennet *et al.* (32) | - | - | - | - | - | - |
| Bennet *et al.* (31) | - | - | - | - | + | + |
| Butel *et al.* (38) | - | - | + | - | - | + |
| Welkon *et al.* (96) | - | - | - | + | - | + |
| Borderon *et al.* (105) | - | - | + | - | + | - |
| Sakata *et al.* (87) | - | ns | + | - | - | - |
| Guggenbichler *et al.* (60) | - | - | - | + | - | + |
| Borderon *et al.* (35) | - | - | + | + | - | - |
| Lambert-Z. *et al.* (107) | - | - | + | - | - | + |
| Lambert-Z. *et al.* (106) | - | - | + | - | - | + |
| Fujita *et al.* (53) | + | - | + | - | - | - |
| Sunakawa *et al.* (90) | - | - | + | - | - | + |
| Blakey *et al.* (33) | - | ns | - | - | - | + |
| Grylack et al. (59) | - | - | - | + | - | + |
| Bingen *et al.* (110) | - | - | + | - | - | + |
| Lambert-Z. *et al.* (109) | - | - | + | - | - | + |
| Lambert-Z *et al.* (108) | - | - | + | - | - | + |
| Mathieu et al. (24) | - | - | + | - | - | + |
| Bourrillon *et al.* (37) | - | - | + | - | - | + |
| Goldmann *et al.* (56) | - | - | - | + | - | + |
| Moustardier *et al.* (112) | ns | ns | ns | - | - | + |
| Brignoli *et al.* (111) | ns | - | - | - | - | + |

ns – not specified

**Supplementary table 3** Summary of characteristics and methods applied across studies. The studies are grouped by the antibiotic groups.

| **ABX group**  **Studies (S)**  **Participants (P)**  **Age distribution (A)**  **Follow-up (F)** | **ABX investigated** | **Microbiota analysis method** | **Reason for ABX** |
| --- | --- | --- | --- |
| **Penicillins**  S: 13  P: 1,311  A: Preterm birth to 7 y of age  F: d 4 of ABX to 2 y after stopping ABX | • Penicillin and ampicillin (55,75,87,90,112)  • Amoxicillin (71,78,79,89)  • Amoxicillin plus clavulanate (35,107)  • Penicillin and amoxicillin with or without clavulanate (70)  • Penicillin plus moxalactam (100)  • Piperacillin-tazobactam (100) | • 16S rRNA gene sequencing (55,70,71,79,100)  • Metagenomic sequencing (75,89)  • Cultures (35,87,90,107,112)  • PCR-TGGE (78) | • Respiratory infections (71,78)  • Severe acute malnutrition (89)  • Various other infections (35,55,70,75,87,107)  • Healthy children (79)  • ns (90,100,112) |
| **Penicillins plus aminoglycosides**  S: 13  P: 3,141  A: Preterm birth to 12 m of age  F: 2 w to 12 m | • Ampicillin plus gentamicin (37,40,52,58,69,81,93,94,110)  • Penicillin plus gentamicin (81,83)  • Penicillin plus tobramycin (26,85)  • Amoxicillin plus clavulanate plus gentamicin (83)  • Penicillin plus netilmicin (63) | •16S rRNA gene sequencing (26,40,46,52,58,69,83)  • Cultures (37,63,81,93,110)  • Shotgun metagenomic sequencing (94) | • (Suspected) early onset sepsis (63,81,83)  • Suspected other infections (40,85)  • Necrotizing enterocolitis (58,94)  • ns (26,37,52,93,110) |
| **Penicillins plus cephalosporins**  S: 5  P: 1,123  A: Preterm birth to 12 months of age  F: 1 w to 12 m | • Amoxicillin plus cefotaxime (83)  • Ampicillin plus cefotaxime (40,85)  • Amoxicillin plus ceftazidime (101)  • Amoxicillin plus cefotaxime plus netilmicin (34) | • 16S rRNA gene sequencing sequencing (40,83,85,101)  • Cultures (34) | • Suspected early onset sepsis (83)  • Suspected or proven other infections (34,40,101), (85) |
| **Cephalosporins**  S: 9  P: 916  A: Preterm birth to 18 m of age  F: 6 d to 10 m | • Cefalexin (91)  • Cefazolin (55)  • Cefuroxime (93)  • Cefaclor (87)  • Cefotaxime (55,57,63)  • Ceftriaxone (88)  • Cefpiramide (87)  • Ceftazidime (87) | • 16S rRNA gene sequencing (55,57,91)  • Cultures (60,63,87,88,90,93) | • Various infections (55,57,60,87,88)  • Suspected sepsis (63) |
| **Carbapenems**  S: 3  P: 67  A: Preterm birth to 15 y of age  F: End of ABX to 3 d after ABX | • Imipenem-cilastatin (96,105)  • Meropenem (55) | • Cultures (96,105)  • Shotgun metagenomic sequencing (55) | • Various infections (all studies) |
| **Macrolides**  S: 13  P: 802  A: Birth to 15 y of age  F: 2 d to 10 m after stopping ABX | • Erythromycin (38,87,112)  • Azithromycin (46,62,79,80,95,103)  • Spiramycin (111)  • ns or no separate analysis for different macrolides (70,71,75) | • 16S rRNA gene sequencing (46,70,71,79,80,95)  • Shotgun metagenomic sequencing sequencing (62,79)  • Cultures (38,87,111,112) | • Various infections (70,75,87)  • Respiratory infections (71)  • Asthma-like symptoms (95)  • Healthy children (46,62,79,80,103)  • ns (39,111,112) |
| **Trimethoprim/**  **sulfamethoxazole**  S: 6  P: 254  A: Preterm birth to 8 y of age  F: 2 d after ABX to 8 y after randomisation | • na | • 16S rRNA gene sequencing (27,55,79)  • Shotgun metagenomic sequencing (36,47)  • Cultures (53) | • Various infections (53,55)  • HIV-positive children receiving prophylactic ABX (36)  • HIV-exposed, uninfected infants receiving prophylactic ABX (47)  • Children with vesicoureteral reflux receiving prophylactic ABX (27)  • Healthy children (79) |
| **Aminoglycosides**  S: 3  P: 52  A: Preterm birth to 6 y  F: 2 d to 3-6 d after ABX | • Gentamycin (55,87)  • Steptomycin (112) | • 16S rRNA gene sequencing (55)  • Cultures (87) | • Various infections (55,87)  • ns (112) |
| **Glycopeptides**  S: 3  P: 48  A: Preterm neonates (all studies)  F: 2 d after ABX to 6 w of life | • Vancomycin (all studies) | • 16S rRNA gene sequencing (all studies) | • Necrotizing enterocolitis (84)  • Various infections (55)  • Prophylaxis for central venous line-associated sepsis (45) |
| **Various ABX**  S: 38  P: 5,139  A: Preterm birth to 5 y  F: 4 d to 21 m after ABX | • No separate analysis for different ABX (24,31–33,44,48,49,54,59,64–66,72–74,77,86,92,97)  • ABX ns (28–30,38,42,43,50,51,56,61,67,68,76,82,98,99,104,106,109,115) | • 16S rRNA gene sequencing (28,29,42–44,51,64,66–68,72–74,76,77,86,92,99,115)  • Shotgun metagenomic sequencing (30,54,74,97,98)  • Cultures (24,31–33,49,56,59,61,106,109)  • qPCR (50,65,67,82)  • PCR-TGGE (38,49)  • FISH (104)  • 16S-23S IS profiling (48) | • Various (suspected) infections (24,31,32,40,65,72,74,86,98,106)  • Suspected neonatal sepsis (48)  • Suspected necrotizing enterocolitis (59,67,76)  • ns (28,29,33,39,42–44,49–51,54,56,61,64,66,68,73,77,82,92,97,99,104,109,115) |

ABX – antibiotic treatment

FISH – Fluorescence in situ hybridization

HIV – human immunodeficiency viruses

ns – not specified

na – not applicable

PCR/qPCR/PCR-TGGE – polymerase chain reaction/quantitative polymerase chain reaction/temperature gradient gel electrophoresis

rRNA – ribosomal ribonucleic acid

**Pubmed Search Term**

((("Anti-Bacterial Agents/administration and dosage"[Majr] OR "Anti-Bacterial Agents/adverse effects"[Majr] OR "Anti-Bacterial Agents/pharmacology"[Majr] OR "Anti-Bacterial Agents/therapeutic use"[Majr] OR "Anti-Infective Agents/administration and dosage"[Majr:NoExp] OR "Anti-Infective Agents/adverse effects"[Majr:NoExp] OR "Anti-Infective Agents/pharmacology"[Majr:NoExp] OR "Anti-Infective Agents/therapeutic use"[Majr:NoExp] OR "Tetracyclines/administration and dosage"[Majr] OR "Tetracyclines/adverse effects"[Majr] OR "Tetracyclines/pharmacology"[Majr] OR "Tetracyclines/therapeutic use"[Majr] OR "Lactams/administration and dosage"[Majr:NoExp] OR "Lactams/adverse effects"[Majr:NoExp] OR "Lactams/pharmacology"[Majr:NoExp] OR "Lactams/therapeutic use"[Majr:NoExp] OR "Beta-lactams/administration and dosage"[Majr] OR "Beta-lactams/adverse effects"[Majr] OR "Beta-lactams/pharmacology"[Majr] OR "Beta-lactams/therapeutic use"[Majr] OR "Amoxicillin/administration and dosage"[Majr] OR "Amoxicillin/adverse effects"[Majr] OR "Amoxicillin/pharmacology"[Majr] OR "Amoxicillin/therapeutic use"[Majr] OR "Quinolones/administration and dosage"[Majr:NoExp] OR "Quinolones/adverse effects"[Majr:NoExp] OR "Quinolones/pharmacology"[Majr:NoExp] OR "Quinolones/therapeutic use"[Majr:NoExp] OR "Fluoroquinolones/administration and dosage"[Majr] OR "Fluoroquinolones/adverse effects"[Majr] OR "Fluoroquinolones/pharmacology"[Majr] OR "Fluoroquinolones/therapeutic use"[Majr] OR "Macrolides/administration and dosage"[Majr] OR "Macrolides/adverse effects"[Majr] OR "Macrolides/pharmacology"[Majr] OR "Macrolides/therapeutic use"[Majr] OR "Sulfamethoxazole/administration and dosage"[Majr:NoExp] OR "Sulfamethoxazole/adverse effects"[Majr:NoExp] OR "Sulfamethoxazole/pharmacology"[Majr:NoExp] OR "Sulfamethoxazole/therapeutic use"[Majr:NoExp] OR "Trimethoprim, Sulfamethoxazole Drug Combination/administration and dosage"[Majr:NoExp] OR "Trimethoprim, Sulfamethoxazole Drug Combination/adverse effects"[Majr:NoExp] OR "Trimethoprim, Sulfamethoxazole Drug Combination/pharmacology"[Majr:NoExp] OR "Trimethoprim, Sulfamethoxazole Drug Combination/therapeutic use"[Majr:NoExp] OR "Trimethoprim/administration and dosage"[Majr:NoExp] OR "Trimethoprim/adverse effects"[Majr:NoExp] OR "Trimethoprim/pharmacology"[Majr:NoExp] OR "Trimethoprim/therapeutic use"[Majr:NoExp] OR "Aminoglycosides/administration and dosage"[Majr] OR "Aminoglycosides/adverse effects"[Majr] OR "Aminoglycosides/pharmacology"[Majr] OR "Aminoglycosides/therapeutic use"[Majr]) OR (antibiotic[tiab] OR antibiotics[tiab] OR "antibacterial agent"[tiab:~1] OR "antibacterial agents"[tiab:~1] OR "anti-bacterial agent"[tiab:~1] OR "anti-bacterial agents"[tiab:~1] OR "bacteriocide agent"[tiab:~1] OR "bacteriocide agents"[tiab:~1] OR "antimicrobial agent"[tiab:~1] OR "antimicrobial agents"[tiab:~1] OR "anti-microbial agent"[tiab:~1] OR "anti-microbial agents"[tiab:~1] OR "antimyrobial agent"[tiab:~1] OR "antimyrobial agents"[tiab:~1] OR "anti-myrobial agent"[tiab:~1] OR "anti-myrobial agents"[tiab:~1] OR "antiinfective agent"[tiab:~1] OR "antiinfective agents"[tiab:~1] OR "anti-infective agent"[tiab:~1]OR "anti-infective agents"[tiab:~1] OR "antitubercular agent"[tiab:~1] OR "antitubercular agents"[tiab:~1] OR "anti-tubercular agent"[tiab:~1] OR "anti-tubercular agents"[tiab:~1]) OR (aminoglycoside*[tiab] OR penicillin*[tiab] OR amoxcillin*[tiab] OR amoxillin*[tiab] OR amoxycillin*[tiab] OR amoxicillin*[tiab] OR carbapenem*[tiab] OR cephalosporin*[tiab] OR macrolide*[tiab] OR macrotetrolide*[tiab] OR quinolone*[tiab] OR chinolone[tiab] OR tetracyclin*[tiab] OR chlortetracyclin*[tiab] OR chlortetracylin*[tiab] OR demeclocyclin*[tiab] OR mexocine*[tiab] OR doxycyclin*[tiab] OR doxymycin*[tiab] OR metacyclin*[tiab] OR methacyclin*[tiab] OR minocyclin*[tiab] OR minocyn*[tiab] OR oxytetracyclin*[tiab] OR rolitetracyclin*[tiab] OR tetracyn*[tiab] OR topicyclin*[tiab] OR trimethoprim*[tiab] OR trimetoprim*[tiab] OR trimethoprin*[tiab] OR fluoroquinolone*[tiab] OR cotrimoxazole*[tiab] OR bactrim*[tiab] OR sulfamethoprim*[tiab] OR sulfamethoxazole*[tiab] OR beta-lactam*[tiab] OR Glycopeptide*[tiab] OR Tigecycline*[tiab] OR Streptogramin*[tiab] OR Linezolid*[tiab] OR Colistin*[tiab] OR Sulfonamide*[tiab] OR Nitrofurantoin*[tiab] OR Fosfomycin*[tiab] OR alatrofloxacin*[tiab] OR amikacin*[tiab] OR ampicillin*[tiab] OR amoxicillin-clavulanate*[tiab] OR clavulanate*[tiab] OR clavulanic*[tiab] OR atovaquone*[tiab] OR azithromycin*[tiab] OR azlocillin*[tiab] OR aztreonam*[tiab] OR benzylpenicillin*[tiab] OR benzathine-benzylpenicillin*[tiab] OR cefaclor*[tiab] OR cefalexin*[tiab] OR cephalexin*[tiab] OR cefalotin*[tiab] OR cephalothin*[tiab] OR cefamandole*[tiab] OR cefazolin*[tiab] OR cephazolin*[tiab] OR cefepime*[tiab] OR cefodizime*[tiab] OR cefotaxime*[tiab] OR cefotetan*[tiab] OR cefoxitin*[tiab] OR cefpirome*[tiab] OR cefpodoxime*[tiab] OR ceftaroline*[tiab] OR ceftazidime*[tiab] OR ceftolozane*[tiab] OR ceftriaxone*[tiab] OR cefuroxime*[tiab] OR chloramphenicol*[tiab] OR ciprofloxacin*[tiab] OR clarithromycin*[tiab] OR clindamycin*[tiab] OR cloxacillin*[tiab] OR colistimethate*[tiab] OR dalfopristin*[tiab] OR quinupristin*[tiab] OR daptomycin*[tiab] OR dicloxacillin*[tiab] OR doripenem*[tiab] OR enoxacin*[tiab] OR ertapenem*[tiab] OR erythromycin*[tiab] OR fidaxomicin*[tiab] OR fleroxacin*[tiab] OR flucloxacillin*[tiab] OR fosfomycin-trometamol*[tiab] OR fusidic-acid*[tiab] OR gatifloxacin*[tiab] OR gentamicin*[tiab] OR gentamycin*[tiab] OR imipenem*[tiab] OR lincomycin*[tiab] OR loracarbef*[tiab] OR meropenem*[tiab] OR metronidazole*[tiab] OR mezlocillin*[tiab] OR micafungin*[tiab] OR moxifloxacin*[tiab] OR nalidixic-acid*[tiab] OR norfloxacin*[tiab] OR ofloxacin*[tiab] OR oseltamivir*[tiab] OR pentamidine*[tiab] OR permethrin*[tiab] OR phenoxymethylpenicillin*[tiab] OR piperacillin*[tiab] OR procaine-benzylpenicillin*[tiab] OR rifampicin*[tiab] OR rifaximin*[tiab] OR roxithromycin*[tiab] OR spectinomycin*[tiab] OR tazobactam*[tiab] OR teicoplanin*[tiab] OR ticarcillin*[tiab] OR tinidazole*[tiab] OR trovafloxacin*[tiab] OR vancomycin*[tiab] OR nitroimidazole*[tiab] OR lipoglycopeptide*[tiab] OR oxazolidinone*[tiab] OR rifamycin*[tiab] OR sarecycline*[tiab] OR relebactam*[tiab] OR rifampin*[tiab] OR benznidazole*[tiab] OR besifloxacin*[tiab] OR dalbavancin*[tiab] OR fexinidazole*[tiab] OR oritavancin*[tiab] OR omadacycline*[tiab] OR bedaquiline*[tiab] OR tedizolid*[tiab] OR telavancin*[tiab] OR ozenoxacin*[tiab] OR eravacycline*[tiab] OR finafloxacin*[tiab] OR plazomicin*[tiab] OR chloromycetin*[tiab] OR vibramycin*[tiab] OR levofloxacin*[tiab] OR lymecycline*[tiab] OR terramycin*[tiab] OR spiramycin*[tiab] OR polymyxin*[tiab] OR isoniazid*[tiab] OR ethambutol*[tiab] OR suphonamide*[tiab])) AND ("Genetic Predisposition to Disease"[Mesh:NoExp] OR "Environmental Exposure"[Mesh:NoExp] OR "Prenatal Exposure Delayed Effects"[Mesh:NoExp] OR use[tiab] OR exposure[tiab] OR expose*[tiab] OR early[tiab] OR first-year*[tiab] OR second-year[tiab] OR year-of-life[tiab] OR years-of-life[tiab] OR change*[tiab] OR effect*[tiab] OR impact*[tiab]) AND (newborn* OR new-born* OR baby OR babies OR neonat* OR neo-nat* OR infan* OR toddler* OR pre-schooler* OR preschooler* OR kinder OR kinders OR kindergarten* OR boy OR boys OR girl OR girls OR child OR children OR childhood OR pediatric* OR paediatric* OR adolescen* OR youth OR youths OR teen OR teens OR teenage* OR school-age* OR schoolage* OR school-child* OR schoolchild* OR school-girl* OR schoolgirl* OR school-boy* OR schoolboy* OR young-adult* OR young-people* OR young-person* OR emerging-adult* OR emerging-person* OR AYA OR AYAs OR CAYA OR CAYAs) AND ("Bacteria/drug effects"[Majr:NoExp] OR "Bacteria/genetics"[Majr:NoExp] OR "Microbiota/drug effects"[Majr:NoExp] OR "Gastrointestinal Microbiome/drug effects"[Majr:NoExp] OR "Intestinal Mucosa/drug effects"[Majr:NoExp] OR "Intestinal Mucosa/microbiology"[Majr:NoExp] OR "Feces/microbiology"[Mesh:NoExp] OR "Gastrointestinal Tract/drug effects"[Mesh:NoExp] OR "Gastrointestinal Tract/microbiology"[Mesh:NoExp] OR "Intestines/microbiology"[Majr:NoExp] OR microbiota*[tiab] OR micro-biota*[tiab] OR microbiome*[tiab] OR micro-biome*[tiab] OR microflora*[tiab] OR microbial-diversity[tiab] OR gut-flora[tiab] OR intestinal-flora[tiab] OR bacterial-composition[tiab] OR microbial-community[tiab] OR gut-resistome[tiab] OR bacterial-flora[tiab] OR intestine-flora[tiab] OR gastrointestinal-tract[tiab] OR gastro-intestinal-tract[tiab])) NOT (("Animal" OR "animals" OR "rat" OR "rats" OR "mouse" OR "mice" OR "rodent*" OR "swine" OR "porcine" OR "murine" OR "sheep" OR "lamb" OR "lambs" OR "pig" OR "pigs" OR "piglet" OR "piglets" OR "rabbit" OR "rabbits" OR "cat" OR "cats" OR "dog" OR "dogs" OR "cattle" OR "bovine" OR "monkey" OR "monkeys" OR "trout" OR "marmoset" OR "marmosets") NOT ("human" OR "humans" OR "patient" OR "patients" OR "newborn*" OR "baby" OR "babies" OR "neonat*" OR "infan*" OR "toddler*" OR "pre-schooler*" OR "preschooler*" OR "kindergarten" OR "boy" OR "boys" OR "girl" OR "girls" OR "child" OR "children" OR "childhood" OR "adolescen*" OR "pediatric*" OR "paediatric*" OR "youth*" OR "teen" OR "teens" OR "teenage*" OR "school-aged*" OR "school-child*" OR "school-girl*" OR "school-boy*" OR "schoolgirl*" OR "schoolboy*" OR "man" OR "men" OR "woman" OR "women" OR "adult" OR "adults" OR "middle-age*" OR "elderly"))

**Embase Search Term**

1 exp antibiotic agent/ct, cb, cm, do, dt, iv, po, pv, pk, pd

2 *antiinfective agent/ct, cb, cm, do, dt, iv, po, pv, pk, pd or exp *leprostatic agent/ct, cb, cm, do, dt, po, pk, pd, pv

3 *tetracycline/ct, cb, cm, do, dt, iv, po, pv, pk, pd

4 exp *beta lactam antibiotic/ct, cb, cm, do, dt, iv, po, pk, pd, pv or exp *beta lactamase inhibitor/ct, cb, cm, do, dt, iv, po, pk, pd, pv

5 *amoxicillin/ct, cb, cm, do, dt, iv, po, pk, pd, pv

6 exp *quinolone derivative/ct, cb, cm, do, dt, iv, po, pk, pd, pv

7 exp *macrolide/ct, cb, cm, do, dt, iv, po, pk, pd, pv

8 *sulfamethoxazole/ct, cb, cm, do, dt, iv, po, pk, pd, pv or *cotrimoxazole/ct, cb, cm, do, dt, iv, po, pk, pd, pv

9 *trimethoprim/ct, cb, cm, do, dt, iv, po, pk, pd, pv or exp *aminoglycoside antibiotic agent/ct, cb, cm, do, dt, iv, po, pk, pd, pv

10 (antibiotic or antibiotics or ((antibacterial* or anti-bacterial* or bacteriocid* or antimicrobial* or anti-microbial* or antimycrobial* or anti-mycrobial* or antiinfective* or anti-infective* or antitubercular* or anti-tubercular*) adj1 agent*)).tw,kf,dq.

1 (aminoglycoside* or aminoglycoside or penicillin* or amoxcin* or amoxcillin* or amoxiclin* or amoxillin* or amoxycillin* or amoxicillin* or carbapenem* or cephalosporin* or macrolide* or macrotetrolide* or quinolone* or chinolone or tetracyclin* or chlortetracyclin* or chlortetracylin* or demeclocyclin* or meciclin* or mexacine* or mexocine* or doxycyclin* or doxymycin* or metacyclin* or methacyclin* or minocyclin* or minocyn* or oxytetracyclin* or rolitetracyclin* or tetracyn* or topicyclin* or trimethoprim* or trimetoprim* or trimethoprin* or fluoroquinolone* or cotrimoxazole* or bactrim* or sulfamethoprim* or sulfamethoxazole* or sulfaprim* or beta-lactam* or Glycopeptide* or Tigecycline* or Streptogramin* or Linezolid* or Colistin* or Sulfonamide* or Nitrofurantoin* or Fosfomycin* or alatrofloxacin* or amikacin* or ampicillin* or amoxicillin-clavulanate* or clavulanate* or clavulanic* or atovaquone* or azithromycin* or azlocillin* or aztreonam* or benzylpenicillin* or benzathine-benzylpenicillin* or cefaclor* or cefalexin* or cephalexin* or cefalotin* or cephalothin* or cefamandole* or cefazolin* or cephazolin* or cefepime* or cefodizime* or cefotaxime* or cefotetan* or cefoxitin* or cefpirome* or cefpodoxime* or ceftaroline* or ceftazidime* or ceftolozane* or ceftriaxone* or cefuroxime* or chloramphenicol* or ciprofloxacin* or clarithromycin* or clindamycin* or cloxacillin* or colistimethate* or colistin-IV-with-colistimethate* or dalfopristin* or quinupristin* or daptomycin* or dicloxacillin* or doripenem* or enoxacin* or ertapenem* or erythromycin* or fidaxomicin* or fleroxacin* or flucloxacillin* or fosfomycin-trometamol* or fusidic-acid* or gatifloxacin* or gentamicin* or gentamycin* or imipenem* or lincomycin* or loracarbef* or meropenem* or metronidazole* or mezlocillin* or micafungin* or moxifloxacin* or nalidixic-acid* or norfloxacin* or ofloxacin* or oseltamivir* or pentamidine* or permethrin* or phenoxymethylpenicillin* or piperacillin* or procaine-benzylpenicillin* or rifampicin* or rifaximin* or roxithromycin* or spectinomycin* or tazobactam* or teicoplanin* or ticarcillin* or tinidazole* or trovafloxacin* or vancomycin* or nitroimidazole* or lipoglycopeptide* or oxazolidinone* or rifamycin* or sarecycline* or relebactam* or rifampin* or benznidazole* or besifloxacin* or dalbavancin* or fexinidazole* or oritavancin* or omadacycline* or bedaquiline* or tedizolid* or telavancin* or ozenoxacin* or eravacycline* or finafloxacin* or plazomicin* or chloromycetin* or vibramycin* or levofloxacin* or lymecycline* or terramycin* or spiramycin* or polymyxin* or isoniazid* or ethambutol* or suphonamide*).tw,kf,dq.

12 *antibiotic prophylaxis/

13 1 or 2 or 3 or 4 or 5 or 6 or 7 or 8 or 9 or 10 or 11 or 12

14 *genetic predisposition/

15 *environmental exposure/

16 *prenatal exposure delayed effect/

17 ("use" or exposure or expose* or early or first-year* or second-year or year-of-life or years-of-life or change* or effect* or impact*).tw,kf,dq.

18 14 or 15 or 16 or 17

19 (newborn* or new-born* or baby or babies or neonat* or neo-nat* or infan* or toddler* or pre-schooler* or preschooler* or kinder or kinders or kindergarten* or kinder-aged or boy or boys or girl or girls or child or children or childhood or pediatric* or paediatric* or adolescen* or youth or youths or teen or teens or teenage* or school-age* or schoolage* or school-child* or schoolchild* or school-girl* or schoolgirl* or school-boy* or schoolboy* or young-adult* or young-people* or young-person* or emerging-adult* or emerging-people* or emerging-person* or AYA or AYAs or CAYA or CAYAs).tw,kf,hw,dq.

20 exp *bacterium/

21 *microflora/ or *bacterial flora/ or *feces microflora/

22 exp *intestine flora/

23 *intestine mucosa/

24 *feces/

25 exp *gastrointestinal tract/

26 (microbiota* or micro-biota* or microbiome* or micro-biome* or microflora* or microbial-diversity or gut-flora or intestinal-flora or bacterial-composition or microbial-community or gut-resistome).tw,kf,dq.

27 intestine/

28 20 or 21 or 22 or 23 or 24 or 25 or 26 or 27

29 13 and 18 and 19 and 28

30 (rat or rats or mouse or mice or rodent* or swine or porcine or murine or sheep or lamb or lambs or pig or pigs or piglet or piglets or rabbit or rabbits or cat or cats or dog or dogs or cattle or bovine or monkey or monkeys or trout or marmoset or marmosets).ti. and animal experiment/

31 Animal experiment/ not (human experiment/ or human/)

32 29 not (30 or 31)

33 case report/

34 limit 33 to (conference abstract or conference paper or "conference review" or editorial or letter or "preprint (unpublished, non-peer reviewed)")

35 32 not (33 or 34)
